# Desymmetrization via Activated Esters Enables Rapid Synthesis of Multifunctional Benzene-1,3,5-tricarboxamides and Creation of Supramolecular Hydrogelators

**DOI:** 10.1021/jacs.1c12685

**Published:** 2022-02-23

**Authors:** Shahzad Hafeez, Huey Wen Ooi, Dennis Suylen, Hans Duimel, Tilman M. Hackeng, Clemens van Blitterswijk, Matthew B. Baker

**Affiliations:** † Department of Complex Tissue Regeneration, MERLN Institute for Technology Inspired Regenerative Medicine, Maastricht University, P.O. Box 616, 6200 MD Maastricht, The Netherlands; ‡ Department of Biochemistry, Cardiovascular Research Institute Maastricht (CARIM), Maastricht University, P.O. Box 616, 6200 MD Maastricht, The Netherlands; § Maastricht MultiModal Molecular Imaging Institute (M4i), Maastricht University, P.O. Box 616, 6200 MD Maastricht, The Netherlands

## Abstract

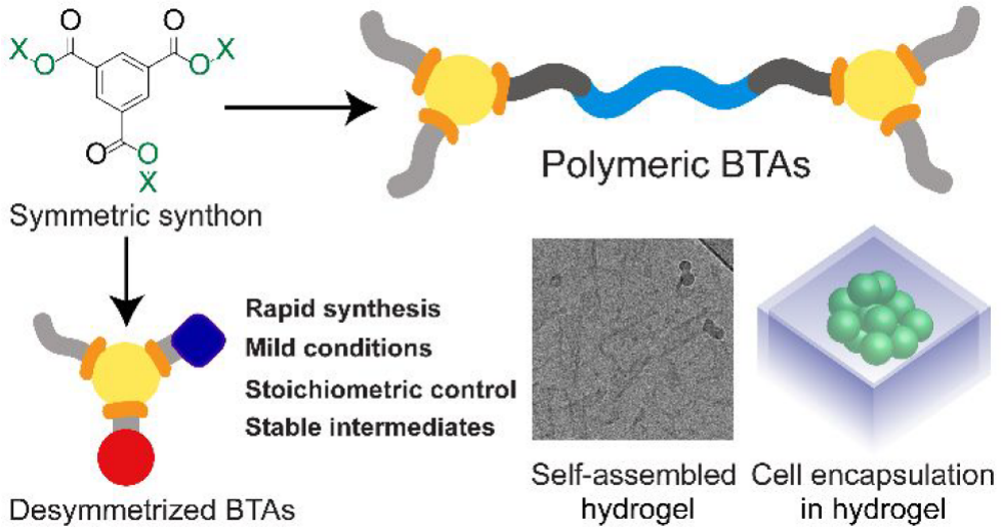

Supramolecular materials based on the self-assembly of benzene-1,3,5-tricarboxamide (BTA) offer an approach to mimic fibrous self-assembled proteins found in numerous natural systems. Yet, synthetic methods to rapidly build complexity, scalability, and multifunctionality into BTA-based materials are needed. The diversity of BTA structures is often hampered by the limited flexibility of existing desymmetrization routes and the purification of multifunctional BTAs. To alleviate this bottleneck, we have developed a desymmetrization method based on activated ester coupling of a symmetric synthon. We created a small library of activated ester synthons and found that a pentafluorophenol benzene triester (BTE) enabled effective desymmetrization and creation of multifunctional BTAs in good yield with high reaction fidelity. This new methodology enabled the rapid synthesis of a small library of BTA monomers with hydrophobic and/or orthogonal reactive handles and could be extended to create polymeric BTA hydrogelators. These BTA hydrogelators self-assembled in water to create fiber and fibrous sheet-like structures as observed by cryo-TEM, and the identity of the BTA conjugated can tune the mechanical properties of the hydrogel. These hydrogelators display high cytocompatibility for chondrocytes, indicating potential for the use of these systems in 3D cell culture and tissue engineering applications. This newly developed synthetic strategy facilitates the simple and rapid creation of chemically diverse BTA supramolecular polymers, and the newly developed and scalable hydrogels can unlock exploration of BTA based materials in a wider variety of tissue engineering applications.

## Introduction

Supramolecular materials offer the ability to build complex and organized materials via directional noncovalent interactions.^1^ Natural systems have evolved to rely on these weak supramolecular interactions to provide complex materials functions based on the reversibility and responsiveness enabled by supramolecular interactions.^2^ However, the chemical diversity of fully synthetic supramolecular molecules and assembled architectures are relatively simple when compared to the natural world. In order to continue the push toward more complex supramolecular materials, new synthetic methodology (molecular complexity) and assembly strategies (supramolecular complexity) are needed.

For example, recapitulating the complexity of the native extracellular matrix (ECM) in a controllable synthetic system is paramount for the control and guidance of cell-based therapies in applications from drug delivery to tissue engineering. Supramolecular hydrogels offer a decidedly biomimetic solution to create an artificial ECM due to their ECM mimicking fibrous structure, physical interactions, dynamics, and mechanical properties.^3,2^ Such supramolecular hydrogels are designed using noncovalent interactions like hydrogen bonding, van der Waals, π–π, and hydrophobic interactions, which mimic the physical interactions found between proteins in the native ECM.^2^ The specificity and directionality of supramolecular interactions have been used for the creation of fibrous structures similar to the ECM^3^ and can enable the tuning of bioactive properties.^4^ Furthermore, the self-organization and specificity of supramolecular interactions make it possible to combine different modules/monomers simply by a mix and match approach to finely tune materials composition, structure, bioactivity, dynamicity, and mechanical properties on the nanoscale.^4−6,1,7−9^

Benezene-1,3,5-tricarboxamide (BTA) is a promising supramolecular synthon due the supramolecular fibril structure in the assembled state, the potential to design multifunctional and multiarm derivatives for increased complexity, and known structure–property relationships via modular modifications.^10,11,9^ BTAs can self-assemble into helical, one-dimensional supramolecular polymers via 3-fold hydrogen bonding and have been utilized in fundamental studies and applications^12^ including catalysis,^13^ polymer reinforcement,^14^ and vaccine delivery.^15^ Water-soluble versions have been shown to form long structurally complex fibers, approximately tenths of microns in length, and 5 nm in diameter.^16−18^ At higher weight percentages (2–10 wt %) these water-soluble BTAs can also form hydrogels.^10^ Additionally, the BTA core offers the ability to connect different side-arms and different BTA monomers (with various functionality) can be mixed to rapidly create libraries of multicomponent materials with tunable fibril structure, dynamics, and mechanical properteis.^10,18,5,19,17^ This modularity, fibril structure, and potential for multifunctionality make BTAs ideal candidates for biomaterials,^11^ especially toward 3D cell culture and tissue engineering applications.

**Scheme 1:**
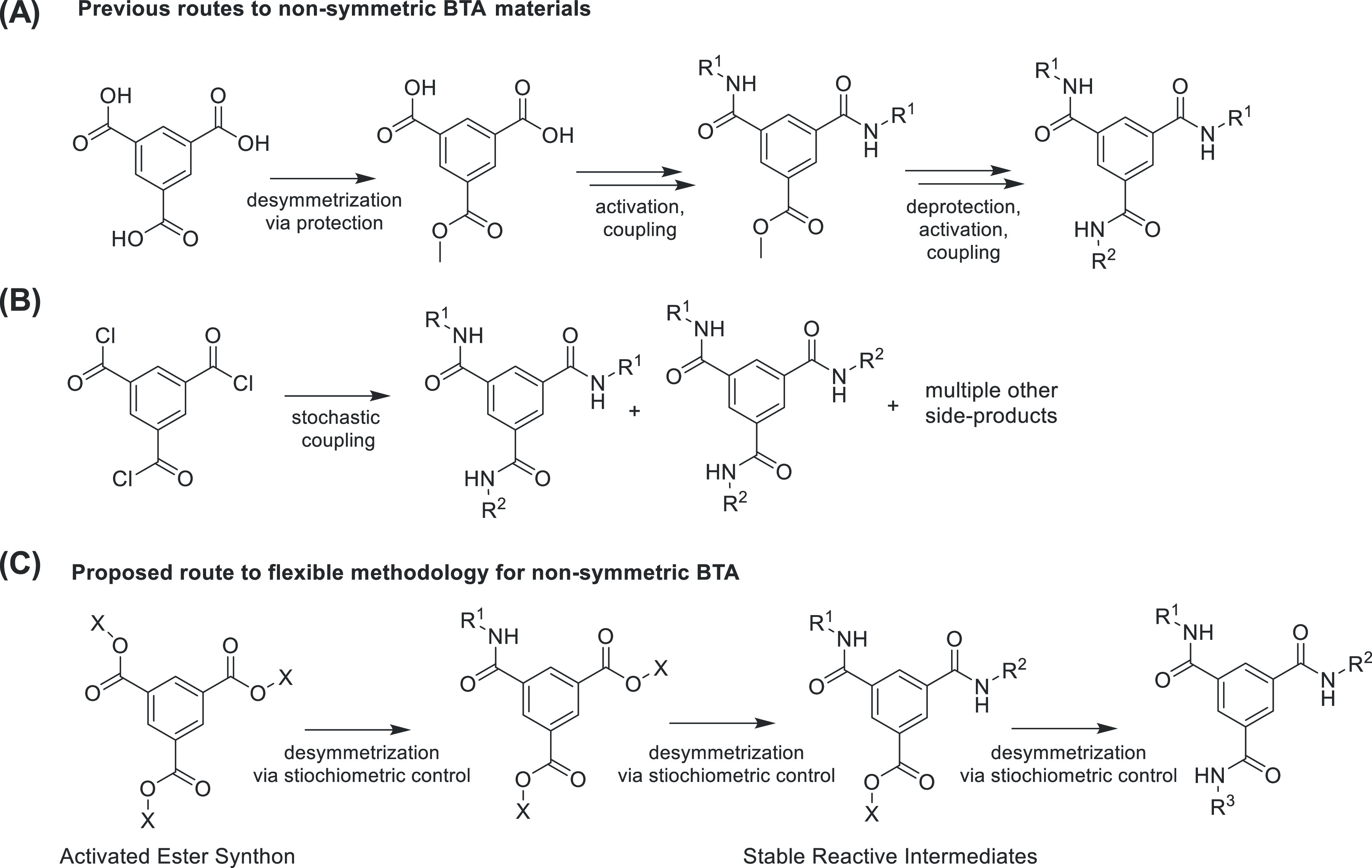
Synthesis of Non-symmetric BTAs Are Critical for Creating Functionally Diverse Supramolecular Polymers Existing routes include the following: (A) desymmetrization via protection/deprotection and functional group conversion, (B) stochastic coupling of on symmetric molecule benzene-1,3,5-tricarbonyl trichloride (BTCl) followed by extensive purification. (C) A route based on activated ester step-wise coupling with stable intermediates is an attractive synthetic route with greater speed and step economy. For example, the activated ester route developed in this article allowed creation of an ABC tri-functional BTA in less steps (4 steps from commercially available material) and has reduced the total synthetic procedure from weeks to days.

In moving toward BTA based materials (especially biomaterials), modular, scalable, flexible, and facile synthetic methods are necessary. For example, most BTAs are *C*3 symmetric, due to ease of synthesis and the symmetry of the core motif; however, non-*C*3-symmetric derivatives offer potential for increased complexity and control over the supramolecular assembly. Desymmetrization, a process to create a nonsymmetrical molecule starting from a symmetrical core, is a potential strategy to create multifunctional BTA supramolecular materials. Previous approaches toward the synthesis of multifunctional/multiarm BTA derivatives involve multiple steps and protection/deprotection strategies.^10,20,18,21−23^ These previous synthetic approaches have enabled the creation of multifunctional BTAs; however, long linear procedures (at least seven steps) and harsh deprotections limit the approach and speed (shown in Scheme 1). The challenge still remains to devise a strategy that provides the freedom to create multiarm and multifunctional BTA monomers in good yield, under mild conditions, and with a reduced number of steps.

Several strategies in the literature exist for desymmetrization of a symmetric core (cyanuric chloride,^24−26^ benzene trifurnanone,^27,28^ and polyphenylene dendrimers^29^). With the aim to form an amide bond during the desymmetrization, we chose to use an activated ester approach, using stable benzene activated triesters which are synthetically accessible and selective to aminolysis. Activated esters for amide bond formation are widely used in polymer and small molecule modifications,^30−32^ while the electronic coupling across the aromatic ring gave us the potential for kinetic activation/deactivation of the synthon upon reacting.

We aimed to create a simple, yet powerful, desymmetrization strategy to create multifunctional BTAs and polymeric supramolecular hydrogelators. We envisioned creating a small library of activated benzene-1,3,5-tricarboxyester (BTE) synthons to find a BTE molecule that can (1) shorten the route to desymmetrized BTAs, (2) produce BTA derivatives with multiple functionalities via aminolysis, (3) be selective toward controllable aminolysis, and (4) be stable under common laboratory conditions. Furthermore, we aimed to expand this desymmetrization strategy to create functional macromolecular architectures to be used as hydrogels. Taken together, we hypothesized that a new desymmetrization strategy would allow us to shorten the route for the synthesis of desymmetrized BTAs, allowing creation of multifunctionality and macromolecular BTA hydrogels opening up further exploration of BTA based biomaterials.

## Results and Discussion

### Activated Benzene-1,3,5-tricarboxyester (BTE) Library Synthesis

In attempting to create BTAs of lower symmetry, the most convenient approach would be desymmetrization of a commercial starting material. Benzene-1,3,5-tricarbonyl trichloride (BTCl) would be an ideal candidate, though the acid chloride functionality would not be envisioned to be stable to purification. Nevertheless, we attempted to react BTCl with 1 equiv of hexylamine to investigate its suitability as a synthon (Figure S1 in Supporting Information). In the absence of side products, the reaction would result in three product molecules (monosubstituted, disubstituted, and trisubstituted) and leftover starting material (upon workup it is expected that the acid chlorides would hydrolyze into acids). Upon ^1^H NMR analysis of the reaction mixture, numerous peaks appeared in the aromatic region (Figure S1B in Supporting Information, 8–10 ppm) and we could not easily obtain useful information on the constituency of the complex mixture formed during the reaction. This result was further supported by a long continuous streak on TLC without distinct spots in the reaction mixture (Figure S1C and D). The reaction was run at different temperatures (20, 4, and −75 °C) to see if the temperature could clean up the product profile; however, all attempts resulted in similar peaks on ^1^H NMR spectrum. Testing this reaction confirmed our suspicion that desymmetrization via an activated ester approach was the best way forward.

**Scheme 2:**
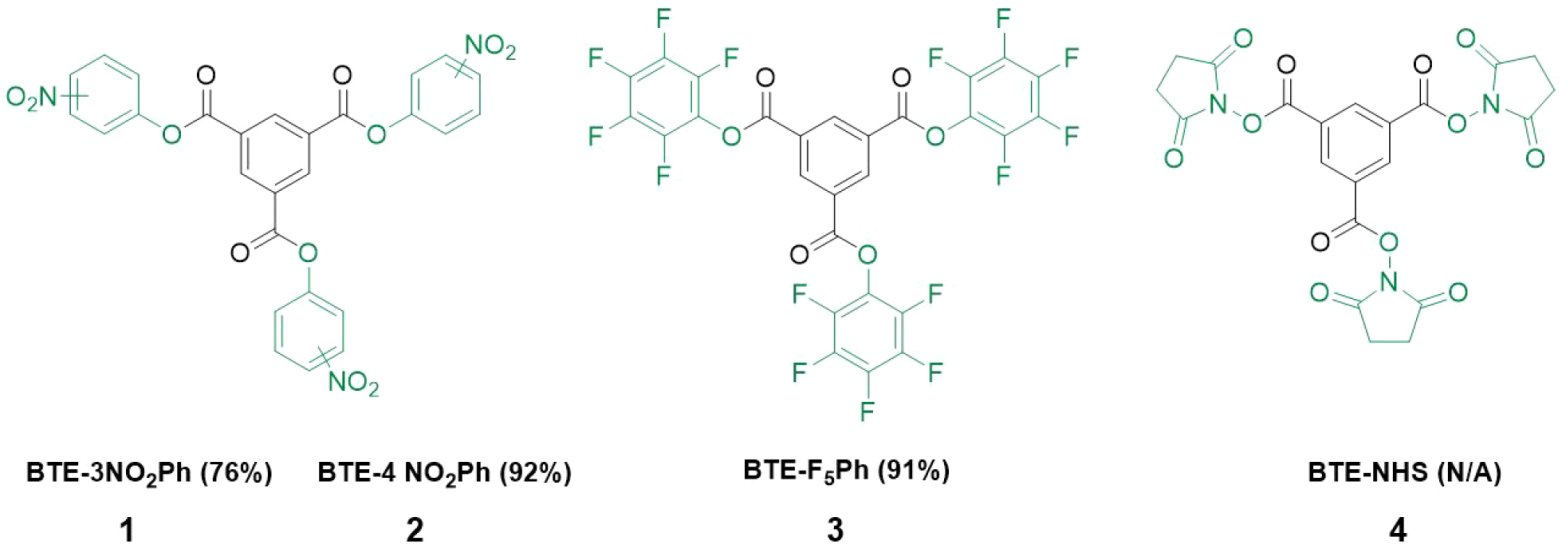
Library of Activated Benzene-1,3,5-tricarboxyesters (BTEs) Synthesized in This Study Isolated % yields are reported in parentheses.

In order to find an alternative to BTCl that can facilitate desymmetrization via aminolysis, the formation of activated esters including aromatic carboxylic esters and thioesters are appealing approaches^33^ and have remained unexplored to create BTA derivatives. Aromatic carboxylate esters were determined to be a better choice since they are less susceptible to hydrolysis and more stable compared to thioesters.^34^ Commonly employed phenols and *N*-hydroxysuccinimide, which are commercially available, were chosen for creating a library of activated benzene-1,3,5-tricarbonyl triester derivatives (BTEs) (**1**–**4**, Scheme 2). Phenols with differences in p*K*_a_ value were chosen with a p*K*_a_ of 8.36,^35^ 7.15,^36^ 5.4^37^ and 6.0^38,39^ for 3-nitrophenol (3NO_2_Ph), 4-nitrophenol (4NO_2_Ph), 2,3,4,5,6-pentafluorophenol (F_5_Ph), and *N*-hydroxy succinimide, respectively.

We started our investigation with the synthesis of nitrophenol activated esters from BTCl (Scheme 2). Both the 3-NO_2_Ph and 4-NO_2_Ph show limited solubility in many solvents (Table S1), yet tetrahydrofuran (THF) was found to be a suitable solvent facilitating reaction completion in <6 h. After optimization of purification (Table S2), **1** could be obtained in good yield (76%, Figures S2 and S3), and **2** could be obtained in excellent yield (92%, Figures S4 and S5) by recrystallization. Next, the library was expanded, and we synthesized **3** (Scheme 2) by coupling F_5_Ph to BTCl. Due to the high solubility of F_5_Ph, this reaction could be easily run in DCM in under 4 h. The reaction was clean, and TLC showed only two spots (*R*_f_ ≈ 0 and 0.9). The reaction mixture could be easily passed through a filter to remove the DIPEA salt and then through a bed of silica to yield **3** in 91% yield (Figures S6 and S7; for alternative workups, see Table S3).

Attempting the synthesis of **4** proved problematic. NHS offered limited solubility, aside from THF and DMF (shown in Table S4). Running the reaction in DMF produced only nonsymmetrical derivatives (based on ^1^H NMR spectrum Figure S8), while running the reaction in THF produced the symmetrical target compound **4**. TLC analysis of the reaction showed two spots (Figure S9); however, **4** was not able to be fully isolated from free NHS by flash chromatography under several mobile and solid phase conditions (Figure S10). Furthermore, pure **4** was not obtained via crystallization, and during numerous workup attempts, the ^1^H NMR evolved extra peaks suggestive of degradation. Importantly, our experiments show that production and isolation of **4** is not straightforward and appears to be very sensitive to degradation during handling.

### Desymmetrization of activated BTE synthons

After a mostly successful BTE library synthesis, we moved to investigate the desymmetrization potential of the symmetrical BTEs. Via stoichiometric control, we aimed to maximize the % yield of monosubstituted and disubstituted derivatives. For reference, previous statistical simulations showed a maximum of 37% monosubstituted derivative using 1 equiv of the nucleophile^27^ for a triply reactive system. Using hexylamine as a model nucleophile, we set out to create a monosubstituted derivative (using 1 equiv (per BTE synthon)). DMF was found to be the best solvent for **1** (although not fully soluble, Table S5), and during the reaction dissolution occurred (Figure S11).

**Scheme 3:**
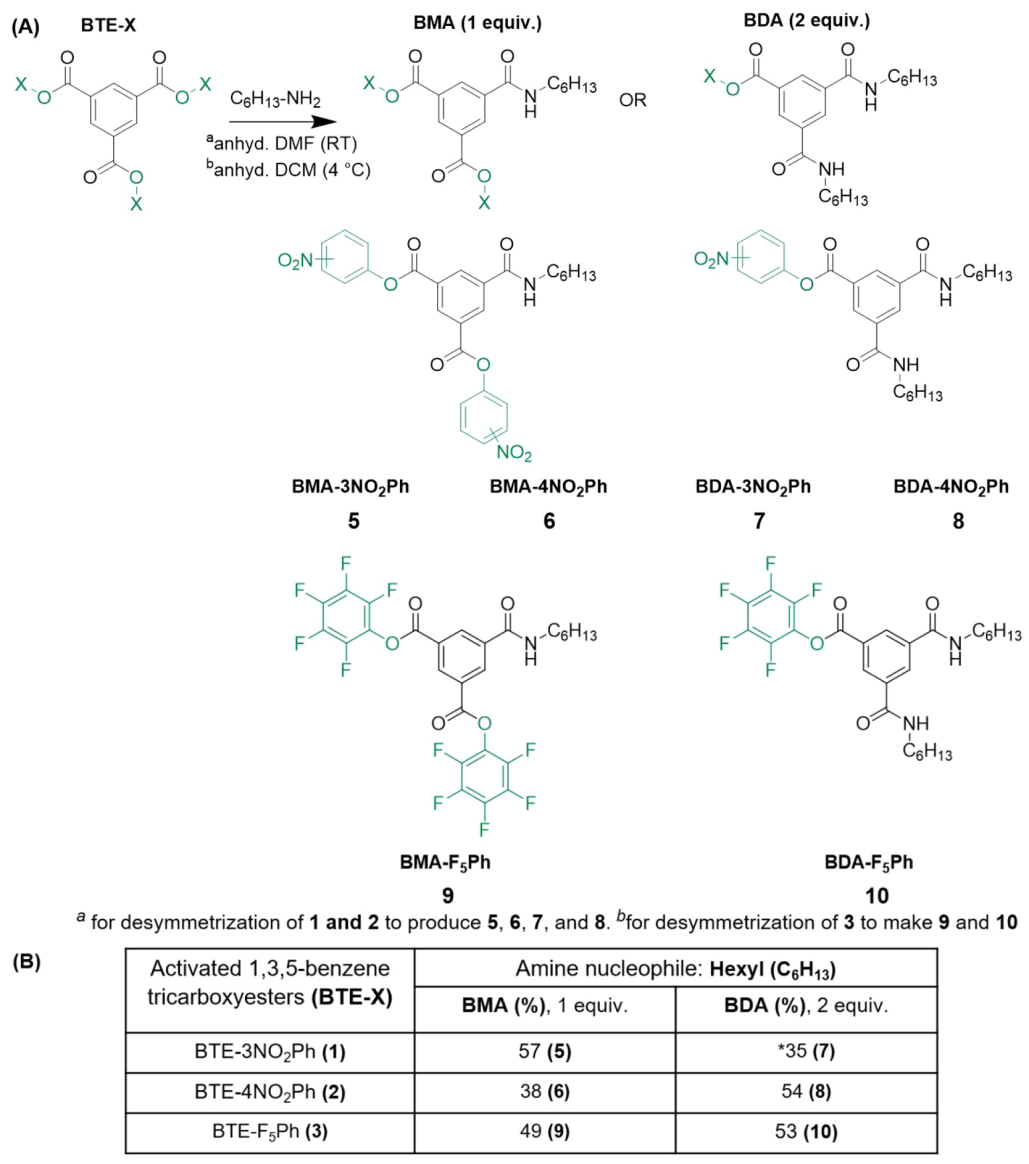
Investigating Desymmetrization of Activated BTEs via Controlled Aminolysis Using Hexylamine (A) All BTEs could be successfully desymmetrized. (B) Reaction yields are reported in the table and calculated from ^1^H NMR integration analysis. Molecules **7**–**10** could be obtained pure; however, molecules **5** and **6** were not stable to purification. *Difficulty with stoichiometry control attributed to instability of **1**.

In the absence of any side products, desymmetrization of molecule **1** (Scheme 3 and Figure S11 and S12) would result in five molecules in the reaction mixture (monosubstituted, disubstituted, trisubstituted, free phenol, and remaining **1**). TLC of the reaction mixture of **1** showed three spots suggesting that some products were not formed or only formed in small amounts (Figure S11C).

Reaction mixtures resulting from the substitution of molecule **1** were partially soluble in DCM, and the products could be isolated via column chromatography, though **7** and the trisubstituted BTA could not be fully resolved. ^1^H NMR analysis (chemical shift, peak splitting pattern and integration analysis of peaks) was used to identify the compounds (Figures S13 and S14). Most importantly, this separation allowed us to identify the complex aromatic peak splitting found in the reaction mixture and assign peak patterns to the mono-, di-, and trisubstituted derivatives (doublet and triplet at 8.94 and 8.91 ppm for **5**, doublet and triplet at 8.71 and 8.65 ppm for **7**, and singlet at 8.34 ppm for the trisubstituted BTA).

With the knowledge of proton chemical shift and peak splitting pattern of products, we were able to analyze the crude reaction mixtures (Figure S12 for 1 equiv) and quickly determine the relative amount of the products in the reaction mixture. Molecule **1** produced a 57% yield of **5** (monosubstituted) when treated with 1 equiv of nucleophile. The desymmetrization of **2** behaved similarly (Figures S15 and S16), and after separation and characterization, the crude reaction mixture produced a 38% yield of **6** (monosubstituted derivative, Figure S17). Interestingly, **1** produced the monosubstituted product significantly higher than statistically calculated, while **2** produced almost equal to statistically predicted. When the same reaction was run with 2 equiv of hexylamine, **1** produced **7** (disubstituted) in 35% yield (Figure S14) and **2** produced **8** in 54% yield (Figure S18). Compound **2** produced the disubstituted derivative roughly twice the statistical prediction, which is 28% using 2 equiv of the nucleophile.^27^

With promising results, we then turned to desymmetrization of the pentafluorophenol synthon, **3**. After running the reaction with 1 equiv of hexylamine in DCM, the crude reaction mixture showed five spots on TLC (Figure S19), which were isolatable via column chromatography. After ^1^H NMR analysis, we were able to determine the ratio of substituted derivatives in the crude reaction mixture (a similar pattern of doublet/triplets and singlets was observed as in the desymmetrization of **1**). Peak integration from the ^1^H NMR spectrum of the crude reaction mixtures showed that **3** produced 49% of **9** (Figures S20 and S21) and 53% of **10** (Figures S22 and S23) using 1 and 2 equiv of hexylamine, respectively. Both desymmetrization reactions yielded higher than statistical yields, and the products were able to be readily isolated via column chromatography.

When developing a reactive synthon for desymmetrization, stability and scalability are also important factors to consider. Both **1** and **2** were stable in a desiccator when stored for a year; however, upon handling in the lab over 2–3 months both molecules started to hydrolyze (^1^H NMR). We did observe that **1** was more stable than **2**. In comparison, **3** showed excellent stability in the lab; it was found to be stable for more than two years, even after open handling in a humid environment (The Netherlands). Purification of **1** and **2** required a large volume of solvents owing to limited solubility (100s of mL for 10s of mg), while **3** offered a short one-step workup with good solubility.

Due to its ease of synthesis, stability, desymmetrization, and purification, **3** was determined to be the best candidate to work with moving forward. We found **3** was stable over years under an inert atmosphere, was stable in the humid environment of the lab, was purified in a short one-step workup and easily scaled to gram scale, showed good solubility in low boiling point solvent, and displayed simple and straightforward NMR analysis. Furthermore, the stability of the F_5_Ph esters on **3** also offers easy separation of desymmetrized intermediates using flash column chromatography.

### Desymmetrization of BTE-F_5_Ph (**3**)

**Scheme 4:**
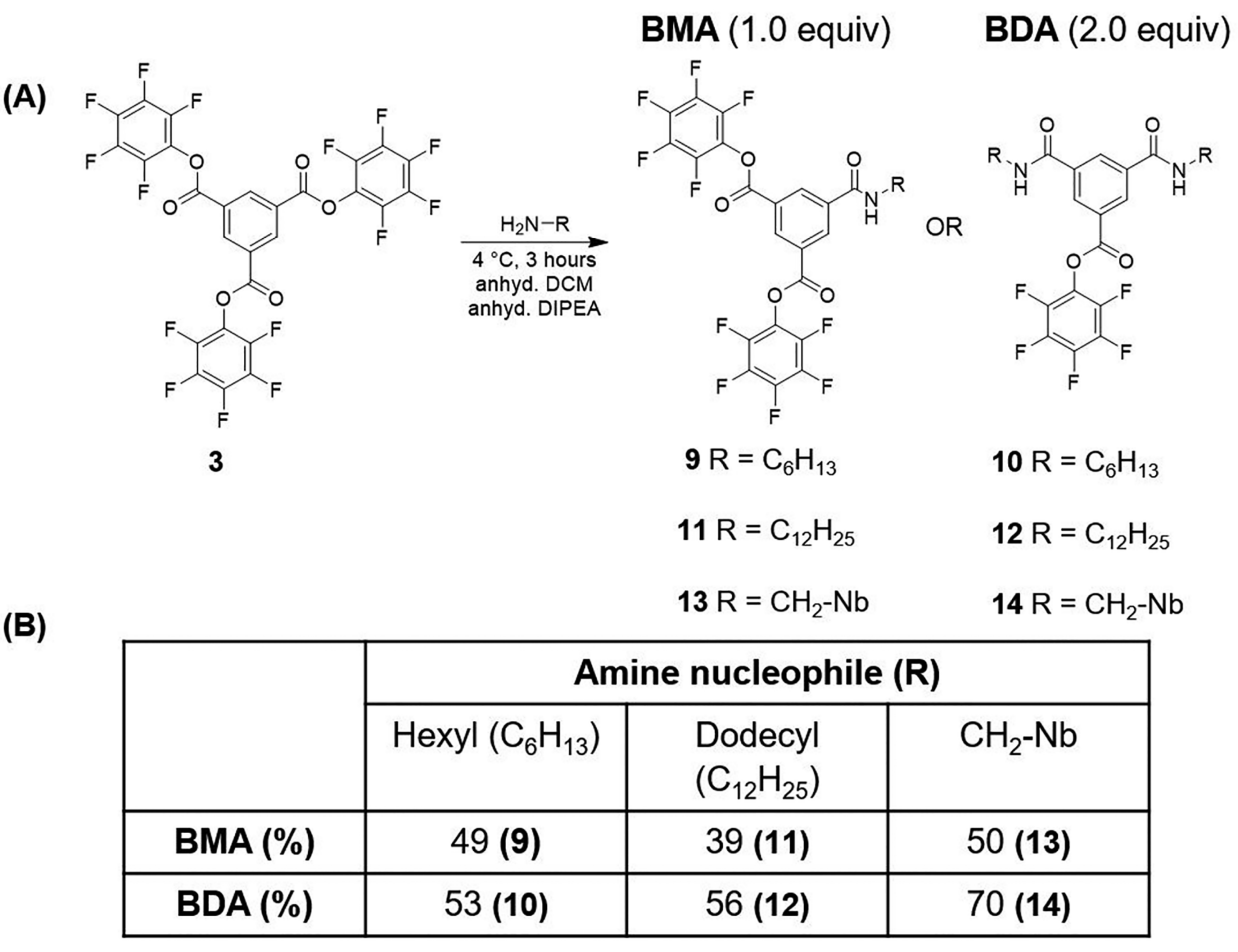
Desymmetrization of Synthon **3** (A) Desymmetrization was tolerant to aliphatic amines such as hexylamine (C_6_H_13_), dodecylamine (C_12_H_25_), and methyl norbornene (CH_2_–Nb). (B) Reported % yield of BMA and BDA molecules formed during a reaction, derived from ^1^H NMR integration analysis.

In order to investigate if temperature affected the product outcome in the desymmetrization of **3**, we attempted desymmetrization at different temperatures. Using 1 mole equiv of hexylamine, **9** was made in 50% yield at 4 °C (Scheme 4 and Figures S20 and S21), and this yield remained 50% when the reaction was run at −78 °C (Table S6). Producing the disubstituted derivative **10** (Scheme 4 and Figures S22 and 23) from 2 equiv of amine resulted in a 53% and 65% yield at 4 °C and −78 °C, respectively, showing a small temperature influence on the second aminolysis. In these test reactions, we observed only a small (5–10%) decrease in the yield after separation using silica gel flash column, resulting in isolated yields of 40% (**9**) and 48% (**10**).

Next, we wanted to test the applicability of the reaction methodology to different amine-based nucleophiles/side-arms, and we analyzed a few different amines. Dodecylamine resulted in a maximum 39% yield of **11** (monosubstituted derivative, 1 equiv amine, Scheme 4 and Figures S24 and S25) and a 56% yield of **12** (disubstituted derivative, 2 equiv amine, Scheme 4 and Figures S26 and S27) at 4 °C. There was little change in produce profile when running at −78 °C, and the isolated yields again showed the stability of the activated ester to handling and purification (e.g., 49% isolated yield for **12**). The desymmetrized synthons (**9**, **10**, **11**, and **12**) were found to be stable over months under an inert and dry atmosphere in a desiccator at room temperature, indicating the potential for storage and resumption of synthetic pathways toward multifunctional BTAs.

After successful desymmetrization using simple hydrophobic side-arms, we next explored more functional side-arms. Monosubstituted and disubstituted derivatives with 5-Norbornene-2-methylamine (5Nb-2MA, Scheme 4 and Figure S28) were targeted using 1 and 2 mole equiv to produce **13** and **14**, respectively. Conveniently, **13** (Figures S29 and S30) and **14** (Figures S31 and S32) were produced in 50% and 70% yield (via ^1^H NMR integration). Going further, we explored if an amine nucleophile would show selectivity over a hydroxyl nucleophile when in competition for the activated ester. To investigate this, **3** was desymmetrized using 1 equiv of 6-amino-1-hexanol (Figure S33), and we find that the amine selectively acted as a nucleophile over the hydroxyl. ^1^H NMR analysis showed that 40% of the molecules were monosubstituted, and no traces of the hydroxyl substituted core were observed.

### Molecular Origins of Selectivity

With a robust and scalable synthon in hand, we also wanted to briefly investigate the origins of slightly higher than statistical yields for the desymmetrization of **3** into (for example) **9**, and **10**. When looking at molecular descriptors like Hammet substituents, we can see that the ester and the amide have similar electron-withdrawing ability (σ_m_ = 0.37 for COOMe and 0.35 for CONHMe);^40^ however, one could imagine that an activated pentafluorphenyl ester would have a stronger electron-withdrawing effect, though literature values could not be readily found. This would then set up a system in which each subsequent amide bond formation could affect the ring electronics and thereby alter the reactivity of the remaining activated esters. Reduced reactivity has been observed in literature during aminolysis of substituted phenyl acetates and has been attributed to an increase in electron density at central benzene ring owing to the introduction of less electron-withdrawing substituent upon aminolysis.^41,42^ Kinetic deactivation during sequential aminolysis of benzotrifuranone has also been linked to reduced reactivity upon successive aminolysis and associated with the synergism of electronic effect and ring strain.^28,43^

In order to further test this hypothesis, we turned to DFT calculations (RB3LYP/6-311G*) on structures **3**, **9a**, and **10a** to determine the changes in reactivity descriptors upon sequential aminolysis. Of note, molecular structures **9a** and **10a** were related to structures **9** and **10**, but with a truncated methyl amide. Several molecular indices are tabulated in Table S7. Immediately apparent in the energy minimized structures (see Supporting Information) was the planar arrangement of the esters with respect to the central aromatic ring, yet not the pentafluoro ring, suggesting a preference for orbital overlap with the central ring. Also, immediately we saw that the C=O length increases upon each sequential aminolysis event (about 0.001 Å, per aminolysis). This suggested a weakening of the carbonyl and could suggest a decreased reactivity. Digging further, we saw several reactivity descriptors for ester reactivity suggested decreased reactivity across the series. The occupancy of the π* orbital (from NBO analysis) increased, the global electrophilicity index (ω) decreased, and the energy of the LUMO increased. These frontier molecular orbital indicators all suggest that attack of a similar nucleophile should become less favored down the series. Surprisingly, the indicators of charge at the carbonyl carbon all suggested decreased positive character (V_c_, NBO charge, δ ^13^C), which could suggest decreased reactivity, but these exact trends between orbital analysis and atomic charge have been documented in the highly selective benzotrilactones previously explored.^28,43^ In summary, we see strong evidence for electronic coupling across this ring system, and orbital reactivity indices indicate a decreased reactivity with each subsequent aminolysis step.

### Multifunctional BTA Derivatives Synthesis

**Scheme 5:**
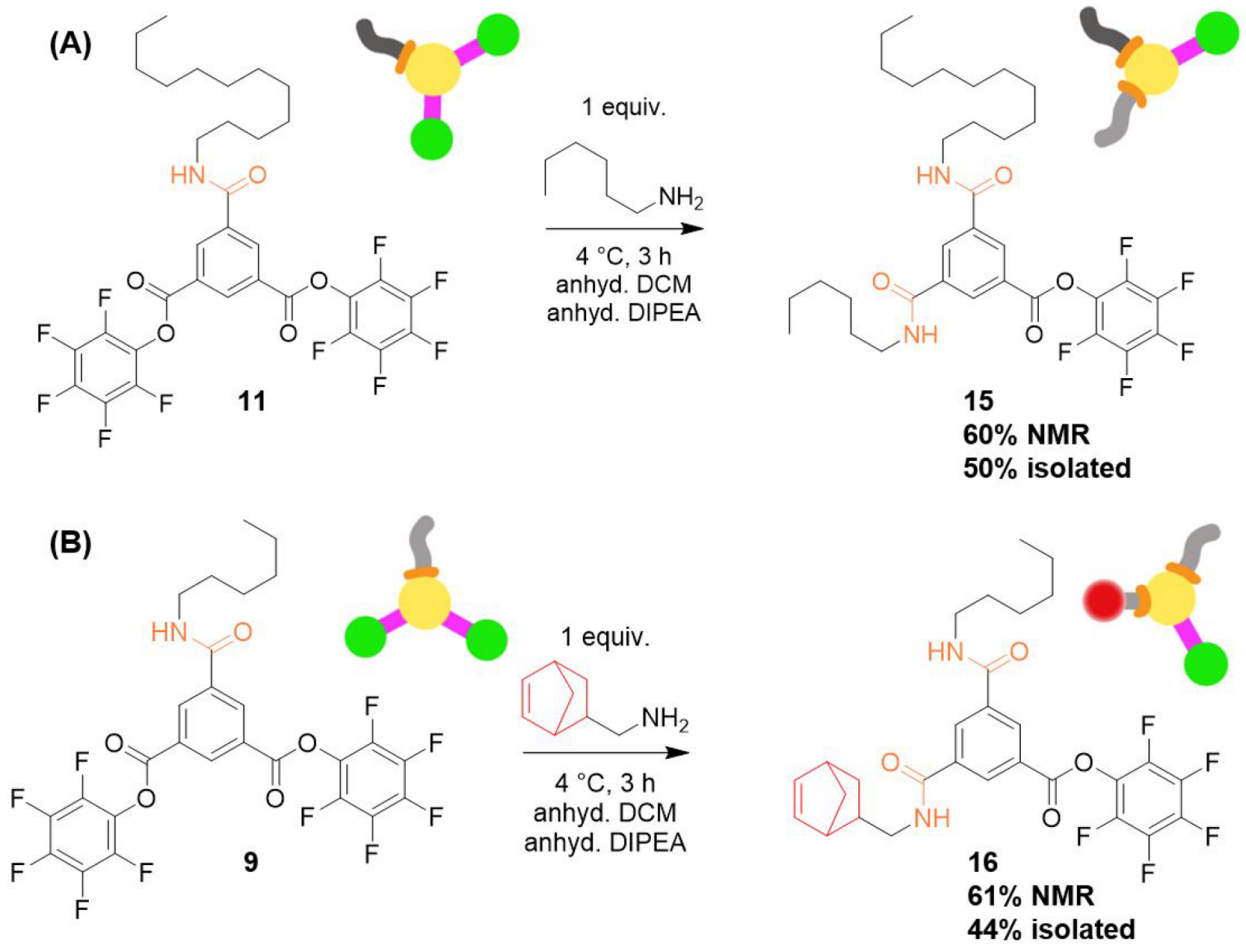
(A) Molecule **15** with Dodecyl and Hexyl Aliphatic Side-Arms Was Synthesized by Desymmetrizing Molecule **11** Using Hexylamine; (B) Molecule **16** Was Synthesized by Desymmetrizing Molecule **9** Using 5-Norbornene-2-methylamine Molecule **15** was obtained in 60% yield by ^1^H NMR integration analysis. Molecule **16** was obtained in 61% yield by ^1^H NMR integration analysis. Norbornene is a light active functionality and offers potential to be employed later for attaching biological molecules.

With the confidence that we could install different side-arms and functionalities on BTA using this activated ester methodology, we moved to create BTA derivatives with different side-arms. Monosubstituted **11** was utilized and desymmetrized further, producing **15** with one dodecyl and one hexyl side arm (Scheme 5 and Figures S34–S36). This reaction resulted in a 60% yield based on ^1^H NMR analysis and a 50% isolated yield. Next, we created a molecule with one hydrophobic side arm and one reactive functionality (norbornene), which could later be utilized for thiol–ene, norbornene–tetrazine, or ROMP polymerization. From **9**, **16** was made (Scheme 5 and Figures S37 and S38) in 61% yield by ^1^H NMR and 44% yield isolated. It is important to note that in both of these reaction pathways the starting monosubstituted **9** and **11** were recovered around 10–15% and can be utilized in future reactions; thus, the isolated yield based on recovered starting material approached 60%.

**Scheme 6:**
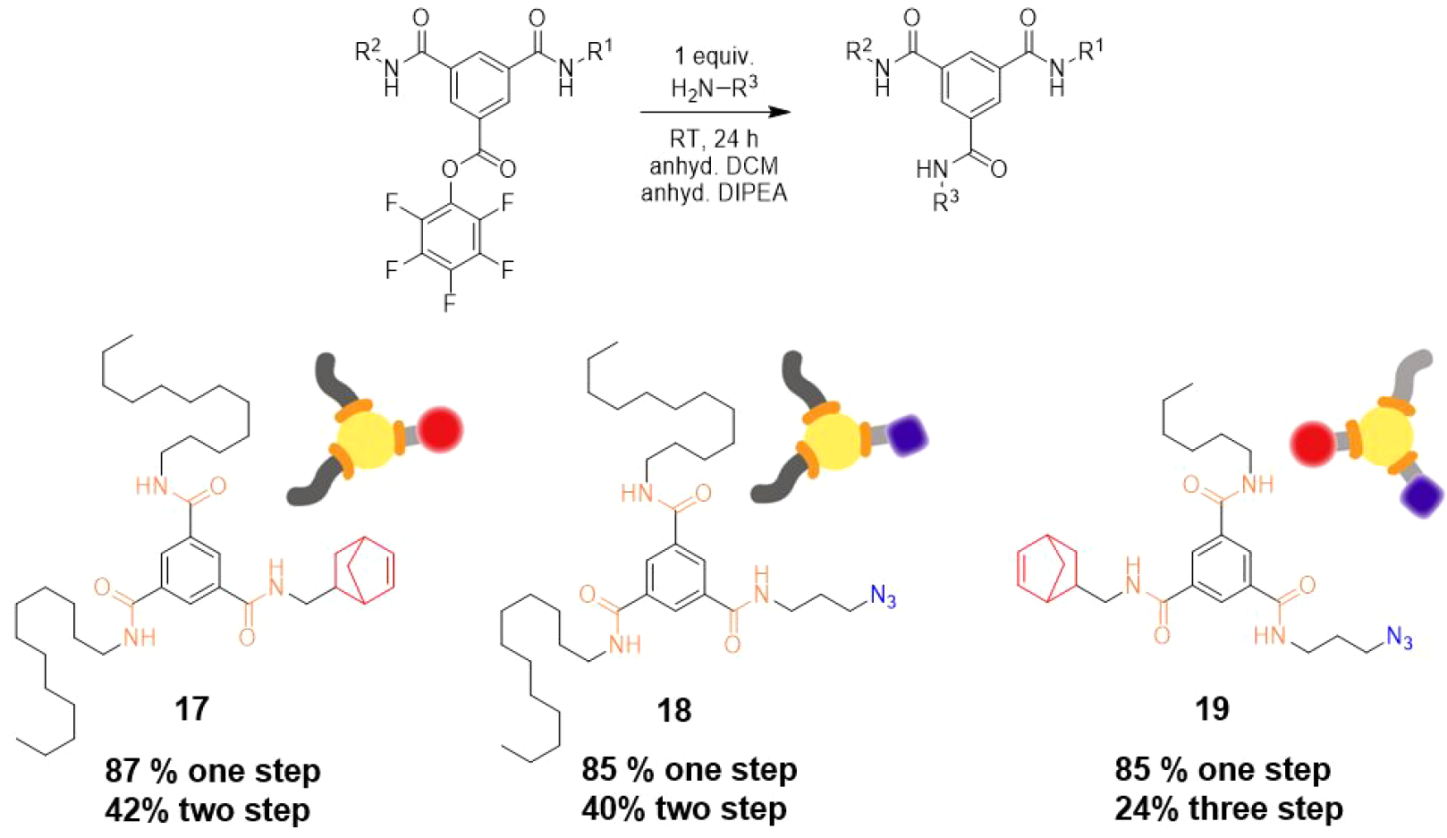
Final Step toward the Production of Non-symmetric BTAs Partially desymmetrized BTAs **17** and **18** were synthesized using **12** and attaching methyl norbornene or propyl azide. Fully desymmetrized, ABC type BTA **19** was synthesized by coupling propyl azide to **16**. Such multifunctional azide and norbornene BTAs can be used to functionalize supramolecular fibers or individual BTAs.

Knowing that we can create a multifunctional disubstituted derivative, next we wanted to create trisubstituted BTA derivatives. We utilized **12** with two dodecyl side-arms and reacted the last activated ester to either 5Nb-2MA or 3-azido-1-propanamine to create functional BTAs **17** and **18** (Scheme 6) with orthogonal reactive handles. After an overnight (16–20 h) reaction in DCM, ^1^H NMR analysis showed 100% conversion of **12** to both **17** (Figures S39 and S40) and **18** (Figures S41 and S42). The isolated yield (from **12**) for **17** and **18** was 87% and 85%, respectively, while the linear two step yield (starting from **3**) was 42% and 40%, respectively. This shows that the high fidelity desymmetrization can be expanded to functional handles, and the potential can be utilized to attach biological molecules as well as probes and effect postassembly modifications of resultant supramolecular polymers.

Finally, we aimed to create a fully desymmetrized ABC type BTA in order to test the full desymmetrization efficiency. In three steps, we synthesized BTA **19** (starting from **16**) with a hydrophobic side arm (hexyl), norbornene side arm, and an azide side arm (Scheme 6) as orthogonal functionalities. After separation, **19** was obtained in 85% isolated yield (Figures S43 and S44). Starting from symmetrical **3** to create **19**, the full linear yield of the reaction was 31% by ^1^H NMR and 24% by mass. This approach doubles the yield (12%) afforded by already existing desymmetrization routes to create an ABC BTA.^18,21^ This represents the first linear and general approach toward fully desymmetrized BTAs and is envisioned to work with a wide variety of amine side-arms due to the high fidelity and mild reaction conditions.

### Molecular Design and Synthesis of Polymeric BTA (BTA-PEG20K-BTA)

Next, we moved to explore if the developed methodology could be employed for the rapid and facile creation of polymeric supramolecular macromolecules in addition to the small molecules presented above. Toward this aim, we wanted to employ our new methodology to create a small set of telechelic BTA-PEG-BTA polymers to be used as potential hydrogelators and 3D environments for cell culture. Previous work showed that a hydrophobic spacer is required to protect the BTA amides in an aqueous environment, and these BTAs undergo self-assembly via hydrogen bonding and hydrophobic interactions to form long fibrils.^16,20^ Previous studies on telechelic BTAs have also shown that a minimum of an eight carbon hydrophobic spacer was needed for stable hydrogel formation;^10^ however, the ability to vary the outer side arm on these hydrogelator architectures had been limited by the previous methodology.

**Scheme 7:**
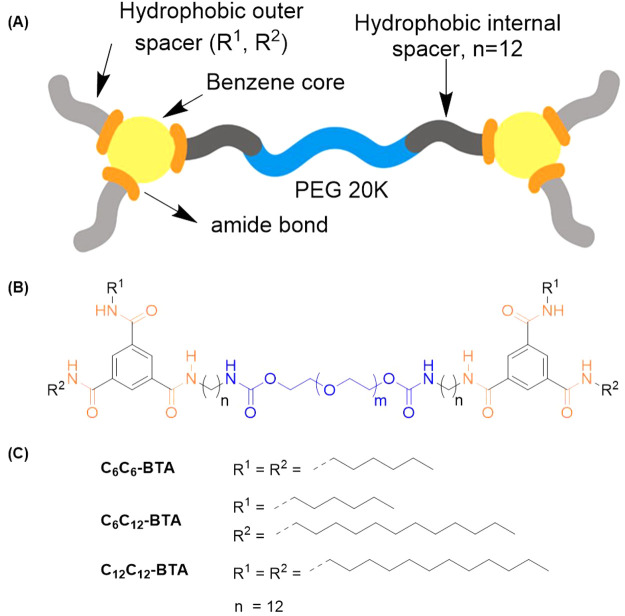
(A) Schematic Representation of Macromolecular Hydrogelators Targeted in This Study; (B) Chemical Structure of BTA Hydrogelator Made by Connecting Two Small Molecule BTAs with Bisaminododecane PEG20K; and (C) Hydrophobic Side-Arms Employed to Create Hydrogelators **C**_**6**_**C**_**6**_, **C**_**6**_**C**_**12**_, and **C**_**12**_**C**_**12**_

In our design, we chose dodecyl as a hydrophobic spacer between BTA and PEG20K and varied the outer side-arms on the BTA (Scheme 7). Amine end-functionalized PEG with dodecyl as an internal spacer (bisaminododecane PEG20K, Figures S45 and S46) was created by conjugating dodecyl diamine to PEG20K diol using carbonyldiimidazole (CDI) chemistry. A small set of BTA macromolecules with C_6_ side arms (**C**_**6**_**C**_**6**_), mixed C_6_ and C_12_ side arms (**C**_**6**_**C**_**12**_), and C_12_ side arms (**C**_**12**_**C**_**12**_) was generated by coupling bisaminododecane PEG20K to **10**, **15**, and **12**. It is important to note that this newly developed methodology not only allowed the rapid variation of outer side-arms on the BTA but also allowed the creation of macromolecular BTA with mixed outer side-arms containing boty hexyl (C_6_) and dodecyl (C_12_).

All telechelic architectures were obtained with more than 95% yield, showing the high fidelity of the final pentafluorophenol ester for conjugation to macromolecules. ^1^H NMR showed that all polymers are pure and have a high degree of functionalization 81%, 67%, and 78% for hydrogelator **C**_**6**_**C**_**6**_, **C**_**6**_**C**_**12**_, and **C**_**12**_**C**_**12**_, respectively (Figures S47–S49). GPC analysis confirmed that all macromolecules have a weight-average molecular weight (*M*_w_) of around 23 kg/mol with a dispersity index (Đ) of 1.2 (Table S7). With this new methodology, we were able to make new telechelic BTA architectures on a multigram scale under 2 weeks, which indicates the rapid large scale capability of the designed methodology.

### Self-Assembly Studies

With the small set of telechelic BTA supramolecular macromolecules in hand, we wanted to explore the self-assembly of these materials and their potential use as hydrogelators. Previous studies have shown that successful BTA assembly in water requires a hydrophobic pocket formation to facilitate amide stacking and stable aggregate formation.^16^ Consequently, we initially utilized a Nile Red assay to investigate the hydrophobic pocket formation upon assembly. In pure water, Nile Red shows very low quantum yield, and the quantum yield increases in more apolar environments.^44^ Self-assembly of BTAs have been shown to increase the fluorescence intensity of Nile Red in solution, owing to the hydrophobic pocket formation.^16,5^ In dilute solution, we observed an increase in fluorescence with increasing hydrophobic length on the exterior of the BTA (Figure S50), suggesting an increase in the size and volume of the hydrophobic pocket, as would be expected. This appeared regular, as the fluorescence intensity increased twice when the hydrophobic length was doubled on a single BTA unit, from **C**_**6**_**C**_**6**_ to **C**_**12**_**C**_**12**_. Furthermore, the fluorescence intensity increased with increasing concentration for all hydrogelators (1 to 2 to 5 mg/mL) indicating BTA aggregation, and no major nonlinearity was seen in the volume of the hydrophobic pocket (Figure S50A–C).

The maximum wavelength (λ_max_) of Nile Red emission can be used to probe the polarity of the hydrophobic pocket. For all tested concentrations 1 mg/mL (45 μM), 2 mg/mL (90 μM), and 5 mg/mL (230 μM), the maximum fluorescence intensity is near ∼621 nm (compare to PEG20K solution in water λ_max_ = 647 nm, Figure S50D–E), indicative of a nonpolar environment. Furthermore, we did not observe major changes in the λ_max_ as a function of concentration or molecular architecture, suggesting a similar polarity hydrophobic pocket across both series.^16^

**Figure 1:**
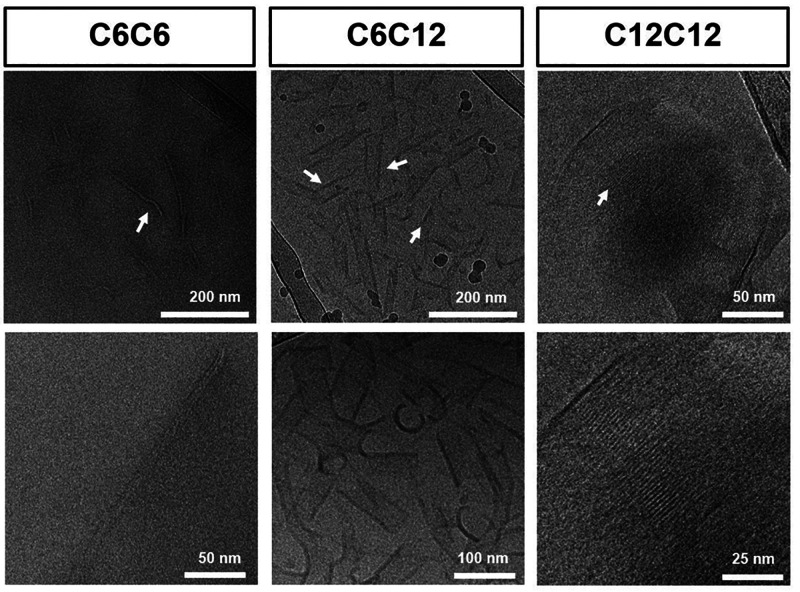
Self-assembly studies: (A) cryo-TEM images showing the fibrous self-assembly morphology for **C**_**6**_**C**_**6**_ and fibrous sheet like morphology for **C**_**6**_**C**_**12**_ and **C**_**12**_**C**_**12**_ at 10 mg/mL. BTA samples were dissolved in minimal methanol and then self-assembled in water.

With confidence from the Nile Red experiments that the BTAs formed a hydrophobic pocket via aggregation, we moved to investigate the morphology of the aggregates in water solution. Cryogenic transmission electron microscopy (cryo-TEM) was employed to investigate the BTA macromolecules structure in dilute solution at 10 mg/mL (Figure 1). Moving up the series, we observed that the fibers became more sheet-like and longer with increasing hydrophobic character on the outside of the polymer. Macromolecule **C**_**6**_**C**_**6**_ showed thin fibers between 5 and 10 nm in diameter, **C**_**6**_**C**_**12**_ showed fibrous sheets of width between 20–60 nm, and **C**_**12**_**C**_**12**_ showed fibrous sheets of width around 150 nm that consisted of nanofibers resting parallel and next to each other. All the macromolecules showed successful self-assembly in dilute solution. Furthermore, the self-assembly resulted in the formation of fibrous structures, which can start to mimic ECM fibrous morphology as seen in collagen and fibrin.

Cryo-TEM studies showed stable, clear, and observable structures at 10 mg/mL for all hydrogelators (Figure 1). We observed no structures at 1 mg/mL; at 5 mg/mL (aged sample for 30 days), a sheet-like structure was observed for **C**_**12**_**C**_**12**_, and no clear and defined structures were observed for **C**_**6**_**C**_**6**_ and **C**_**6**_**C**_**12**_ (Figure S51). Then, we dissolved the hydrogel (10% w/v) until it became a clear solution, and similarly a network of fibrous sheets was found for **C**_**12**_**C**_**12**_ while no defined structures were discovered for **C**_**6**_**C**_**6**_ and **C**_**6**_**C**_**12**_ (Figure S51). Observing no clear structure at low concentration (1 and 5 mg/mL) under cryo-TEM contrasts the Nile Red data, which shows a significant increase in fluorescence intensity and blue shift (621 nm). Then, we quickly recorded **C**_**12**_**C**_**12**_ Nile Red fluorescence at 10 mg/mL and still observed similar spectra (Figure S50C). The Nile Red data indicated that structure formation results in a similar hydrophobic pocket independent of concentration, which would suggest similar structure formation at low concentration. No identification of high aspect ratio structures at low concentration using cryo-TEM would most likely be ascribed to small spherical micelles, or nonhomogeneity in the self-assembly of the sample. These subtle differences in self-assembly remain to be elucidated in future detailed studies.

### Hydrogel Formation and Critical Gelation Concentration (CGC)

**Figure 2:**
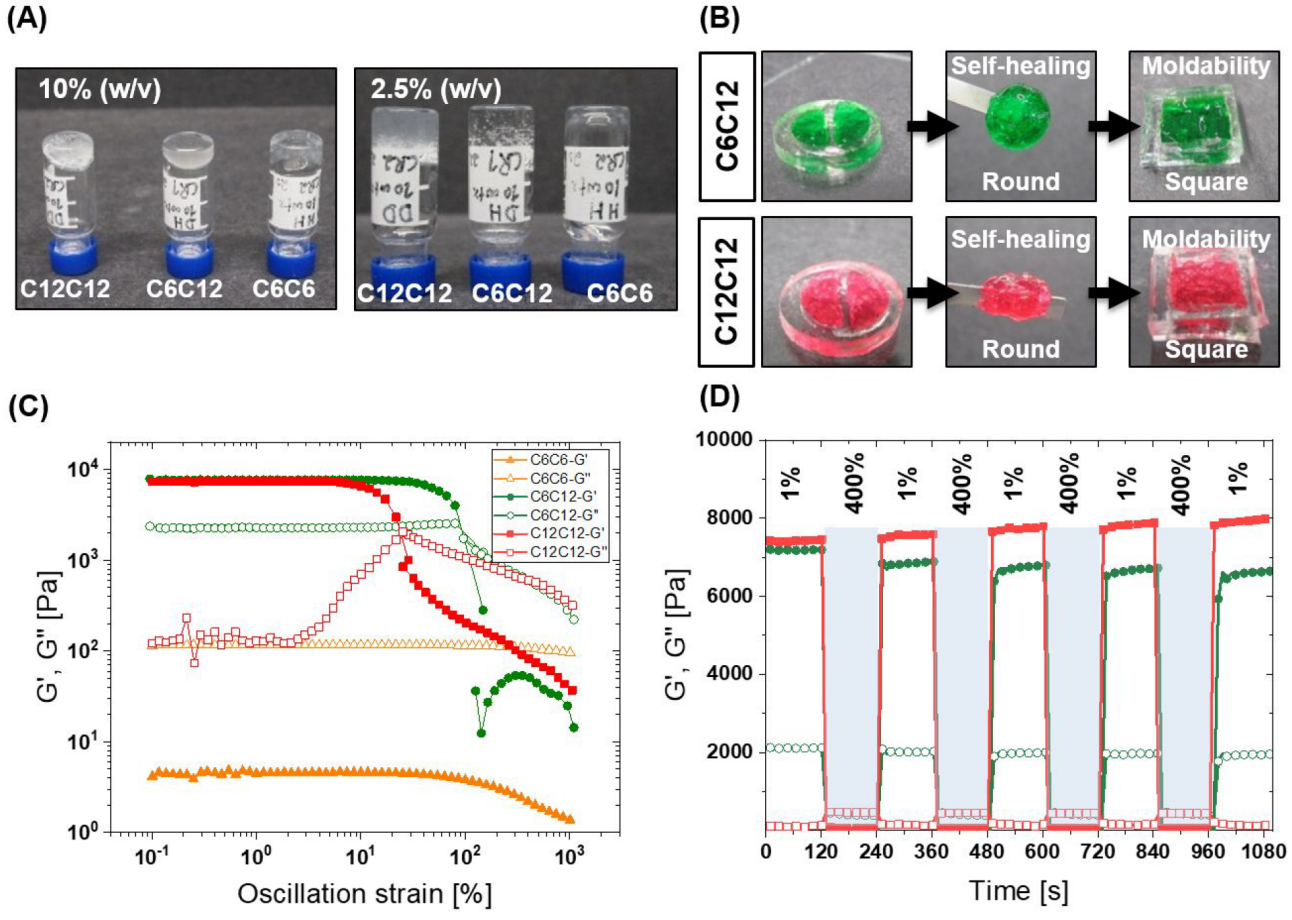
Mechanical properties of hydrogels and self-healing: (A) All BTA supramolecular macromolecules form hydrogels at 10% (w/v). Vial inversion test showed critical gelation concentration was dependent on exterior hydrophobic length. (B) Hydrogels are self-healing when two cut pieces of hydrogel are pressed against each other. Hydrogels can be remolded into different macroscopic shapes by pressing with a spatula; for example, a round hydrogel can adapt the square shape (green **C**_**6**_**C**_**12**_; red **C**_**12**_**C**_**12**_). (C) In the strain sweep **C**_**6**_**C**_**12**_ and **C**_**12**_**C**_**12**_ are observed to have the same moduli, with a greater linear viscoelastic regime for **C**_**6**_**C**_**12**_. At this frequency (1 rad/s), **C**_**6**_**C**_**6**_ was mostly liquid. (D) Hydrogels are self-healing under shear rheology. After rupture, the materials quickly recover to initial moduli in just few seconds. **C**_**6**_**C**_**12**_ is in green, and **C**_**12**_**C**_**12**_ is in red color. Rheology samples were measured at 10% (w/v).

First, we assessed the ability of the telechelic BTA supramolecular macromolecules to form a hydrogel in water. To this end, we formed 10% (w/v) hydrogels via a successive (3×) heat/cool procedure. A final heat/cool cycle on a heating plate (80 °C) with slow cooling to 20 °C was employed to facilitate controllable hydrogel formation. Next, we were interested to determine the critical gelation concentration (CGC), via a qualitative vial inversion (no flow under 30 s). We found that CGC depends on the hydrophobic length; architectures with a more hydrophobic exterior result in lower CGC (Figure 2A and Figure S52). Hydrogelator **C**_**6**_**C**_**6**_ and **C**_**12**_**C**_**12**_ showed a CGC of ∼5% and ∼2.5% (w/v). The difference in CGC indicates the importance of the hydrophobic pocket on the formation of a gel at low concentrations, and this 2-fold higher CGC is conspicuously mirrored by the 2-fold higher Nile Red intensity seen above. Taken together, we can conclude the dodecyl spacer resulted in larger hydrophobic pocket formation and stable aggregate formation, which provided more stability to hydrogels. Interestingly, the asymmetric hydrogelator **C**_**6**_**C**_**12**_ showed the lowest CGC of 1.42% (w/v); however, the exact reason for this remains unknown. The described fully desymmetrized architectures are potentially interesting for future studies.

### Mechanical Properties of Hydrogels

Next, we set out to investigate the mechanical properties of the resultant hydrogels. First, we qualitatively investigated the flow behavior of the hydrogelators with the vial inversion test. All hydrogels were made at an initial concentration of 10% (w/v) to investigate the mechanical properties. As shown in Figure S53, the hydrogelators flowed (or did not flow) on different time scales, from minutes (**C**_**6**_**C**_**6**_) to no flow after 72 h (**C**_**12**_**C**_**12**_). A significant difference in flow behavior suggests that these gels have different viscoelasticity.

In order to investigate the storage moduli and dynamic mechanical properties of these hydrogels, we turned to cone–plate rheology. A frequency sweep was performed at 1% strain, within the linear viscoelastic region. As seen in Figure S54, all hydrogelators showed nearly identical equilibrium shear storage moduli around 10,000 Pa. The similar equilibrium storage modulus of these materials would suggest a similar topology or morphology of the hydrogels, as the cross-link density remains relatively constant at a given concentration between the series. Interestingly, the viscoelastic properties of the hydrogel changed greatly as a function of the side-arms. Hydrogelator **C**_**12**_**C**_**12**_ showed a storage modulus largely independent of frequency across almost five decades, while hydrogelators **C**_**6**_**C**_**6**_ and **C**_**6**_**C**_**12**_ responded dynamically to applied shear; the storage moduli increased from low to high frequency and plateaued (Figure S54). While the similar equilibrium storage moduli indicated a similar internal structure to the hydrogel, the differences in the viscoelastic behavior suggested differences in internal dynamics.

### Self-Healing and Moldability

Due to the reversibility of supramolecular interactions, we wanted to test the self-healing behavior of these hydrogels. The self-healing behavior was visualized by cutting a macroscopic gel disc in two pieces and then compressing the two pieces of gels together. All hydrogelators showed excellent self-healing properties under a few minutes at room temperature (Figure 2B and Figure S55). Next, we studied the moldability of hydrogels. The gel was transferred from a round mold to a square mold. For hydrogelator **C**_**6**_**C**_**6**_, the hydrogel flowed quickly, under 1 min, and filled the square mold (Figure 2B and Figure S55). For hydrogelators **C**_**6**_**C**_**12**_ and **C**_**12**_**C**_**12**_ (Figure 2B), we had to compress the gel using a spatula to take the shape of a square. This suggests the responsive nature of hydrogels to external forces and the ability to adapt to different shape under stress. Such hydrogels with the ability to take the desired shape may find applications to fill the voids resulting from damaged tissues after injury or cavities after surgical operations.

Self-healing behavior was quantitatively investigated using oscillatory rheology by applying high and low strain shear rupture cycles. The first strain sweep was run at 1 rad/s to determine rupture strain (*G*″ higher than *G*′); hydrogelator **C**_**6**_**C**_**6**_ behaved like a liquid (*G*″>*G*′) at this frequency, and hydrogelators **C**_**6**_**C**_**12**_ and **C**_**12**_**C**_**12**_ showed rupture strains around 100% and 20%, respectively (Figure 2C). After applying a strain of 400% to fully rupture the hydrogels, the storage moduli recovered 80% and 100% for hydrogelator **C**_**6**_**C**_**12**_ and **C**_**12**_**C**_**12**_, respectively, under tens of seconds upon removal of the high strain (Figure 2D). We also observed that **C**_**12**_**C**_**12**_ increases slightly in storage modulus after each strain cycle, which perhaps could be due to slippage of the material at such high strains or due to residual stresses in the material. Rapid recovery of the storage moduli indicates quick self-healing behavior, dynamicity, and reversibility of cross-links in BTA hydrogelators.

### Cell Viability

Using the newly developed methodology, we were able to create scalable and tunable fibrous hydrogels which could be considered as suitable ECM mimics. Ultimately, we would like to use this methodology and similar hydrogel architectures for 3D cell culture and tissue engineering applications. Though dilute solutions of BTAs have been used in the presence of cells before,^45^ BTA materials and hydrogels have only limitedly been investigated for their cytocompatibility^11^ and never in 3D encapsulation. Consequently, we wanted to quickly explore cell encapsulation and viability in these materials. Dynamic and viscoelastic matrices have been shown to greatly affect chondrocytes proliferation and cartilage like matrix production.^46,47^ We chose to use chondrocytes (ATDC5), as they are important cells for the investigation of enhanced chondrogenic differentiation and a cell line model to study cartilage tissue regeneration.^48^ ATDC5s could be encapsulated within 10% (w/v) gels via a mixing protocol that leverages the self-healing nature of the hydrogels. Cells had round morphologies in the hydrogels, and we observed that cells were able to form multicellular aggregates within gels over 4 days. This aggregation is likely due to the dynamic nature of the supramolecular hydrogels; however, erosion of the hydrogels could also be partly responsible.

**Figure 3:**
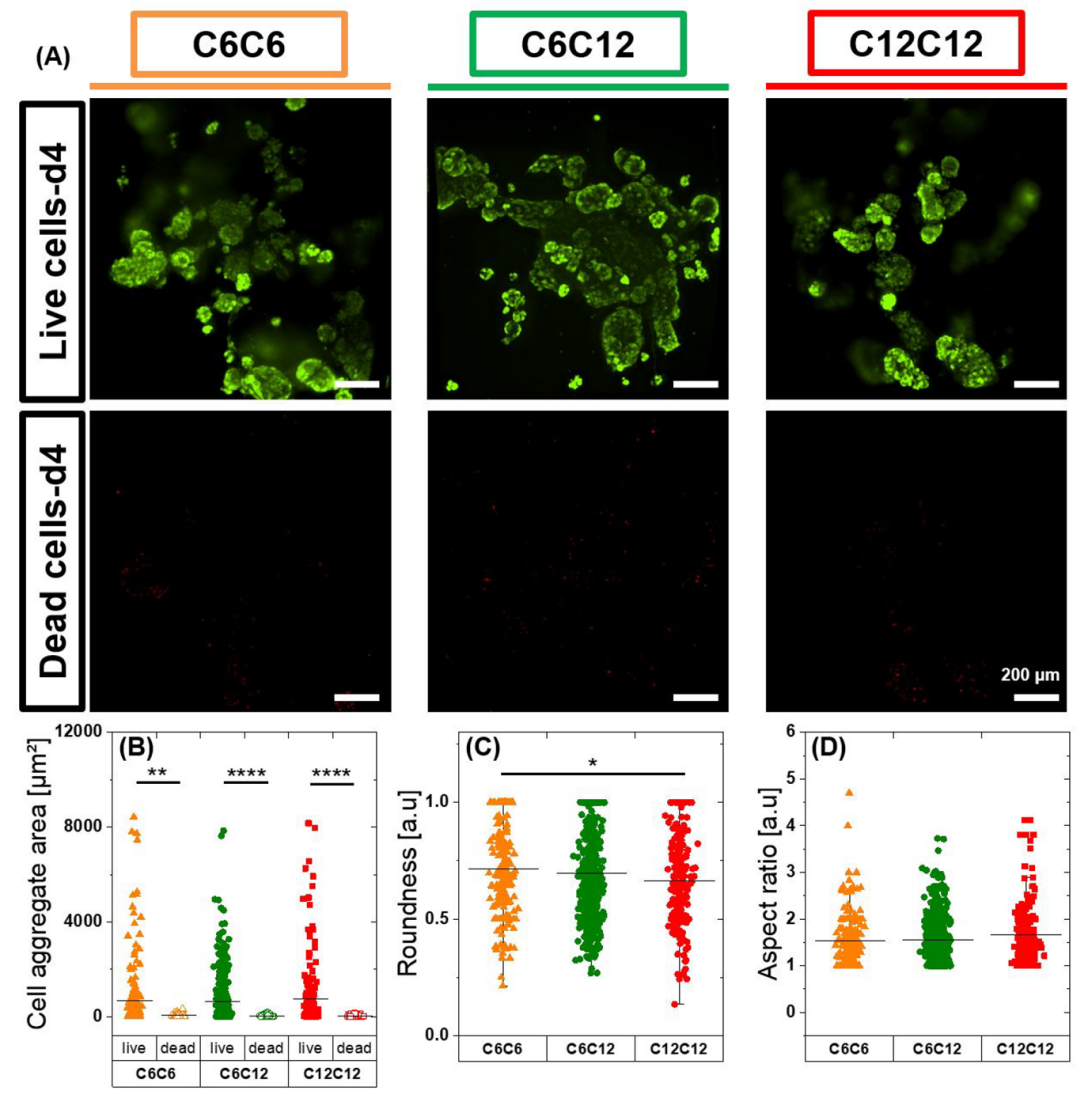
Synthesized BTA hydrogels show good cytocompatibility. (A) Chondrocytes (ATDC5) were encapsulated within gels and stained after 4 days in culture. Green color represents live cells, and red color represents dead cells. Scale bar: 200 μm. (B–D) Live and dead stack images were flattened and using image J. (B) cell aggregates area, (C) roundness of aggregates, and aspect ratio were calculated. Statistical significance was determined using one-way ANOVA (Origin software), and means comparison was analyzed using the Tukey post test. *p < 0.05, **p < 0.01, ***p < 0.001, ****p < 0.0001.

To investigate cell viability, we utilized live–dead staining where cells were stained with calcein (live, green) and ethidium homodimer (dead, red) after 4 days in culture. Since cells formed multicellular aggregates and quantification by counting single cells was not feasible, we calculated the area of live and dead cells. The large majority of cells (>95% cells by area) stayed alive within these gels after mixing and during 4 days of culture (Figure 3A and Figure S56). Cell aggregate area mean values of live cells were also significantly higher (Figure 3B) than dead cells indicating good viability. Aggregate morphology analysis showed that aggregates have morphologies between round and elongated and that all hydrogelators showed similar aspect ratio mean values, and as such, no significant differences were found among hydrogelators (Figure 3C–D). These initial cell viability tests show that this methodology and these hydrogelator structures possess no major cytotoxicity concerns.

## Conclusions

In this work, we have developed a new synthetic strategy to create desymmetrized BTA molecules, macromolecules, and ultimately functional materials. We employed an activated ester desymmetrization methodology, which was economically viable, mild, and fast. We found that BTE-F_5_Ph was the most stable activated ester and offered a flexible synthon for the creation of BTA derivatives with different side-arms and several functionalities. The yield for BTA monosubstituted, disubstituted, and trisubstituted derivatives was high and above statistically expected results for almost all reactions. Unlike existing synthetic approaches to BTAs, **3** offers a flexible desymmetrization strategy; intermediates are stable, solid, and do not require protection, starting materials can be recovered, and intermediates can be stored for further multifunctional BTA synthesis.

These BTA monomers offer the potential to be incorporated into a variety of BTA materials via modular mixing e.g. for tuning bioactivity and creation of polymeric BTA hydrogelators. Utilization of some of our desymmetrized BTAs allowed the creation of a small set of telechelic BTAs on multigram scale with high yield. One of the major advantages this strategy offers is that now BTA functionalized materials can be made with two different external arms on the BTA (i.e., hydrogelator C_6_C_12_).

The telechelic BTA macromolecules synthesized in this study showed fibrous morphology in dilute solution, akin to the fibrous assemblies within the native ECM. At higher concentrations, the telechelic BTA macromolecules form stable hydrogels and offer similar equilibrium storage moduli, but show differences in dynamic mechanical properties. Furthermore, these BTA hydrogels are moldable as well as self-healing and show good cytocompatibility (chondrocytes).

This methodology is envisioned to be highly tolerant to sensitive functional groups and favors the creation of linear and divergent libraries quickly within the lab. With the ability to create more complex molecules, the ability to create more complex supramolecular polymers becomes more attainable. While we chose to investigate the ability to create ECM mimicking BTA fibrous hydrogels, this approach should be amenable to numerous applications where BTA serves as a supramolecular motif. With this new scalable strategy, we can confidently explore more complex BTA hydrogels in a variety of tissue engineering applications, and more complex BTA architectures can be accessed for a variety of studies and applications utilizing supramolecular assembly.

## Supporting Information Available

Synthesis of small molecule BTAs and polymeric BTAs, ^1^H and ^13^C NMR spectra, MALDI-TOF, GPC, Nile Red, cryo-TEM, rheology and cell viability; DFT calculations for molecule **9** and **10** describing molecular origin of selectivity (PDF)
