# Supplementary material for: Desymmetrization via Activated Esters Enables Rapid Synthesis of Multifunctional Benzene-1,3,5-tricarboxamides and Creation of Supramolecular Hydrogelators
Source: J Am Chem Soc. 2022 Feb 23;144(9):4057–70. doi: 10.1021/jacs.1c12685 (PMC8915260; doi:10.1021/jacs.1c12685)
Supplement: Supplementary file 1 — ja1c12685_si_001.pdf [file ja1c12685_si_001.pdf]

Desymmetrization via Ativated Esters Enables Rapid Synthesis of Multifunctional Benzene-1,3,5-tricarboxamides and Creation of Supramolecular Hydrogelators

Shahzad Hafeez,[1] Huey Wen Ooi,[1] Dennis Suylen,[2] Hans Duimel,[3] Tilman M. Hackeng,[2] Clemens van Blitterswijk,[1] and Matthew B. Baker[1,*]

1 Department of Complex Tissue Regeneration, MERLN Institute for Technology Inspired Regenerative Medicine, Maastricht University, P.O. Box 616, 6200 MD Maastricht, the Netherlands;

2 Department of Biochemistry, Cardiovascular Research Institute Maastricht (CARIM), Maastricht University, P.O. Box 616, 6200 MD Maastricht, the Netherlands;

3 Maastricht MultiModal Molecular Imaging Institute, Maastricht University, P.O. Box 616, 6200 MD Maastricht, the Netherlands.

* Correspondence: m.baker@maastrichtuniversity.nl

Materials and Methods:

Abbreviations: Dichloromethane (DCM), Tetrahydrofuran (THF), N,N-Diisopropylethylamine (DIPEA), Benzene-1,3,5- tricarbonyl trichloride (BTCl), Dimethyl sulfoxide (DMSO), tetrahydrofuran (THF), tris(perfluorophenyl) benzene-1,3,5-tricarboxylate (BTE-F_5Ph), 5-Norbornene-2-methylamine (5Nb-2MA), carbonyl diimidazole (CDI), Nuclear Magnetic Resonance (NMR), Matrix-Assisted Laser Desorption/Ionization Time-of-Flight (MALDI-TOF), critical gelation concentration (CGC), Benzene-1,3,5- tricarbonyl trichloride (BTCl), Thin-layer chromatography (TLC)

General:

All chemicals were purchased from commercial sources and used as received unless stated otherwise. 5-Norbornene-2-methylamine (mixture of isomers, TCI Chemicals), N, N-Diisopropylethylamine (DIPEA) solution was dried using sodium hydroxide pellets. Hexylamine solution was dried using powder magnesium sulfate drying agent. Dichloromethane (DCM) and Tetrahydrofuran (THF) employed for desymmetrization reactions were ≥99% anhydrous. Thin-layer chromatography (precoated 0.25 mm, 60-F254 silica gel plates from Merck) was used to follow the reactions, and silica gel (40–63 μm, 60 Å from Sigma-Aldrich) flash chromatography was run to isolate the pure compounds.

Nuclear Magnetic Resonance (NMR) analysis and sample preparation.

NMR analysis was performed using a Bruker Ascend 700MHZ NMR Spectrometer for proton and 176MHZ for carbon. Data was analyzed using the TopSpin 3.5 and spectra were calibrated to either chloroform ($CDCl_3$: 7.27ppm) or d_6-DMSO (2.5ppm). for sample preparation, 2–7 mg sample was dissolved in either deuterated chloroform or deuterated DMSO (roughly 700 ul). Chemical shifts are given in ppm (δ) values relative to the residual solvent. Splitting patterns are labelled as s, singlet; bs, broad singlet, d, doublet; dd, double doublet; t, triplet; q, quartet; p, pentet; m, multiplet and b stands for broad.

Matrix-Assisted Laser Desorption/Ionization Time-of-Flight mass spectrometry:

Matrix-Assisted Laser Desorption/Ionization Time-of-Flight (MALDI-TOF) mass spectrometry (MS) was performed on an Applied Biosystems 4800 MALDI TOF/TOF system in reflector positive mode. α-cyano-4-hydroxycinnamic acid in 50% water / 50% acetonitrile containing 0.1% TFA was used as a matrix. Samples were spotted on the plate and dried in the open air before analyses. Chloroform was used as a solvent in case of low solubility in acetonitrile.

Nile red studies:

For each hydrogelator three samples with final concentration of 1 mg/mL, 2 mg/mL and 5 mg/mL were prepared. To start with solid polymer was weighed in a glass vial and 50 μL methanol was added to it for

dissolving hydrogelators molecularly so that there are no stacking of BTA units. A little bit of heating is required especially for $\mathbf{C_{12}C_{12}}$ to fully dissolve. Later, 950 µL of water was added to the glass vial. Heating and cooling cycles were repeated until solution become transparent and let BTA molecule age overnight. Next day, 200 µL from each solution was transferred to transparent bottom black well plate and added to it 3.2 µL of Nile red (from stock solution of 1 mg/10mL= 315 µM) resulting in final Nile red concentration of 5 µM. The well plate was incubated for 30 minutes in the dark at room temperature and fluorescence emission spectra were recorded.

Cryogenic transmission electron microscopy (cryo-TEM):
All samples were prepared at 10 mg/mL. The polymer was dissolved in 50 µL of methanol with the help of heating and then 950 µL of Milli-Q water was added to the hydrogelator solution. The sample solution was heated until the sample became transparent and was allowed to cool at room temperature and age overnight (16–24 hours) before imaging. In preliminary studies, samples were also prepared in Milli-Q water using the heating-cooling method and we only found a few sheets like structures for $\mathbf{C_{12}C_{12}}$, otherwise mostly precipitated big undefined structures. Using cosolvent methanol and heating for dissolving BTA hydrogelator in minimal methanol and then mixing with water leads to observable self-assembled structures.

The sample vitrification procedure was carried out using an automated vitrification robot (FEI Vitrobot™ Mark IV). Cryo-TEM grids, R2/2 Quantifoil Jena grids, were purchased from Quantifoil Micro Tools GmbH. Prior to the vitrification procedure (3 µL aliquots, blotting time varied from 3 s to 4 s, -5 mm blotting offset, 100% relative humidity) the grids were surface Glow discharged using a Cressington 208 carbon coater operating at 7 mA for 30 s. The cryo-TEM experiments were performed on a FEI Arctica 200KV microscope. The Arctica is equipped with a FEG operating at 200 kV and the images were recorded using a Falcon III camera.

Hydrogel formation:
Solid polymer was weighed in a glass vial and water was added to make the final concentration 10% (w/v). The glass vial was heated until the polymer solution became turbid, vortexed, and then the solution was allowed to cool until it turns into a gel. Heating-cooling cycles were repeated 3x. Subsequently, the hydrogel was placed on a heating plate, the temperature was raised to 80 °C, followed by decreasing the temperature to 20 °C and letting the gel cool while the temperature was being monitored on the digital temperature controller.

Critical gelation concentration (CGC) determination: Starting from 10% (w/v), the hydrogel was diluted to half by adding water and performing a vial inversion test after 24 hours. The vial was inverted and hydrogel flow was observed under 30 seconds. If hydrogel did not flow in 30 seconds, it is a gel and if the hydrogel started to flow under 30 seconds it is considered not a gel.

Macroscopic self-healing and moldability:
The hydrogel was formed as mentioned above ("hydrogel formation" heading) and a few µL of food-grade water-based liquid colorant was added on top of the hydrogel to give hydrogel a color for better visual presentation. Hydrogelator $\mathbf{C_6C_6}$, $\mathbf{C_6C_{12}}$, and $\mathbf{C_{12}C_{12}}$ are colored in yellow, green, and red. Hydrogels were placed in a round mould, divided into two parts, and then placed together and pressed with a spatula for the self-healing test. Self-healing was determined successful if two pieces of hydrogel merged to give a single hydrogel that is stable and did not fall apart when lifted under forces of gravity. After a few minutes in a closed container under a humid environment, the gels were lifted using a spatula and hydrogelator $\mathbf{C_6C_{12}}$ and $\mathbf{C_{12}C_{12}}$ can be lifted successfully as a single piece of the hydrogel. Hydrogelator $\mathbf{C_6C_6}$ could not since it is a weak gel and started to flow like viscous liquid when handled with the spatula and lifted under the gravity force. However, hydrogelator $\mathbf{C_6C_6}$ did show self-healing properties.

For moldability, the gel was taken from a round mould, placed in the square mould, and pressed gently with a spatula to adjust to the new shape.

Mechanical properties:

Rheological measurements were performed using a DHR-2 rheometer using a 20 mm cone-plate geometry with a 2.002° angle. All measurements were performed at 20 °C. First, the linear viscoelastic region was determined by performing a strain sweep on the 10% (w/v) hydrogels. We found that storage and loss modulus are independent of strain below 10% strain and that hydrogels from $\mathbf{C_6C_{12}}$ and $\mathbf{C_{12}C_{12}}$ clearly showed hydrogel dominant behavior (G'>G"), while the hydrogel from $\mathbf{C_6C_6}$ shows liquid dominant behaviour at this frequency (1 rad/s, **Figure S54**). A rejuvenation process was carried out to remove any mechanical history within gels. We choose a 400% strain amplitude at frequency 1 rad/s. Strain amplitude is well into the non-linear regime of the samples. Following the rejuvenation process, a time sweep at 1% strain was carried to observe the ageing kinetics and allow sample equilibration. Subsequently, a frequency sweep was carried out from 0.01 rad/s to 627 rad/s at 1% oscillation strain followed by oscillatory strain amplitude sweeps 1 to 1000% at an angular frequency of 1 rad/s. For self-healing measurements, a step-strain experiment was carried out, the strain was varied between 400% and 1% (at 1 rad/s) for rupture and recovery phase of the sample.

ATDC5 chondrocytes cell culture:

ATDC5 were expanded at 37 °C in humidified incubator using a growth medium consisting of high glucose Dulbecco's modified eagle medium (DMEM) with 10% fetal bovine serum, 1% penicillin/streptomycin. The medium was changed after three days and ATDC5 were used for viability studies between 75–85% confluency.

Cell encapsulation in BTA supramolecular hydrogelators

ATDC5 (chondrocytes) were encapsulated in 200 μL BTA hydrogel at a concentration of 5 million cells per mL of hydrogelator. For encapsulation of cells within hydrogels, 100 μL of hydrogel was transferred to the well plate, centrifuged to form a uniform layer at the bottom of the 48-well plate (non-treated for cell culture). Cell suspension in 25 μL of media was spread on top of the gel and centrifuged at 80 RCF for cells to sediment in a gel. After centrifugation, the other 100 μL of the hydrogel was added on top of the hydrogel and gently mixed using a spatula. On top of each gel, 200 μL media was added and the gels were incubated at 37°C. Dulbecco's Modified Eagle's Medium-F12 (DMEM-F12, low glucose) supplemented with 10% (v/v) fetal bovine serum (FBS) and 1% (v/v) P/S) was used in this study.

ATDC5 viability in BTA supramolecular hydrogelators:

LIVE/DEAD™ Viability/Cytotoxicity Kit (Thermo Fisher Scientific) was used for evaluating cell viability. For live-dead staining, the hydrogels were transferred into a 35 mm glass-bottom dish (ibidi, Germany), washed with PBS, and a solution of calcein-AM (final concentration 1 μM) and ethidium homodimer-1 (final concentration 2 μM) was added to each gel. The gels were incubated for 45-60 minutes in the dark at 37 °C and imaged using an inverted fluorescence microscope (Nikon Eclipse Ti-e) under the conditions of 37 °C and 5% CO_2. Images were analyzed using ImageJ.

Benzene-1,3,5-tricarbonyl trichloride (BTCl) desymetrization using hexylamine:

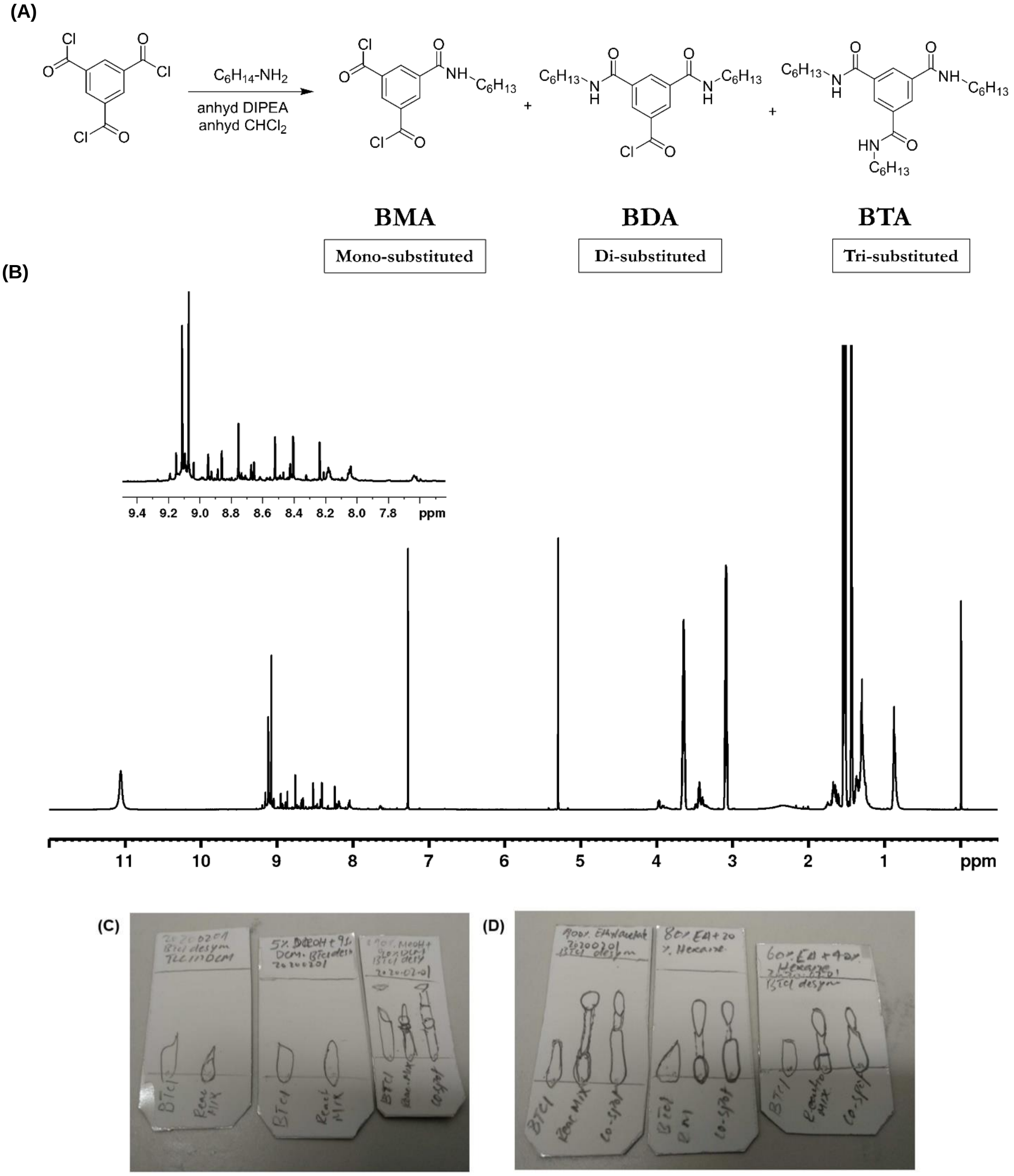

Figure S1: A) BTCl desymmetrization reaction scheme using hexylamine as a model molecule, B) ^{1}H NMR of BTCl desymmetrization using hexylamine. Separation using thin-layer chromatography: C) Left: in DCM, middle: in 5% (v/v) MeOH+95% (v/v) DCM, right: 10% (v/v) MeOH+90% (v/v) DCM. D) Left: in ethyl acetate (EA), middle: in 80% EA (v/v)+20% (v/v) hexane, right: 60% (v/v) EA+40% (v/v) Hexane.

Table S1: Nitrophenol solubility

Solvent	BTCl	$3NO_2PH$	$4NO_2Ph$
DCM	S	NS	NS
Chloroform	S	P	P
THF	S	S	S
DMSO	S	S	S

DCM=Dichloromethane, THF= tetrahydrofuran, DMSO= dimethylsulfoxide, NS=not soluble, P= partially soluble, S= soluble. between 3–5 mg of each compound in 3–4 mL of the solvent.

Synthesis of 1-(3-nitrocyclohexa-1,3-dien-1-yl) 3,5-bis(3-nitrophenyl) benzene-1,3,5-tricarboxylate (1):

In a flame dried flask, benzene-1,3,5-tricarbonyl trichloride (BTCl, 0.1 g, 0.3 mmol, 1 equiv.) was dissolved in 4 mL of freshly dried THF (over NaOH). First, DIPEA (0.29 g, 2.2 mmol, 6 equiv.) and then 3-nitrophenol ($3NO_2Ph$, 0.26 g, 1.8 mmol, 5 equiv.) were added to the reaction flask under nitrogen atmosphere. The formation of solid crystals started to appear in just 5 minutes and the reaction solvent color turns light yellow. TLC was obtained after 1 hour of the reaction in DCM and showed three spots with R_f close to 0, at R_f 0.5 (3-nitrophenol) and 0.7 (expected product, **1**). Adding 5% methanol and running TLC moved these spots higher with R_f close to 0 and R_f at 0.6 and 0.9 but no other spot became visible. The reaction mixture was allowed to stir at room temperature for 20 hours and the color of the reaction solvent turned to dark yellow. After 20 hours, the stirring was stopped and the volatiles were removed under vacuum. The crude reaction mixture was purified by repeated crystallization: 1x using ethyl acetate/hexane (50:50) and 2x using ethyl acetate/hexane/THF (40:40:20). Molecule **1** (BTE-$3NO_2Ph$) was obtained as light yellowish solid (0.16 g, 76% yield) and stored under a nitrogen atmosphere at room temperature in a desiccator. ^{1}H NMR (700 MHz, d_6-DMSO) δ 9.10 (s, 3H), 8.41 (t, H), 8.24 (dd, H), 7.94 (dd, H), 7.83 (t, H). ^{13}C NMR (176 MHz, d_6-DMSO) 162.75, 150.64, 148.38, 135.48, 130.99, 130.60, 129.06, 121.44, 117.68. Mass of the molecule was not obtained using MALDI-TOF in a positive mode. MS (MALDI-TOF) calcd. for $C_{27}H_{15}N_3O_{12}$ $[M+H]^+$ 574.07, not obtained in reflector positive ion mode.

NOTE: $[M+H]^+$ using MALDI was not obtained, we think perhaps the molecule did not ionize.

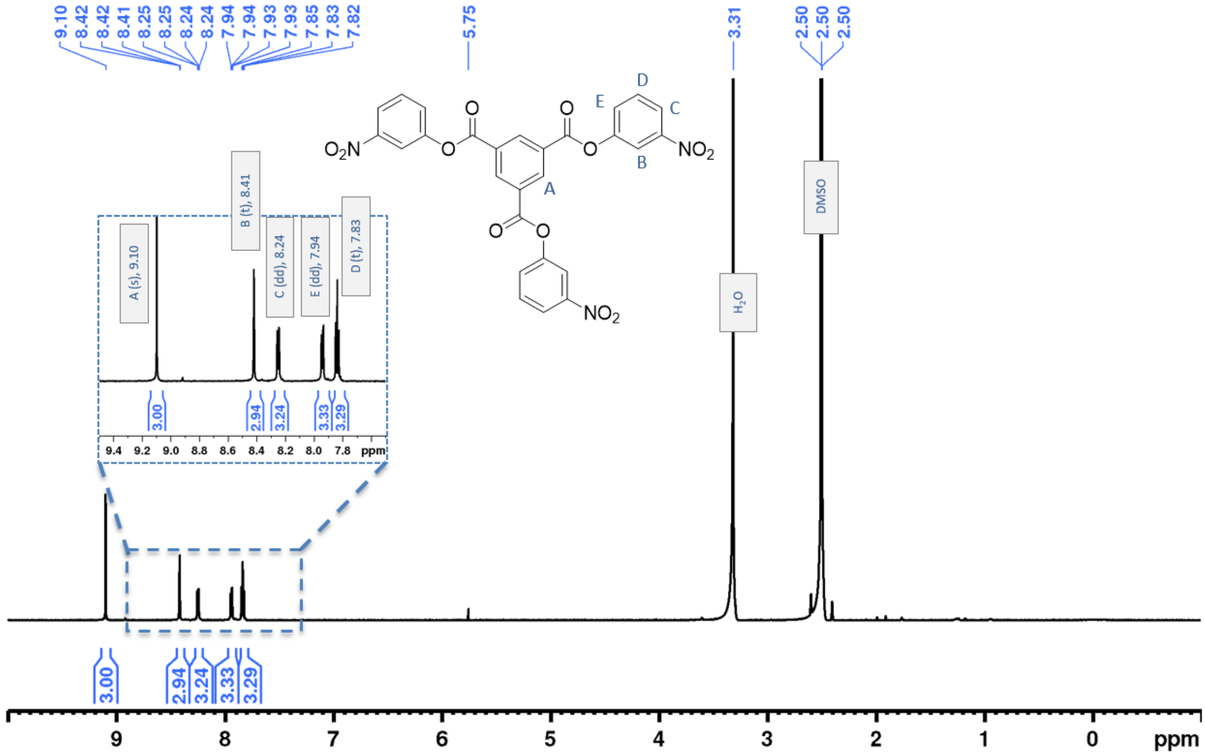

Figure S2: ^{1}H NMR (d_6-DMSO) of BTE-3NO$_2$Ph (molecule **1**)

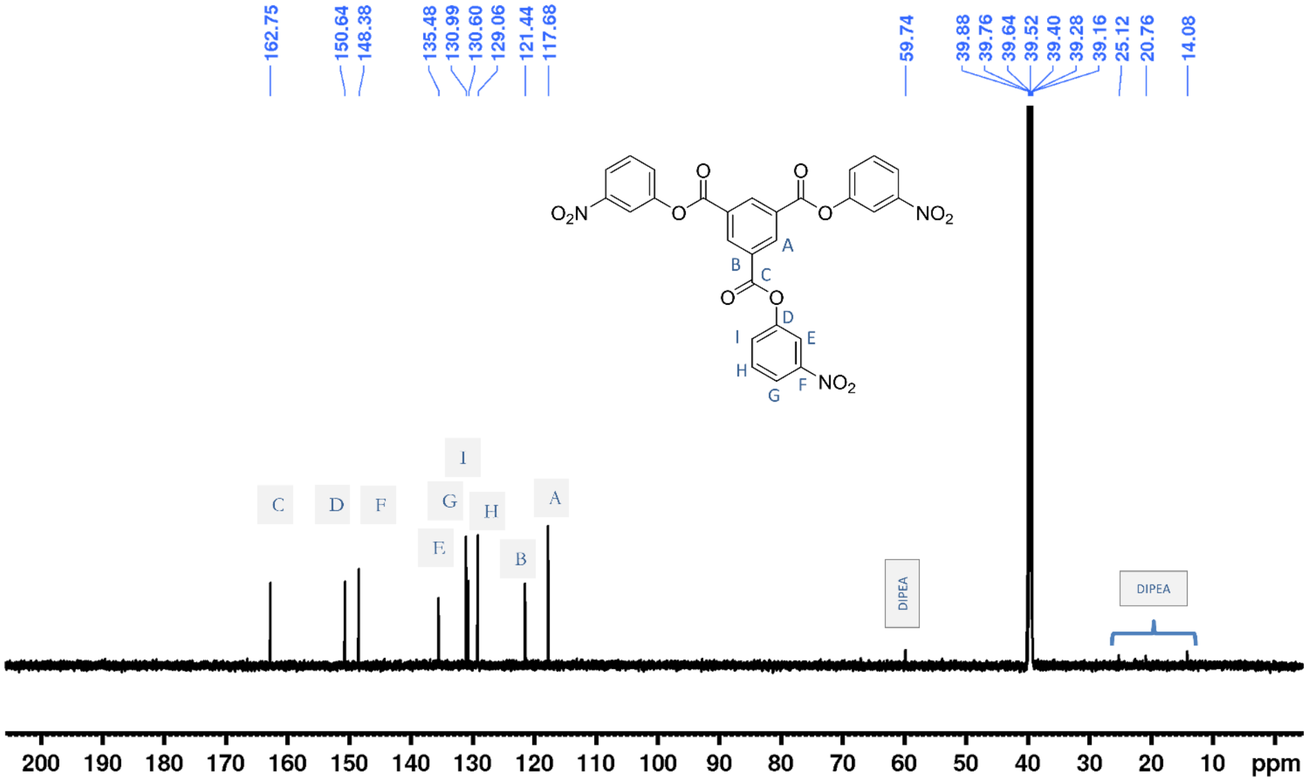

Figure S3: ^{13}C NMR (d_6-DMSO) of BTE-3NO$_2$Ph (molecule **1**)

Synthesis of 1-(4-nitrocyclohexa-1,3-dien-1-yl) 3,5-bis(4-nitrophenyl) benzene-1,3,5-tricarboxylate (2):

Benzene-1,3,5-tricarbonyl trichloride (BTCl) (0.1 g, 0.3 mmol, 1 equiv.) was weighed in a dried round bottom flask and DIPEA (0.29 g, 2.2 mmol, 6 equiv.) and 4-nitrophenol ($3NO_2Ph$) (0.26 g, 1.8 mmol, 5 equiv.) dissolved in 4 mL of freshly dried THF was added to the flask under nitrogen atmosphere through a syringe under stirring. Solid crystals started to appear in the reaction mixture in under 5 minutes. Thin-layer chromatography (TLC) in DCM showed two spots with R_f close to 0.5 (3-nitrophenol) and at R_f 0.7 (expected product, **2**). When TLC was run by adding 5% methanol, the two spots moved higher with R_f values 0.6 and 0.9 and no other spot showed up. The reaction mixture was stirred for 20 hours at room temperature. TLC showed the same spots after 20 hours. The reaction mixture was then vacuum dried and purified by repeated crystallization: 1x using ethyl acetate/ hexane (50:50) and 2x using ethyl acetate/hexane/THF (40:40:20). Molecule **2** (BTE-$4NO_2Ph$, 0.2 g, 92% yield) was obtained as creamy white/light yellowish solid. ^1H NMR (700 MHz, d_6-DMSO) δ 9.08 (s, 3H), 8.40 (d, 3H), 7.75 (d, 3H). ^{13}C NMR (176 MHz, d_6-DMSO) 162.15, 154.95, 145.39, 135.39, 130.42, 125.17, 123.15. MALDI-TOF peak for $C_{27}H_{15}N_3O_{12}$ $[M+H]^+$ 574.07 was not detected in reflector positive ion mode.

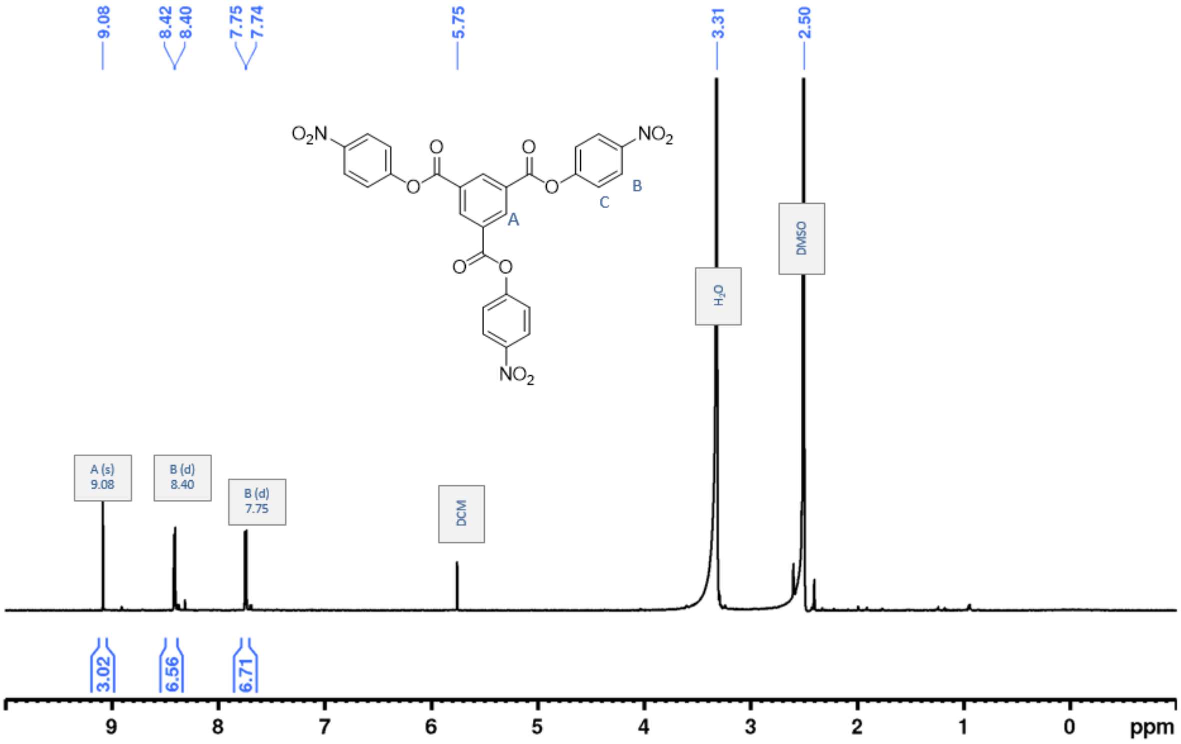

Figure S4: ^1H NMR (d_6-DMSO) of **2** (BTE-$4NO_2Ph$)

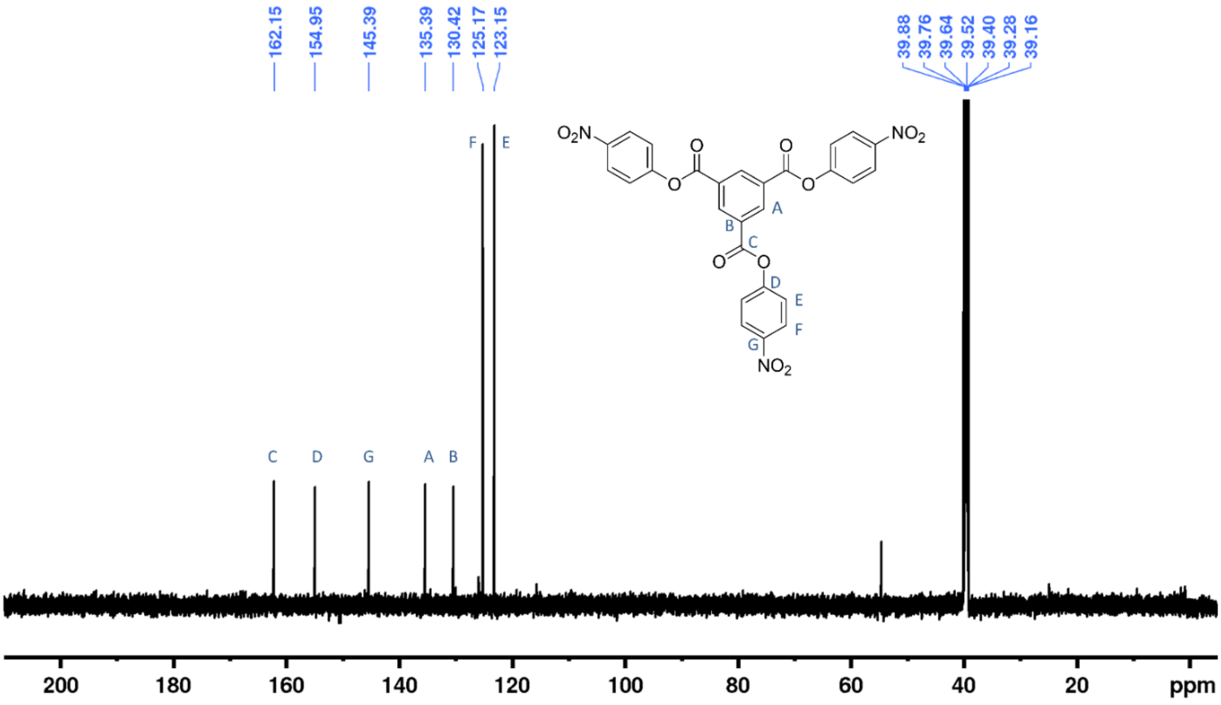

Figure S5: ^{13}C NMR d_6-DMSO of **2** (BTE-4NO_2Ph)

Table S2: Alternative workups for purification of molecule 1 and 2 and obtained % yields

Solvent for crystallization	BTE-3NO_2Ph Isolated yield	BTE-4NO_2Ph Isolated yield
**Hexane:Ethyl acetate (50/50)**	Not fully soluble with heating	Not fully soluble with heating
**THF:Ethyl acetate (40/60)**	26%	N/A
**THF:ethyl acetate:hexane (20:40:40)**	76%	92%

Synthesis of tris(perfluorophenyl) benzene-1,3,5-tricarboxylate (3):

To a three neck clean and dry 250 mL round bottom flask equipped with a stir bar under nitrogen flow, pentafluorophenol (5.56 g, 30.1 mmol, 4 equiv) was added, followed by anhydrous dichloromethane (DCM, 30 mL). The reaction solution was stirred until complete dissolution of pentafluorophenol. The reaction solution was then placed into an ice bath and allowed to cool. After cooling, dry diisopropylethylamine (DIPEA, 3.89 g, 30.1 mmol, 4 equiv.) was mixed with anhydrous dichloromethane (~80 mL) and added dropwise to the reaction solution under vigorous stirring. Subsequently, benzene-1,3,5-tricarbonyl trichloride (2.0 g, 7.5 mmol, 1 equiv.) dissolved in ~25 mL of dry DCM was added dropwise to the reaction mixture. The reaction mixture was removed from the ice bath after 30 minutes and allowed to stir at room temperature for 4 hours. TLC (ran in DCM) showed two spots at R_f close to zero and at R_f 0.9. The reaction mixture in DCM was concentrated *in vacuo*, passed through a filter paper, and then through a silica bed twice. The solvent (DCM) was removed under reduced pressure, yielding **3** (BTE-F_5Ph) as pure white solid (4.9 g, 91% yield). ^{1}H NMR (700 MHz, $CDCl_3$) δ 9.29 (s, 3H). ^{13}C NMR (176 MHz, $CDCl_3$) 160.5, 142.1, 140.9,

140.6, 139.5, 139.0, 137.8, 137.5, 129.6, 124.9, MS (MALDI-TOF) m/z: calcd. for $C_{27}H_3O_6F_{15}$, 707.97; found 708.02 $[M+H]^+$.

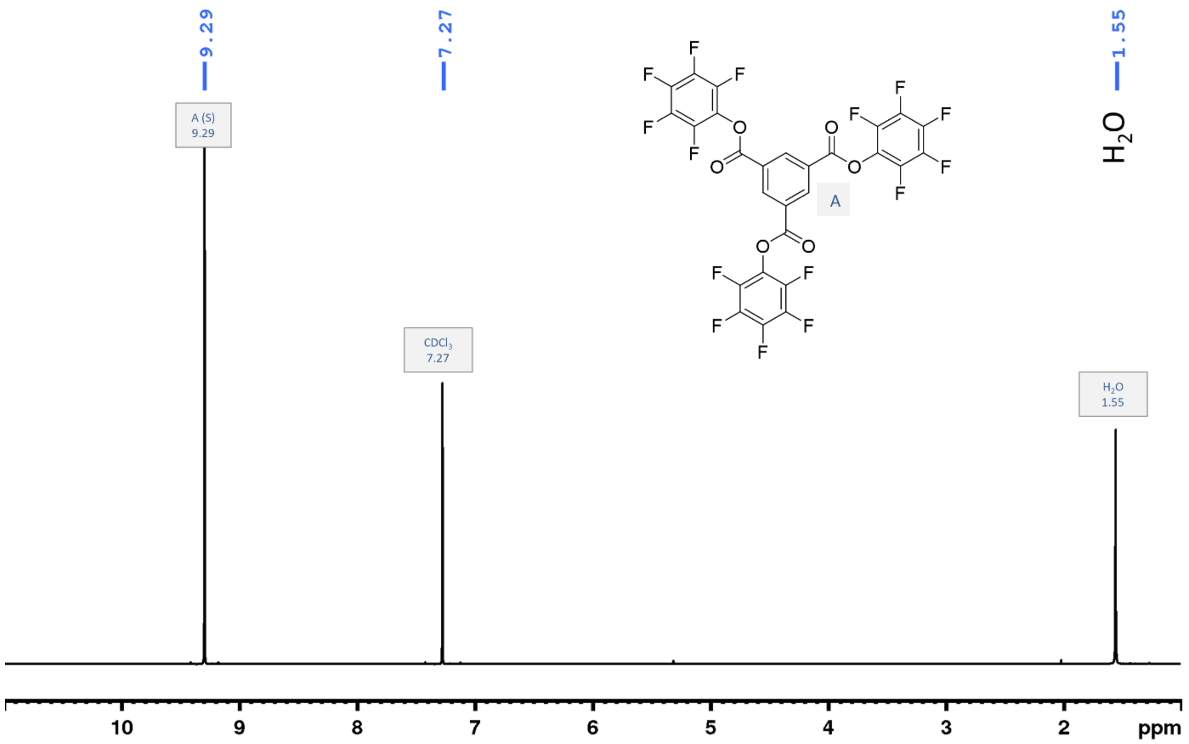

Figure S6: ^1H NMR ($CDCl_3$) of BTE-F_5Ph (molecule **3**)

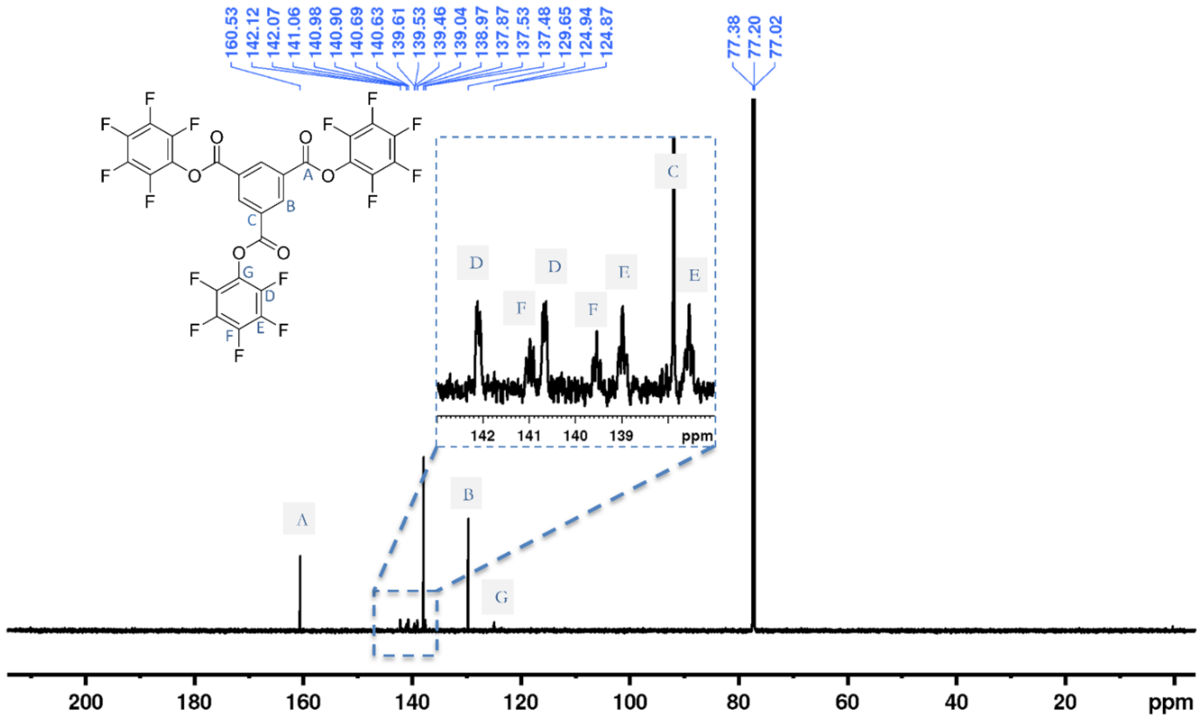

Figure S7: ^{13}C NMR ($CDCl_3$) of BTE-F_5Ph (molecule **3**)

Table S3: Workups for purification of molecule 3 and obtained % yields

Methods	Solvent	BTE-F5Ph % Yield
**Excessive washing of reaction mixture with acetonitrile**	Acetonitrile	45%
**Crystallization**	acetonitrile+hexane/chloroform	60%
**Filtration and passing through bed of silica**	Dichloromethane	91%

Table S4: NHS solubility in different organic solvents for the synthesis of molecule 4

Solvents	NHS
**DCM**	P
**Chloroform**	P
**DMF**	S
**DMSO**	S
**THF**	S

DCM=Dichloromethane, THF= tetrahydrofuran, DMSO= dimethyl sulfoxide, DMF= dimethylformamide, NS=not soluble, P= partially soluble, S= soluble. NHS compound 3–5 mg was dissolved roughly in 3–4 mL of the [illegible]

Synthesis of tris(2,5-dioxopyrrolidin-1-yl) benzene-1,3,5-tricarboxylate (4):

A 50 mL clean and dry single-neck flask equipped with a stir bar was purged with nitrogen. Benzene-1,3,5-tricarbonyl trichloride (0.1 g, 0.3 mmol, 1 equiv.) was added under nitrogen atmosphere and the flask was closed with a screw cap. Freshly dried THF (using NaOH pellets) was added to the flask using a cannula and stirred until dissolved. DIPEA (0.38 g, 3 mmol, 8 equiv.) and NHS (0.21 g, 1 mmol, 5 equiv.) dissolved in 7 mL of anhydrous THF was added drop by drop to the reaction mixture using a syringe. The resulting mixture was stirred for 20 hours and then the volatiles were removed by vacuum. TLC showed two spots at R_f equal to ~0.1 and ~0.4 (**Figure S9**); however, during separation using silica gel flash column chromatography, **4** was not obtained pure and free NHS seems to eluted with **4** (**Figure S10**, ^{1}H NMR collected fractions for R_f 0.4). Crystallization in ethylacetate: hexane (80:20) provided a greasy solid and the reaction mixture was not fully soluble even in the hot boiling solvent. TLC showed that greasy solid is not pure. No product crystallization was observed in THF: ethylacetate: hexane (~33:~33:~33).

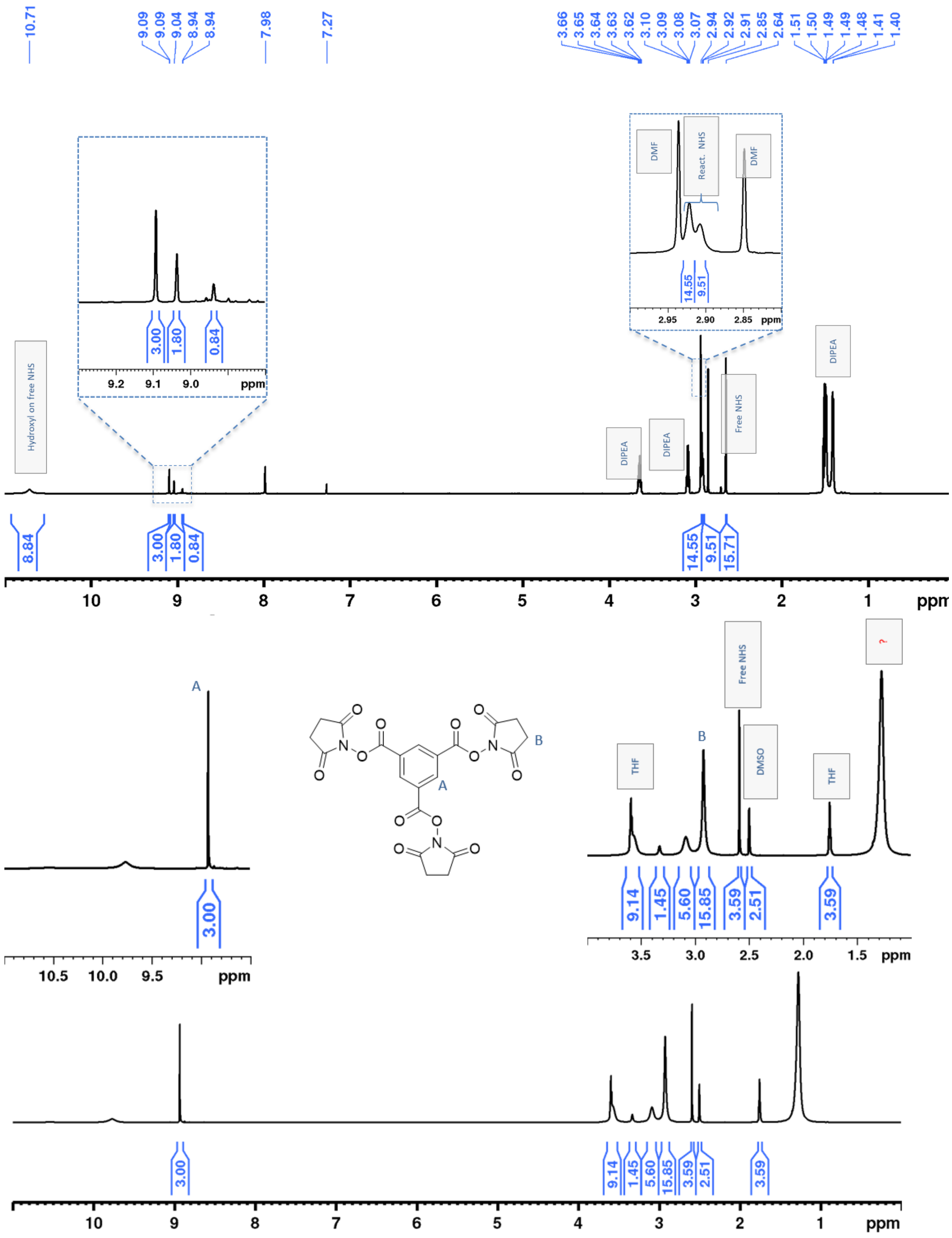

Figure S8. ^1H NMR ($CDCl_3$) of reaction mixture when reaction was run in DMF (top) to synthesize **4** (BTE-NHS). Appearance of three peak around 9 ppm suggests that this reaction produced non-symmetric BTE-NHS molecule which is not of interest for this study. ^1H NMR (d_6-DMSO) of the reaction mixture when **4** was synthesized in dry THF. Appearance of singlet for aromatic proton "A" indicates formation of symmetrical molecule **4.**

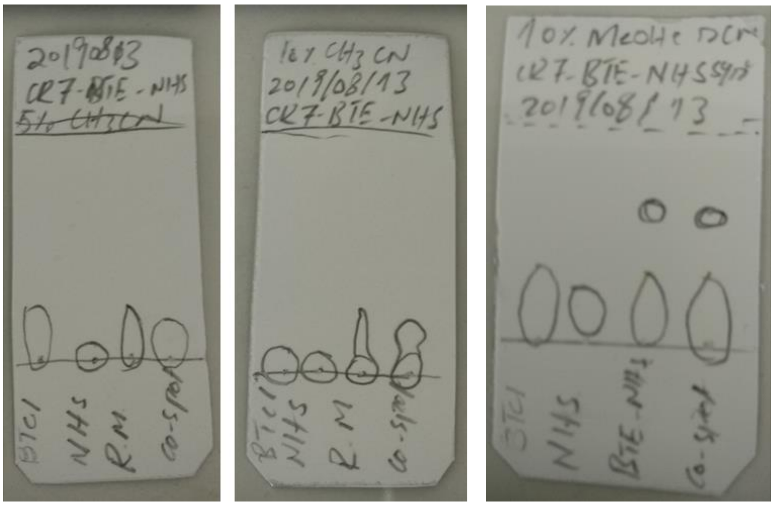

Figure S9. BTE-NHS synthesis in THF: Thin layer chromatography spots for benzene-1,3,5- tricarbonyl trichloride (BTCl), N-Hydroxysuccinimide (NHS) and reaction mixture in (Left) 5% (v/v) CH_3CN+95% (v/v) DCM, (middle) 10% (v/v) CH_3CN+90% (v/v) DCM, and (right) 10% (v/v) CH_3OH+90% (v/v) DCM.

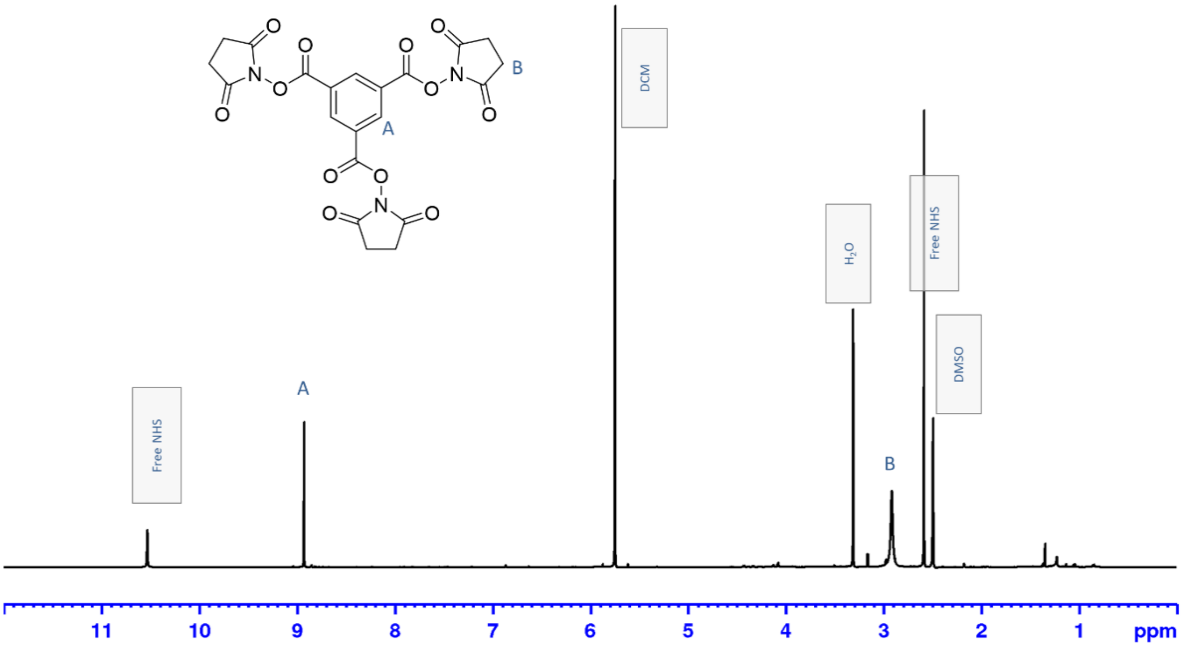

Figure S10. ^{1}H NMR (d_6-DMSO) after flash column chromatography using 10%(v/v) CH_3OH+90%(v/v) DCM showing presence of free NHS. Free NHS appears to be travelling with **4**. We were able to separate reaction mixture spots on TLC as shown above in **figure S9** but **4** was not obtained pure when separated using silica gel flash column chromatography.

Table S5: BTE-3NO$_2$Ph (1) and BTE-4NO$_2$Ph (2) solubility in different organic solvents

**Solvent**	**BTE-3NO2Ph (1)**	**BTE-4NO2Ph (2)**
Toluene	NS	NS
DMF	P	P
acetonitrile	P	P
DCM	NS	NS
THF	NS	NS
2-propanol	P	P
1,4 dioxane	P	P
Dimethyl carbonate	P	P

NS = not soluble, P = partial solubility, S = fully soluble. Each Nitrophenol compound between 2–5 mg was dissolved in ~4 mL of the solvent.

Desymmetrization reaction scheme of 1-(3-nitrocyclohexa-1,3-dien-1-yl) 3,5-bis(3-nitrophenyl) benzene-1,3,5-tricarboxylate (1):

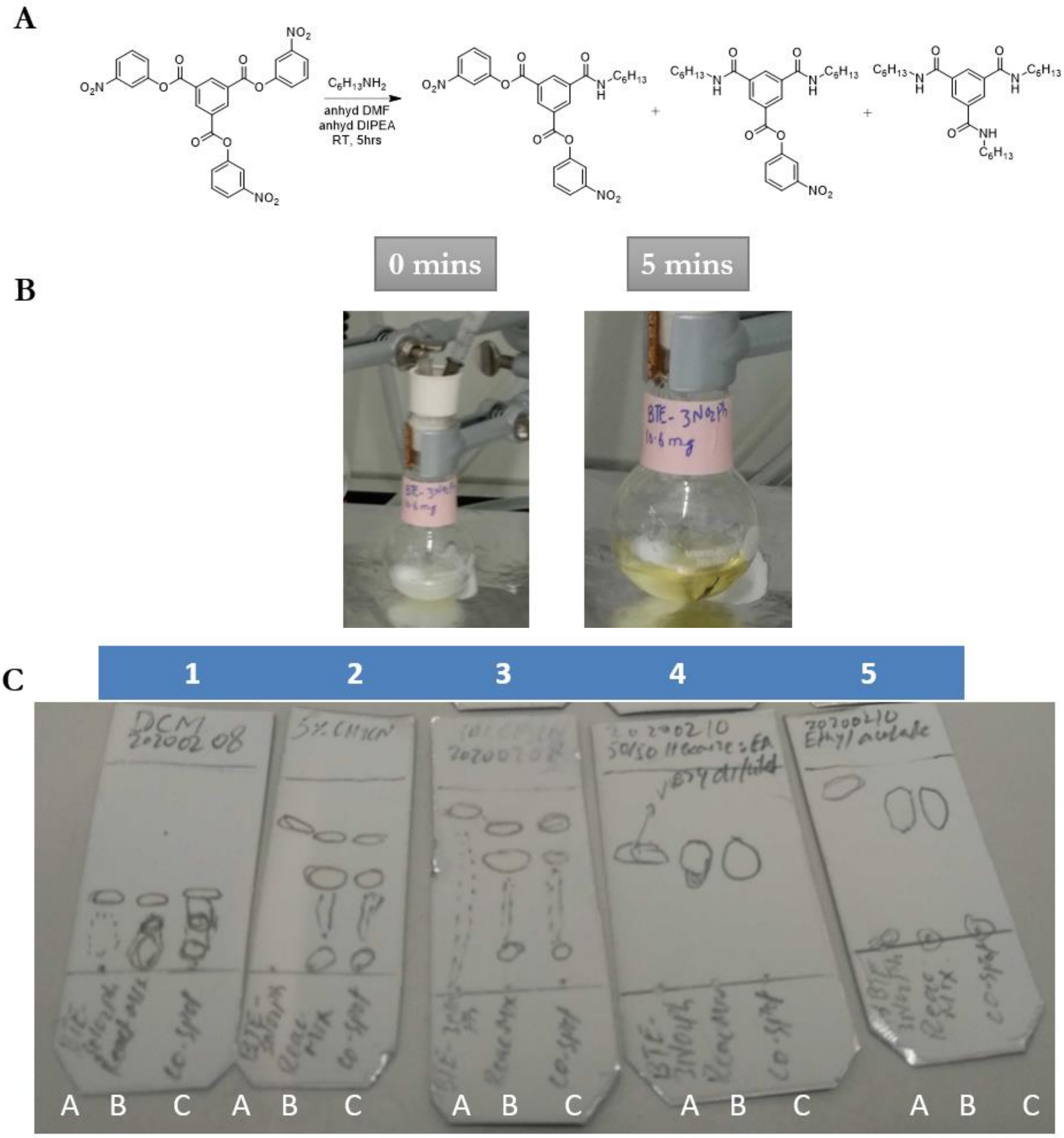

Figure S11: A) Reaction scheme of desymmetrization reaction of molecule **1** using DMF solvent. B) Molecule **1** showed partial solubility in DMF (left) and solubility increased and reaction solution became clear with reaction proceeding (right). C) TLC of the reaction mixture in DMC (labelled as 1), in 5%(v/v) CH_3CN+95%(v/v) DCM (labelled as 2), 10%(v/v) CH_3CN+90%(v/v) DCM (labelled as 3), 50%(v/v) ethylacetate+50%(v/v) hexane (labelled as 4), an in ethyl acetate (labelled as 5). Different solvent combinations were used to separate products spots formed during the reaction. Maximum three spots were visible on TLC when using solvent 10%(v/v) CH_3CN+90%(v/v) DCM. First reaction spot on TLC plate is molecule **1** (labelled as A on TLC plate), middle spot is reaction mixture (labelled as B on TLC plate, and last spot on TLC is a co-spot (labelled as C on TLC plate).

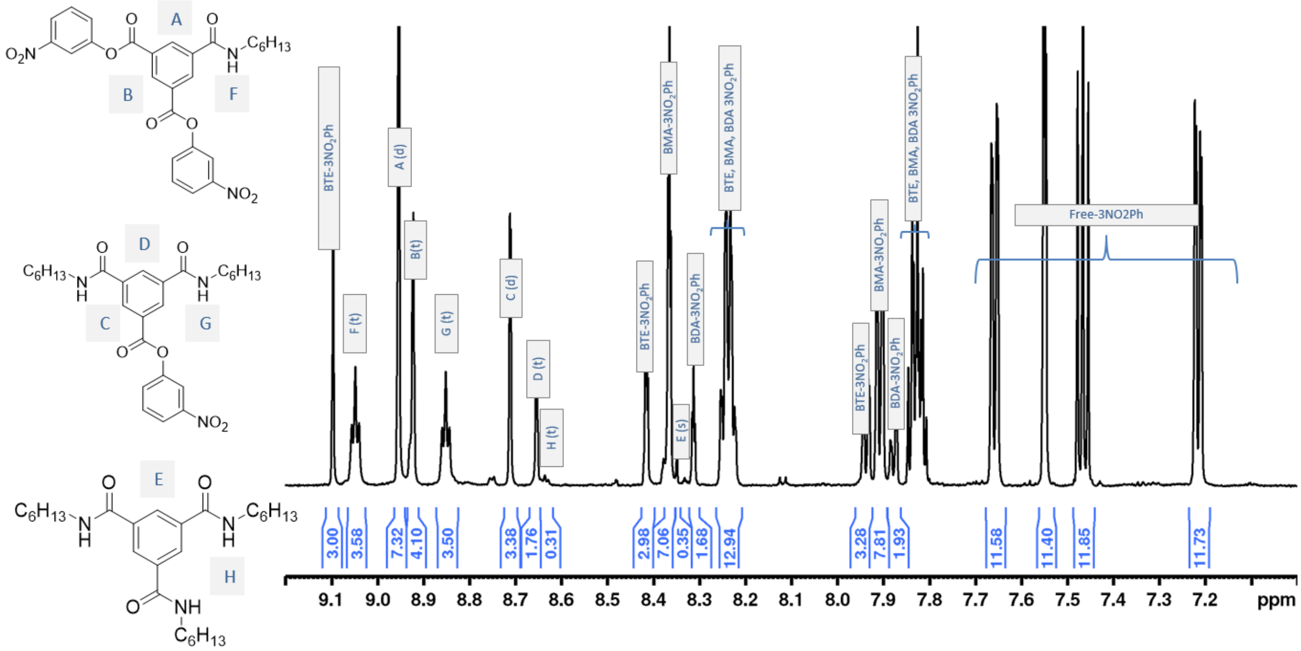

Figure S12: ^{1}H NMR (d_6-DMSO) of reaction mixture of **1** (BTE-3NO_2Ph) when desymmetrized using one equivalent of hexylamine. Different expected molecules (mono-substituted, di-substituted and tri-substituted) peaks are assigned based on chemical environment of proton and ^{1}H NMR peaks integration analysis. Zoomed in area is shown for sake of clarity of the peaks.

Synthesis of molecule 5:

Under nitrogen atmosphere, **1** (20 mg, 0.11 mmol, 1 equiv.) was dissolved in a dry round bottom flask using 3 mL anhydrous DMF. Color of the reaction mixture was milky white and **1** was not fully soluble in the reaction solvent. DIPEA (12 µL, 0.069 mmol, 0.66 equiv.) and hexylamine (4.6 µL, 0.034 mmol, 0.33 equiv.) was dissolved in 3–4 mL and added drop by drop to the reaction flask using a syringe and under nitrogen atmosphere. The reaction solution was stirred at room temperature for 5 hours. With reaction proceeding solubility of **1** increases and the color of the reaction mixture changes from milky white to light yellow and all the crystals disappeared. TLC of the reaction mixture was taken in different solvent combinations as shown above in **Figure S11** and showed a maximum of three spots with R_f close to 0.1, 0.7, and 0.9 in DCM with 10% (v/v) acetonitrile. After 5 hours reaction solvent was concentrated *in vacuo* and molecules were separated using silica gel flash column chromatography. Using dichloromethane with 5% (v/v) acetonitrile as eluent, **5** was obtained (6.9 mg, 37% isolated yield). ^{1}H NMR (700 MHz, d_6-DMSO) δ 9.03 (t, 1H), 8.94 (d, 2H), 8.91 (t, 1H), 8.35 (dd, 2H), 8.22 (t, 2H), 7.90 (dd, 2H), 7.82 (t, 2H), 3.30 (t, 2H), 1.56 (p, 2H), 1.31 (m, 6H), 0.86 (t, 3H). ^{13}C NMR (176 MHz, d_6-DMSO) δ 163.6, 163.1, 150.7, 148.39, 135.9, 133.48, 133.1, 130.98, 129.8, 129.1, 126.2, 121.3, 115.8, 30.99, 28.91, 26.16, 22.05, 13,91.

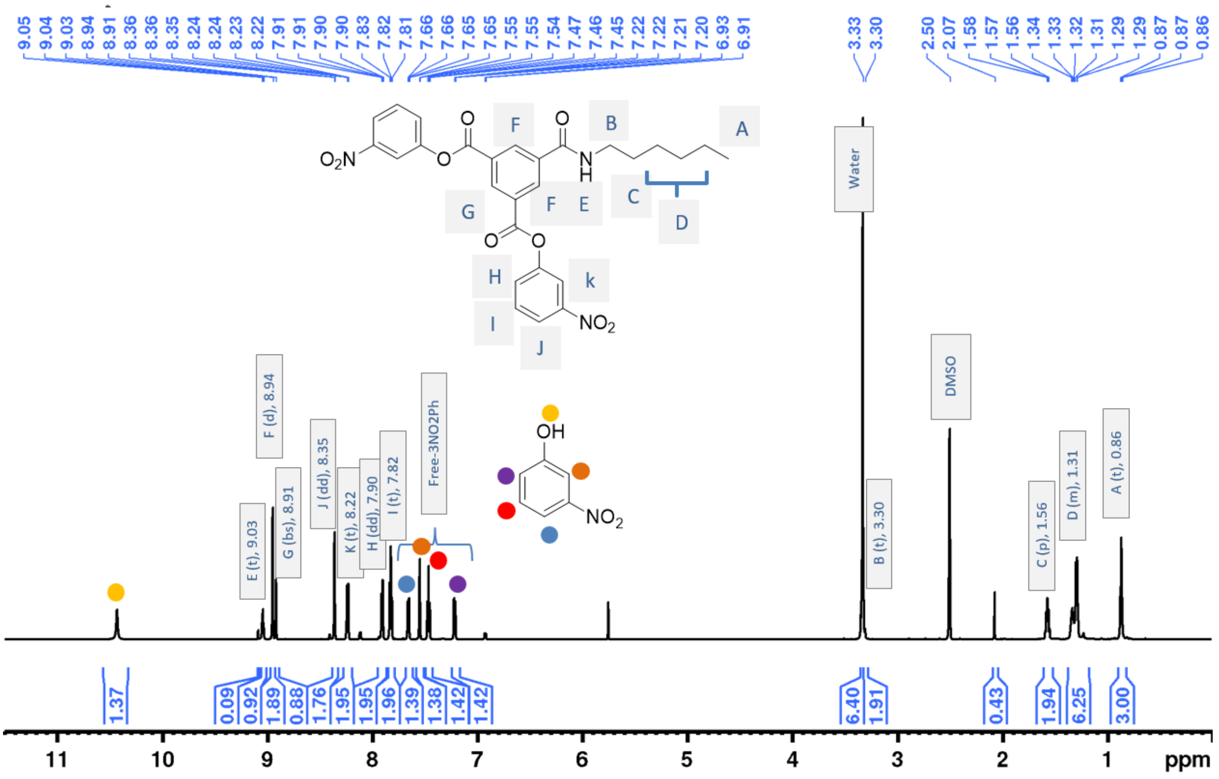

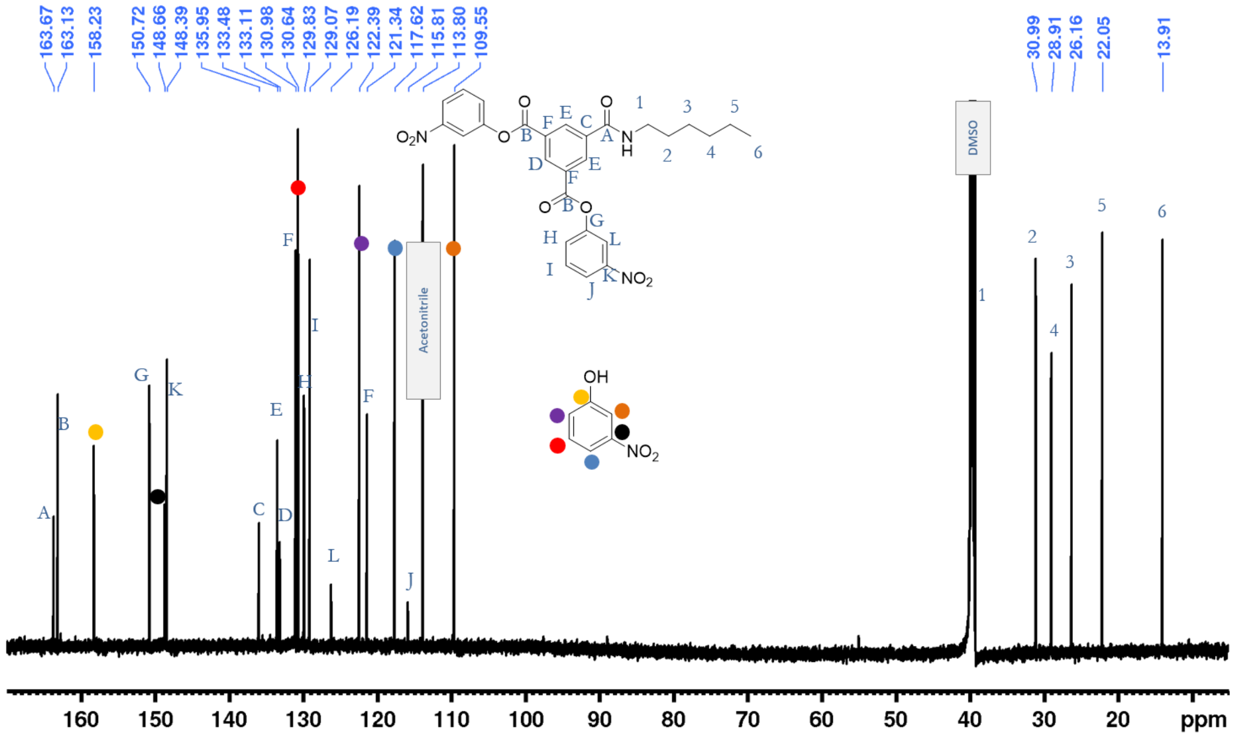

Figure S13: ^{1}H NMR (top) and ^{13}C NMR (bottom) of molecule **5** (BMA-3NO$_2$Ph). Free 3-nitrophneol which cleaved off from **1** during desymmetrization eluted with **5**. 3-nitrophenol peaks are labelled with color circles.

Synthesis of molecule 7:

Under nitrogen atmosphere, **1** (20 mg, 0.11 mmol, 1 equiv.) was dissolved in a dry round bottom flask using 5–6 mL anhydrous DMF. The reaction mixture color was milky white and non-transparent and **1** was partially soluble in anhydrous DMF. DIPEA (18 μl, 0.11 mmol, 1 equiv.) and hexylamine (9.2 μl, 0.07 mmol, 0.66 equiv.) was dissolved in 7–8 mL of anhydrous DMF and added drop by drop to the reaction flask using syringe and at room temperature under nitrogen atmosphere. The reaction mixture was stirred for 5 hours and the color of the reaction goes from milky white to yellow and clear without any solid crystals. **7** was separated using silica gel flash column chromatography using DCM with 10% (v/v) acetonitrile. ^{1}H NMR (700 MHz, d_6-DMSO) δ 8.85 (t, 2H), 8.71 (d, 1H), 8.65 (t, 2H), 8.31 (t, 1H), 8.22 (dd, 1H), 7.88 (dd, 1H), 7.81 (t, 1H), 3.26 (t, 4H), 1.55 (p, 4H), 1.29 (m, 12H), 0.86 (t, 6H). ^{13}C NMR (176 MHz, d_6-DMSO) δ 164.54, 163.57, 150.81, 148.41, 135.59, 131.48, 130.98, 130.72, 129.08, 129.00, 121.24, 117.57, 31.00, 28.95, 26.16, 22.05, 13.92.

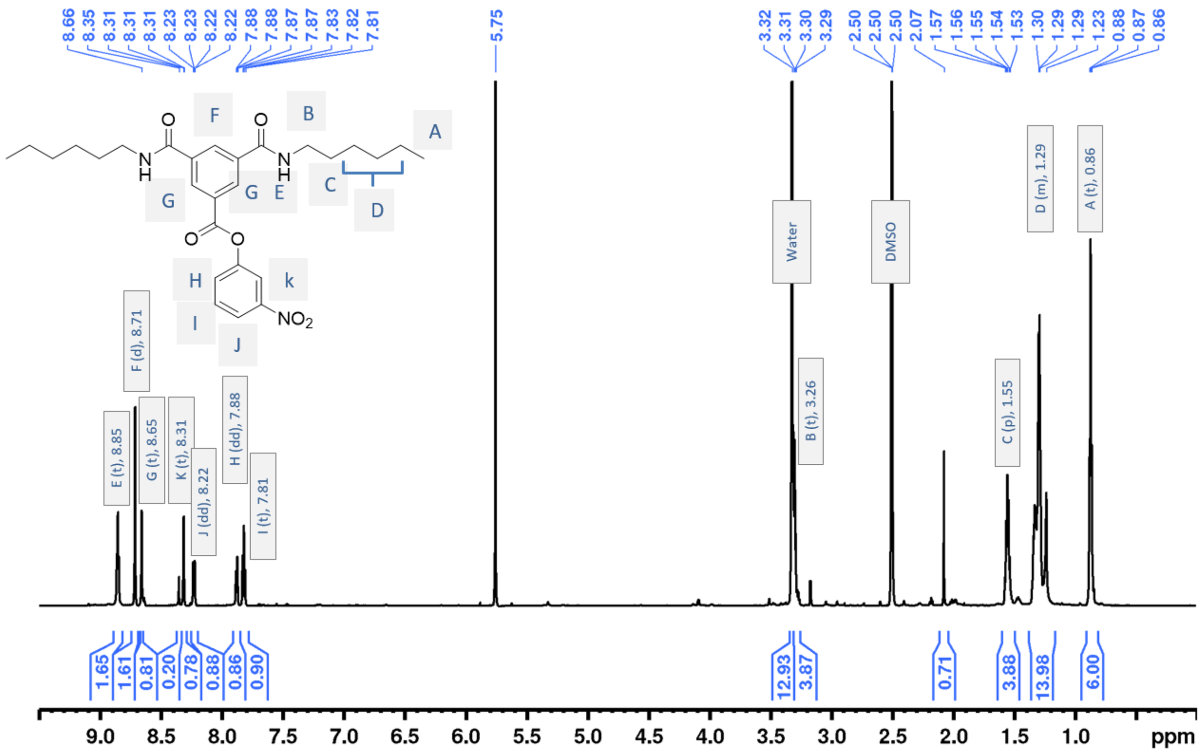

Figure S14A: ^{1}H NMR of molecule **7**

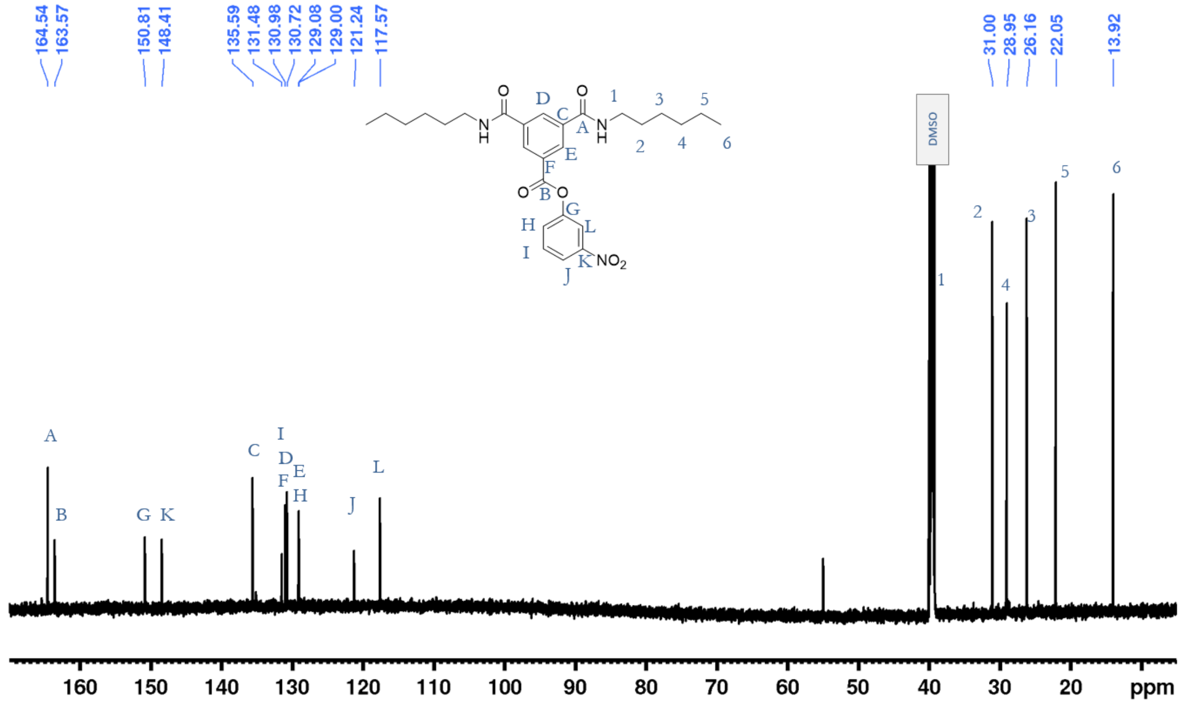

Figure S14B: ^{13}C NMR of molecule **7**

Desymmetrization of 1-(4-nitrocyclohexa-1,3-dien-1-yl) 3,5-bis(4-nitrophenyl) benzene-1,3,5-tricarboxylate (2):

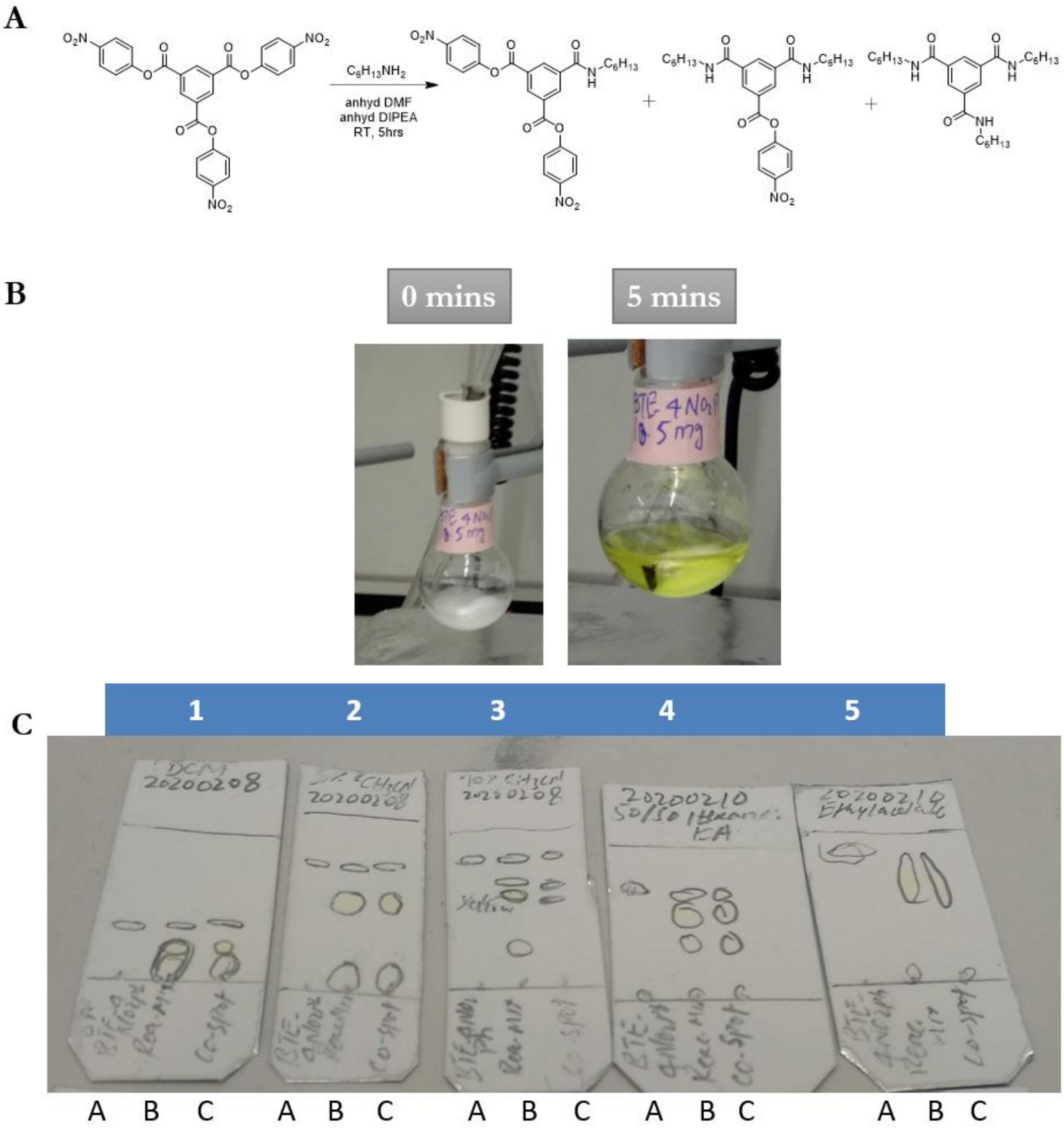

Figure S15: A) Molecule **2** desymmetrization reaction scheme and reaction was run in anhydrous DMF. B) Molecule **2** was partially soluble in DMF (reaction flask at left) and solubility increased and reaction solution become clear (right reaction flask) with reaction proceeding. C) TLC of the reaction mixture showed one spot in DCM at R_f 0.3 and three spots in DCM with 5% acetonitrile at R_f 0, 0.5, and 0.8 and four spots in DCM with 10% acetonitrile at R_f 0.3, 0.5, 0.6, 0.9. Several solvent combinations were used for separating product molecules formed during the reaction. The spot on the left on TLC plate is for molecule **2** (labelled as A on TLC plate), spot in the middle is for reaction mixture (labelled as B on TLC plate) and the spot on the right on TLC plate is a co-spot (labelled as C on TLC plate).

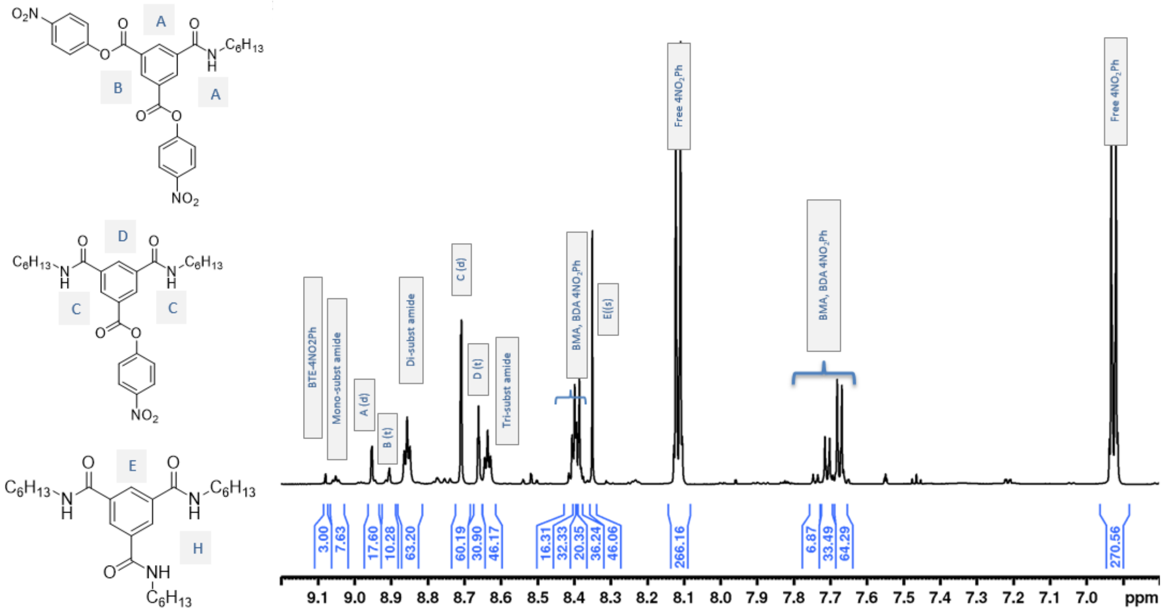

Figure S16: ^{1}H NMR of reaction mixture of BTE-4NO$_2$PH (molecule **2**) desymmetrization using hexylamine (2 equiv.). Expected molecules (mono-substituted, di-substituted and tri-substituted) peaks are assigned based on ^{1}H NMR peaks integration analysis.

Synthesis of molecule 6:

In dry round bottom flask, **2** (20 mg, 0.11 mmol, 1 equiv.) was dissolved in 3–4 mL anhydrous DMF under nitrogen atmosphere. DIPEA (12 µL, 0.069 mmol, 0.66 equiv.) and hexylamine (4.6 µL, 0.034 mmol, 0.33 equiv.) was dissolved in 3–4 mL and added drop by drop to the reaction flask using syringe and under nitrogen atmosphere. Color of the reaction mixture was milky white and upon adding DIPEA and hexylamine, the reaction solvent turns yellow and the reaction mixture was clear without any solid crystals. After 5 hours, the reaction mixture was dried *in vacuo* **6** was separated by silica gel flash column chromatography using DCM with 5% (v/v) acetonitrile. **6** was not obtained as pure and it contains starting materials **2**, free 4-nitrophenol, which can be seen in **Figure S17** ^{1}H NMR spectrum. Molecule **6** was produced in 38% yield from proton ^{1}H NMR peak integration analysis.

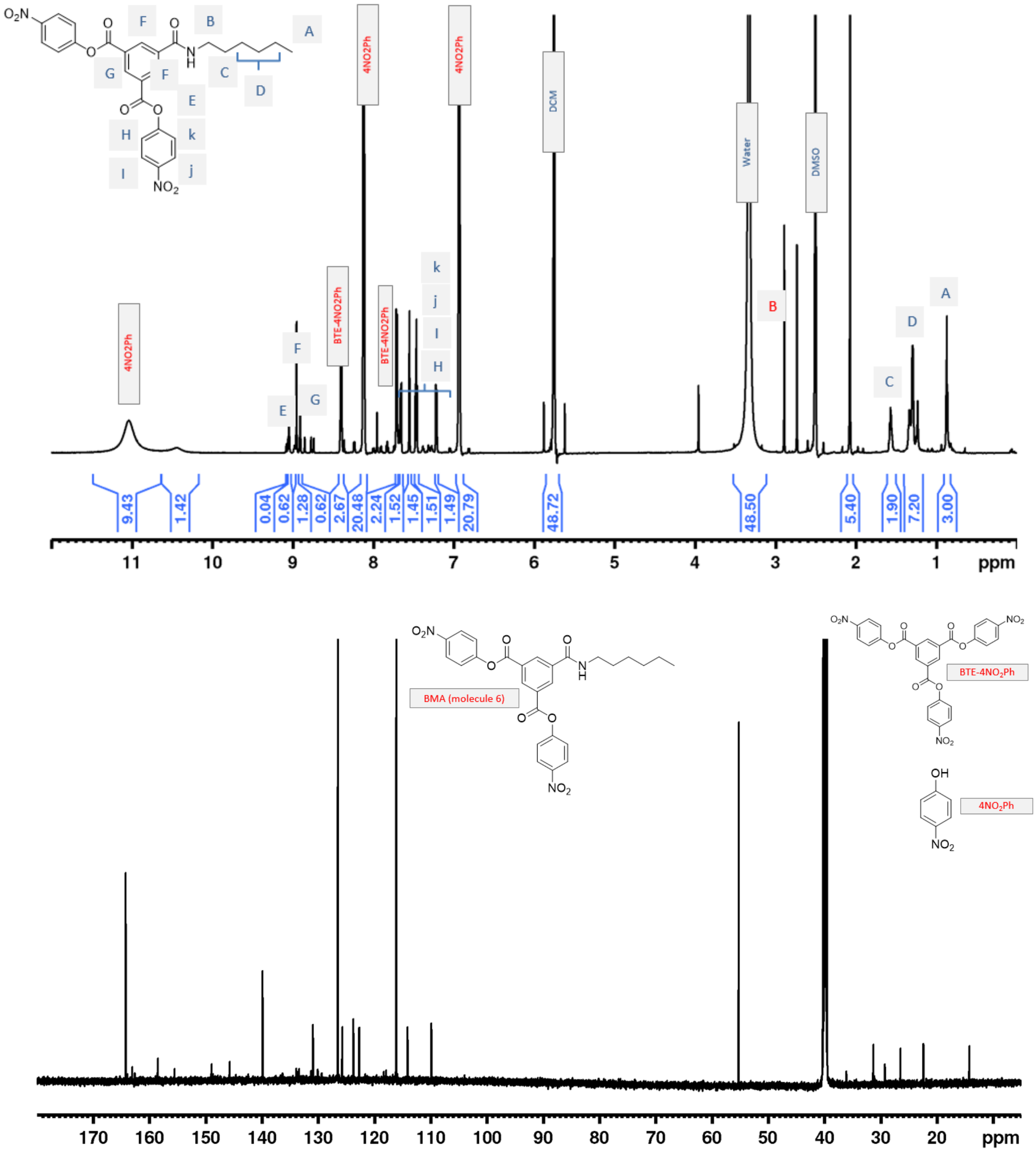

Figure S17: ^{1}H NMR (top) and ^{13}C NMR (bottom) of **6** (BMA-4NO_2Ph). It contains starting materials BTE-4NO_2Ph and free 4-nitrophenol. All eluted together in flash column chromatography separation.

Synthesis of molecule 8:

In dry round bottom flask, **2** (20 mg, 0.11 mmol, 1 equiv.) was dissolved in 5–6 mL anhydrous DMF under nitrogen atmosphere. DIPEA (18 µL, 0.11 mmol, 1 equiv.) and hexylamine (9.2 µL, 0.07 mmol, 0.66 equiv.) was dissolved in 5–6 mL of anhydrous DMF and added dropwise to the reaction flask under nitrogen atmosphere. After 5 hours, reaction solvent was dried *in vacuo*. DCM with 10% (v/v) acetonitrile solvent mixture was used to elute **8** on silica gel flash column chromatography. Molecule **8** was obtained (3 mg, 17% isolated yield). ^{1}H NMR (700 MHz, *d6*-DMSO) δ 8.86 (t, 2H), 8.70 (d, 2H), 8.66 (bs, 1H), 8.39 (d, 2H), 7.68 (d, 2H), 3.29 (t, 4H), 1.54 (p, 4H), 1.39 (m, 12H), 0.87 (t, 6H), ^{13}C NMR (176 MHz, d_6-DMSO) δ 164.54, 163.55, 155.67, 145.65, 135.93, 131.90, 131.00, 129.16, 125.71, 123.71, 31.29, 29.23, 26.44, 22.34, 14.21. Note, Carbon atom labelled as 1 in the ^{13}C spectrum shows up under DMSO peaks.

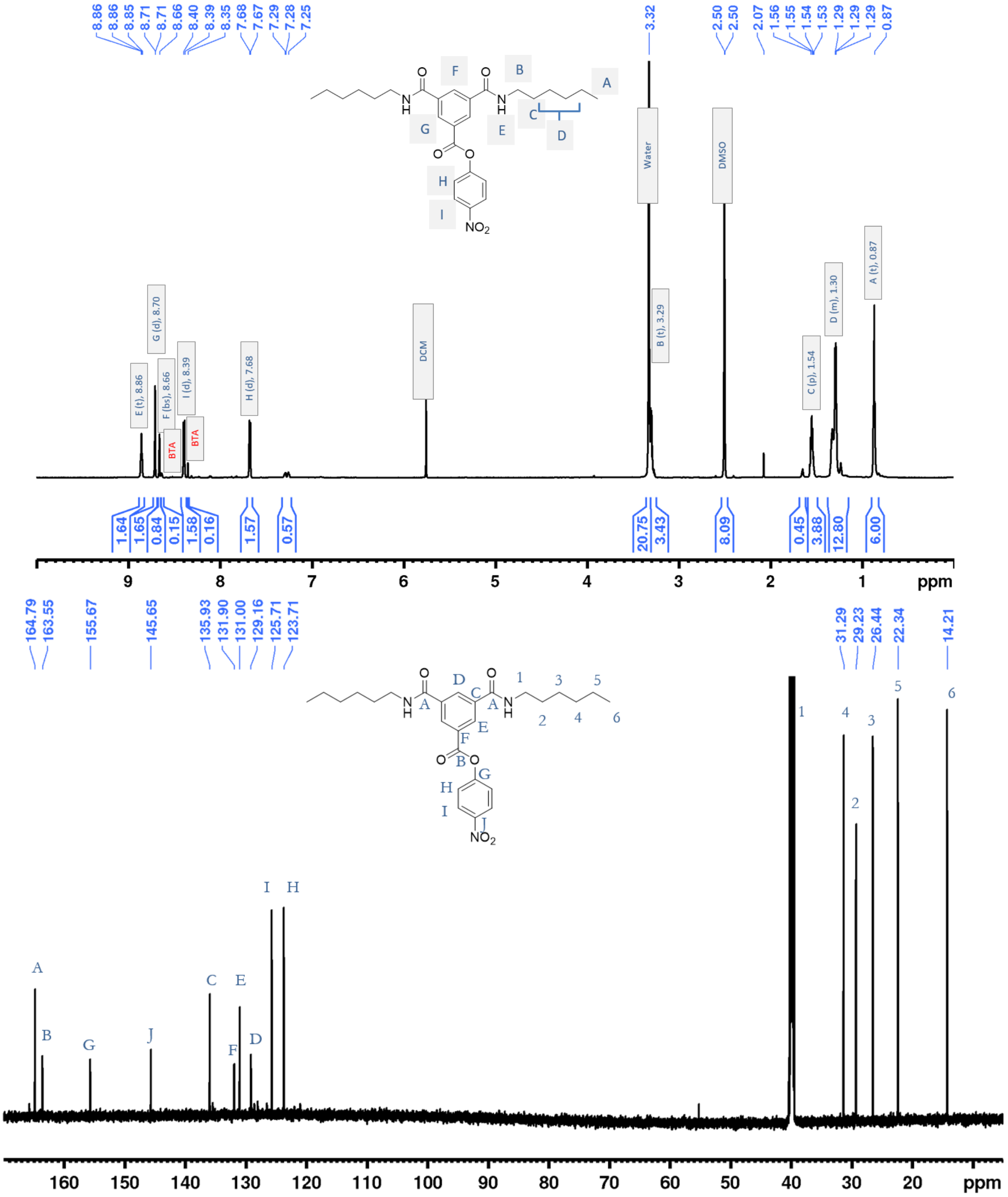

Figure S18: ^{1}H NMR of molecule **8** (top). Molecule **8** is around 95% pure and contains around 5% of trisubstituted hexylamine derivative labelled as BTA. ^{13}C NMR (bottom) of molecule **8** (bottom).

Desymmetrization of tris(perfluorophenyl) benzene-1,3,5-tricarboxylate (3):

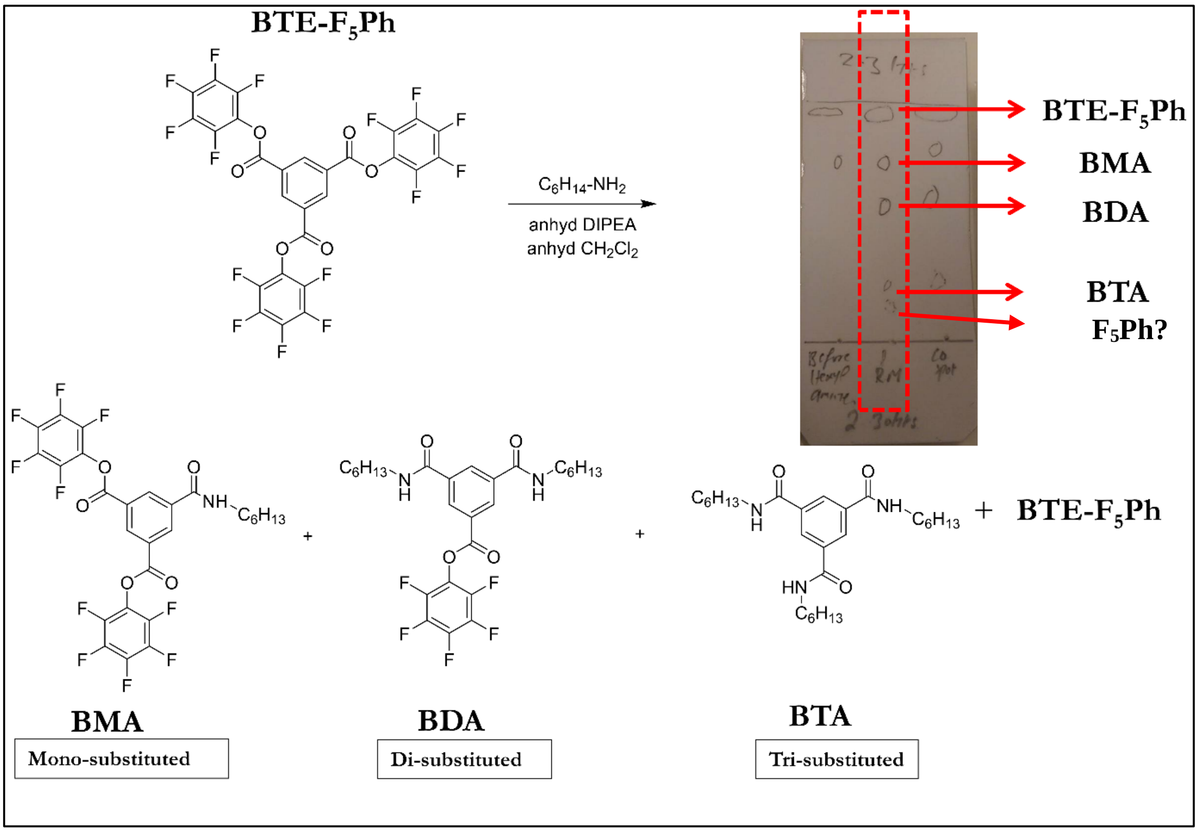

Figure S19: Reaction scheme of desymmetrization of molecule **3** (BTE-F_5PH)**.** Thin layer chromatography showed that there are total of five spots which indicates that there are total five molecules in reaction mixture.

Synthesis of molecule 9:

In the dried round bottom flask under nitrogen atmosphere molecule **3** (15 mg, 0.064 mmol, 1 equiv.) was dissolved in 3 mL of anhydrous DCM and the reaction glass flask was placed into a cold dry ice bath in acetone (-78 °C). DIPEA (14.8 μL, 0.084 mmol, 1.3 equiv.) dissolved in anhydrous DCM was added to the reaction flask. After this, solution of hexylamine (2.8 μL, 0.021 mmole , 0.33 equiv.) dissolved in 4 mL anhydrous DCM was added dropwise to the reaction flask under nitrogen atmosphere. The reaction solution was stirred for 1.5 hours at -78 °C. The reaction solution was concentrated *in vacuo* and purified by silica gel flash column chromatography. Monosubstituted derivative, **9**, was separated using dichloromethane as eluent and obtained as white solid (5.3 mg, 40% isolated yield). ^{1}H NMR (700 MHz, $CDCl_3$) δ 9.09 (t, 1H), 8.89 (d, 2H), 6.5 (t, 1H), 3.54 (q, 2H), 1.67 (p, 2H), 1.30-1.45 (m, 6H), 0.87 (t, 3H). ^{13}C NMR (176 MHz, $CDCl_3$) δ 164.6, 161.1, 137.1, 134.9, 134.6, 128.8, 40.8, 31.6, 29.6, 26.8, 22.7, 14.2. MS (MALDI-TOF) calcd. for $C_{27}H_{17}F_{10}NO_5$: 625.09, found: 626.12 $[M+H]^+$.

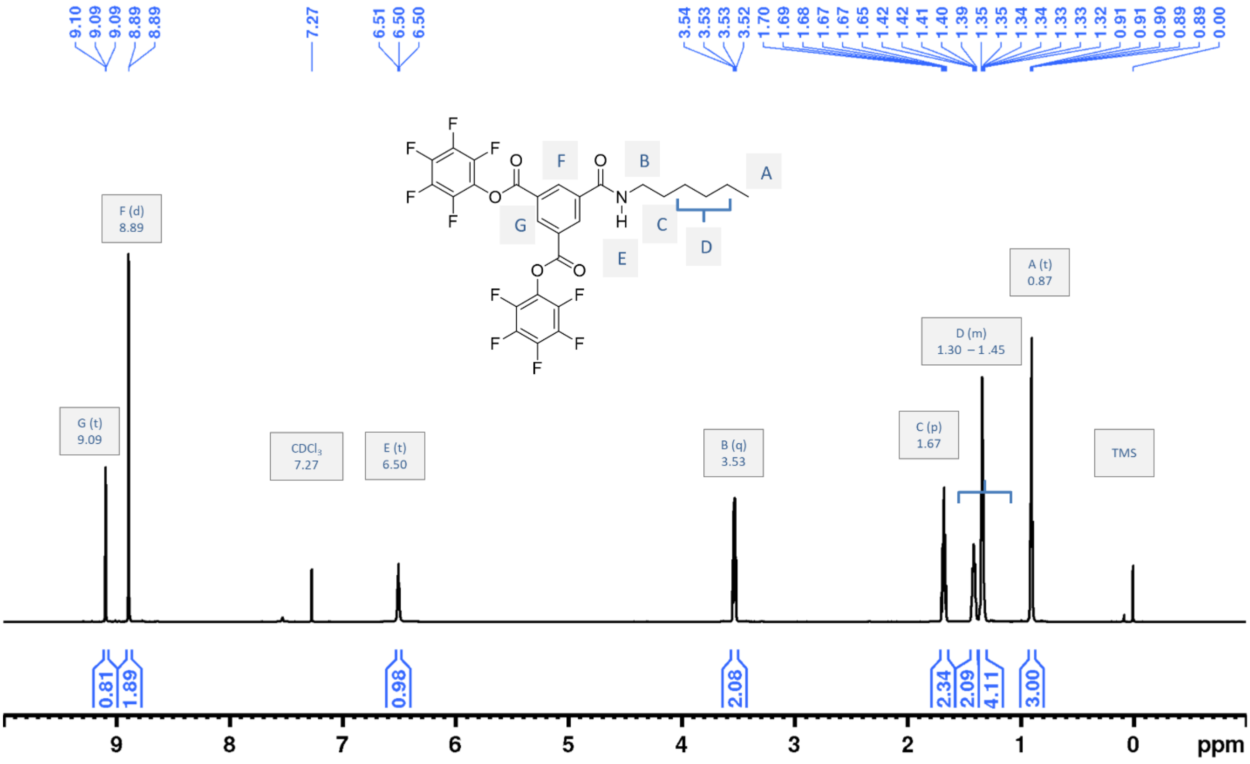

Figure S20: ^{1}H NMR ($CDCl_3$) of molecule **9**

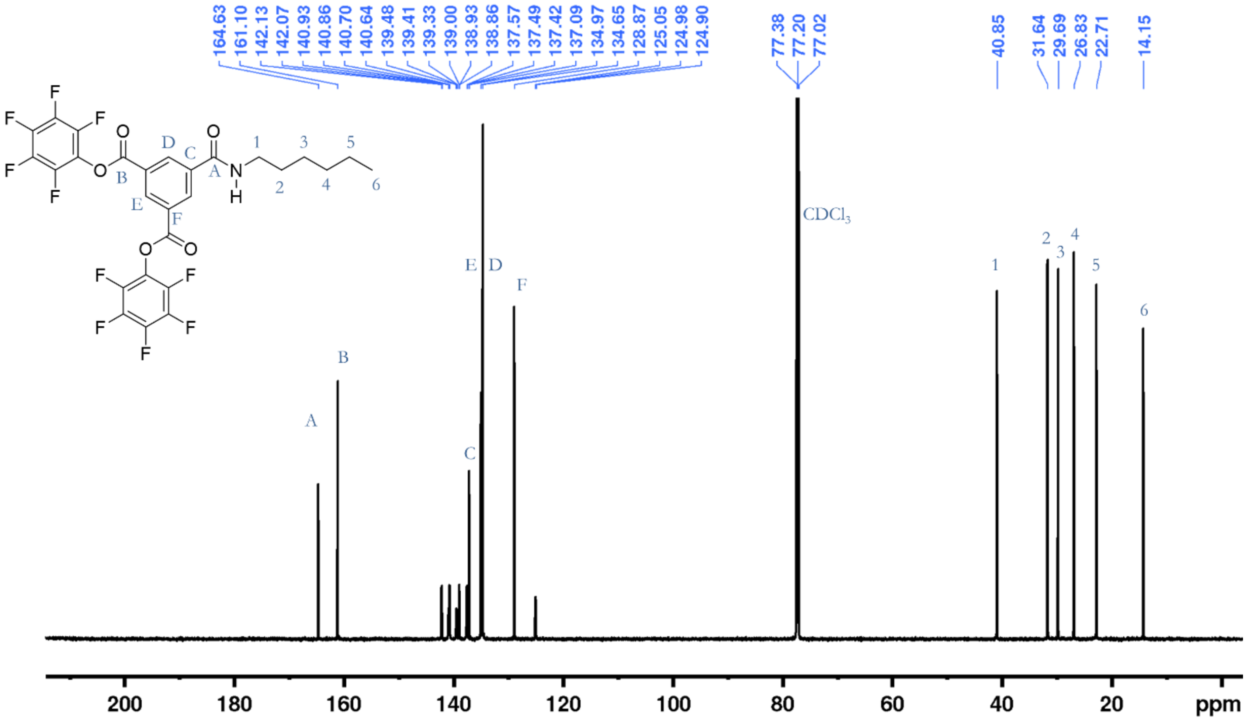

Figure S21: ^{13}C NMR ($CDCl_3$) molecule **9**

Synthesis of molecule 10:

Molecule **3** (15 mg, 0.064 mmol, 1 equiv.) was dissolved in anhydrous DCM (3 mL) under nitrogen atmosphere and cooled in a dry ice bath to -78°C. Solution of DIPEA (14.7 µL, 0.085 mmol, 1.33 equiv.) in anhydrous DCM (3 mL) was added dropwise to the reaction flask under nitrogen atmosphere. Subsequently, dry hexylamine (5.6 µL, 0.042 mmol, 0.66 equiv.) solution in anhydrous DCM (3 mL) was added dropwise to the reaction flask cooled to -78°C in about 15 minutes under nitrogen atmosphere. The reaction solution was stirred for 1.5 hours at -78°C and concentrated *in vacuo*. The reaction mixture was purified using silica gel flash column chromatography and molecule **10** was separated using eluent 95% by volume DCM and 5% by volume acetonitrile. Molecule **10** was obtained as white solid in 48 % yield (5.5 mg). ^{1}H NMR (700 MHz, $CDCl_3$) δ 8.66 (d, 2H), 8.53 (t, 1H), 6.59 (t, 2H), 3.47 (q, 4H), 1.63 (p, 4H), 1.32 (m, 12H), 0.88 (t, 6H).^{13}C NMR (176 MHz, $CDCl_3$) δ 165.3, 161.7, 136.3, 131.5, 131.4, 128.0, 40.7, 31.6, 29.6, 26.8, 22.7, 14.2. MS (MALDI-TOF), calcd. for $C_{27}H_{31}F_5N_2O_4$: 542.2, found: 543.2 $[M+H]^+$.

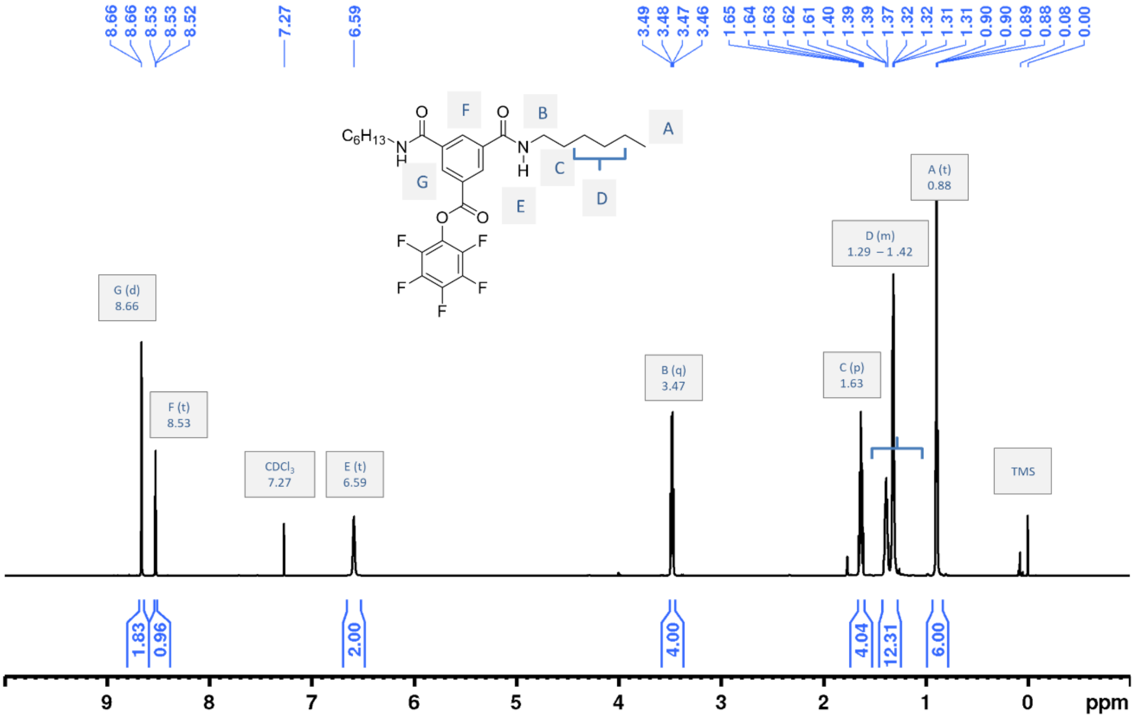

Figure S22: ^{1}H NMR ($CDCl_3$) of molecule **10**

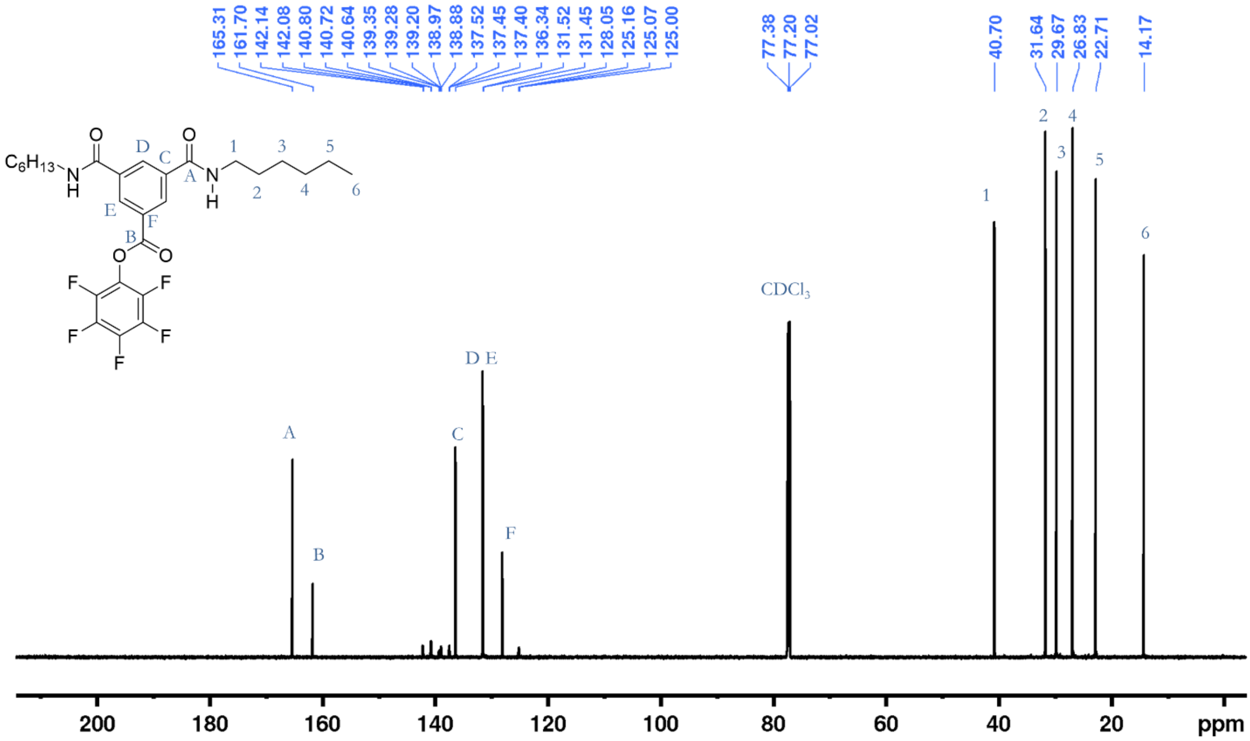

Figure S23: ^{13}C NMR ($CDCl_3$) of molecule **10**

Synthesis of molecule 11:

In dry round bottom flask, **3** (50 mg, 0.21 mmol, 1 equiv.) was dissolved in 3 mL of anhydrous DCM under nitrogen atmosphere. The temperature of the solution was maintained to 4°C using cold ice bath. DIPEA solution (49.1 µL, 0.28 mmol, 1.33 equiv.) was dissolved in anhydrous 3 mL anhydrous DCM and added to the reaction flask. Reaction solvent color turns yellow. Subsequently, solution of dodecylamine (13.1 µL, 0.07 mmol, 0.33 equiv.) in 3 mL anhydrous DCM was added dropwise to the reaction flask. The reaction was allowed to stir at 4°C and after 2 hours reaction solvent was removed under reduced pressure and ^{1}H NMR of the reaction mixture was taken and **11** was produced in 39% yield from proton ^{1}H NMR analysis. Using DCM solvent on silica gel flash column chromatograph, **11** was separated. ^{1}H NMR (700 MHz, $CDCl_3$) δ 9.1 (1H, t), 8.88 (2H, d), 6.32 (1H, t), 3.53 (2H, q), 1.68 (2H, m), 1.45–1.26 (18H, m), 0.88 (3H, t). ^{13}C NMR (176 MHz, $CDCl_3$) δ 164.5, 161.1, 137.1, 134.9, 134.6, 128.8, 40.8, 32.08, 29.82, 29.79, 29.76, 29.74, 29.71, 29.51, 29.48, 27.18, 22.85, 14.26. MS (MALDI-TOF) calcd. for $C_{33}H_{29}F_{10}NO_5$: 709.9, observed:710.2 [M+H]$^+$.

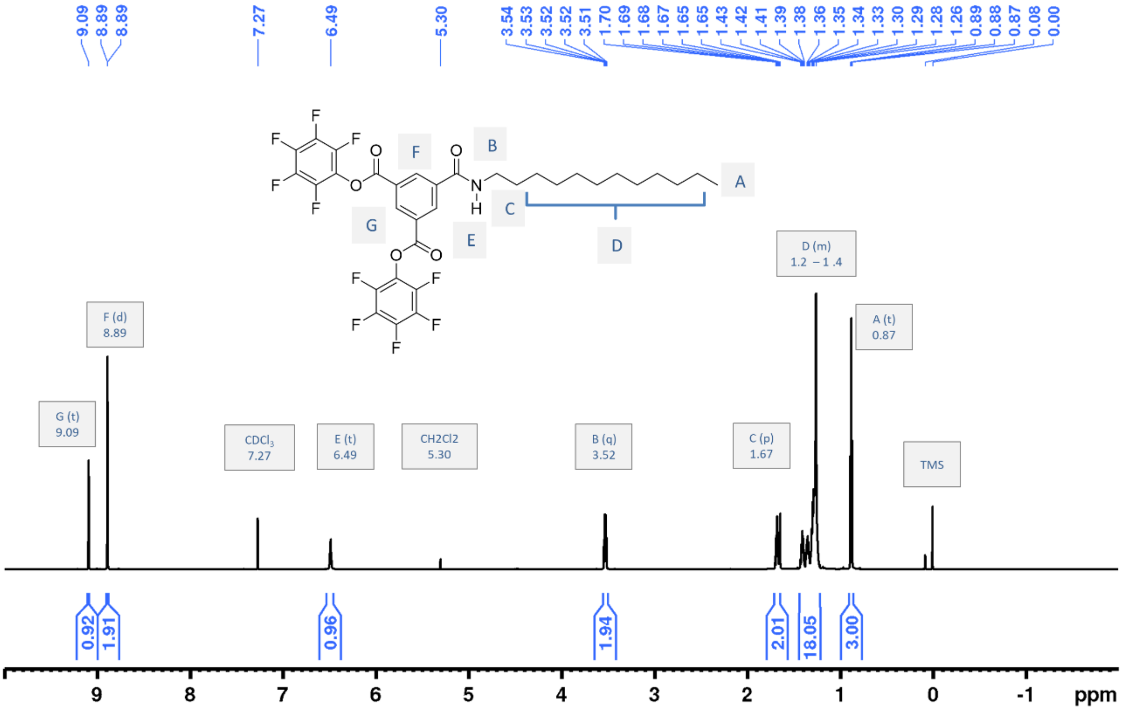

Figure S24: ^{1}H NMR ($CDCl_3$) of molecule **11**

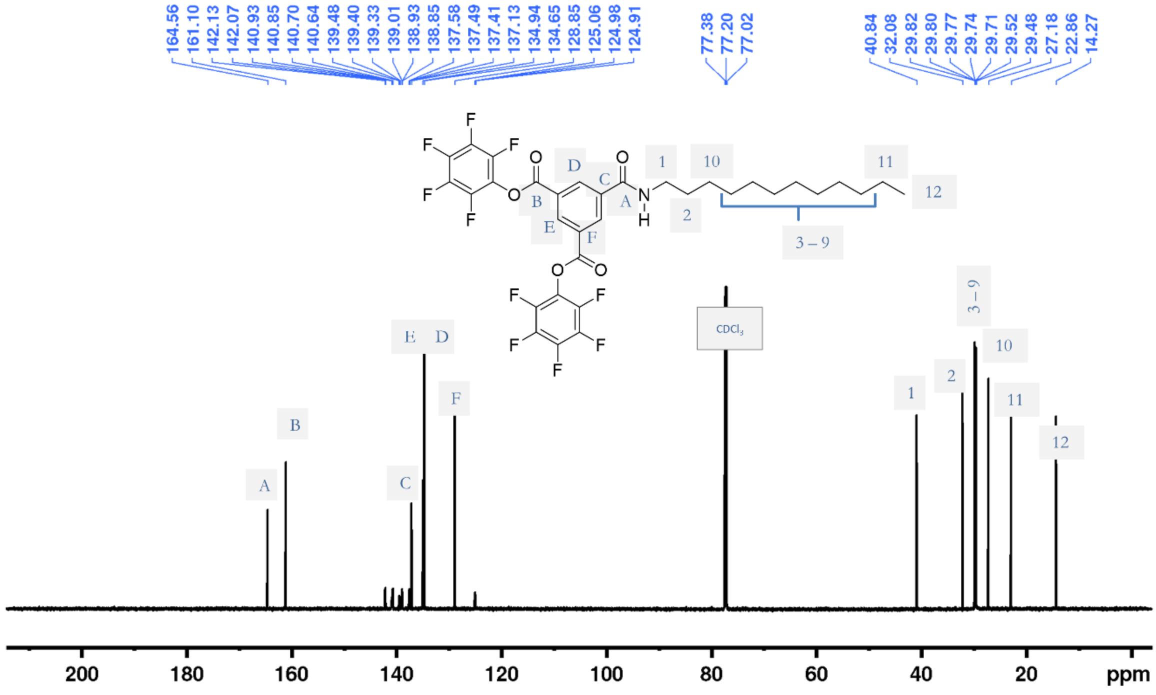

Figure S25: ^{13}C NMR ($CDCl_3$) of molecule **11.**

Synthesis of molecule 12:

In a dry round bottom flask, **3** (1000 mg, 4.2 mmol, 1 equiv.) was dissolved in 50 mL of anhydrous DCM under nitrogen atmosphere. The reaction flask was set into ice-cold bath at 4 °C. DIPEA (863 µL, 5.0 mmol, 1.2 equiv.) solution in anhydrous DCM was added dropwise to the reaction flask at 4 °C using a syringe. Once the reaction solution temperature was maintained at 4 °C, dodecylamine (523 mg, 2.8 mmol, 0.67 equiv.) in 25 mL anhydrous DCM was added dropwise to the reaction flask under nitrogen atmosphere and over a time interval of 10–15 minutes. The reaction was stirred for 1.5 hours at 4 °C. The reaction mixture was vacuum dried under reduced pressure and **12** was obtained using DCM with 5% (v/v) acetonitrile as eluent in 49% isolated yield (486 mg) as white powder. ^{1}H NMR (700 MHz, $CDCl_3$) δ 8.67 (2H, d), 8.52 (1H, t), 6.49 (2H, t), 3.49 (4H, q), 1.64 (4H, m), 1.4–1.2 (36H, m), 0.88 (6H, t). ^{13}C NMR (176 MHz, $CDCl_3$) δ 165.2, 161.7, 136.3, 131.5, 131.4, 128.0, 40.7, 32.08, 29.82, 29.80, 29.77, 29.72, 29.71, 29.52, 29.49, 27.18, 22.86, 14.28. MS (MALDI-TOF), calcd. for $C_{39}H_{55}F_5N_2O_4$: 710.4, found : 711.4 $[M+H]^+$.

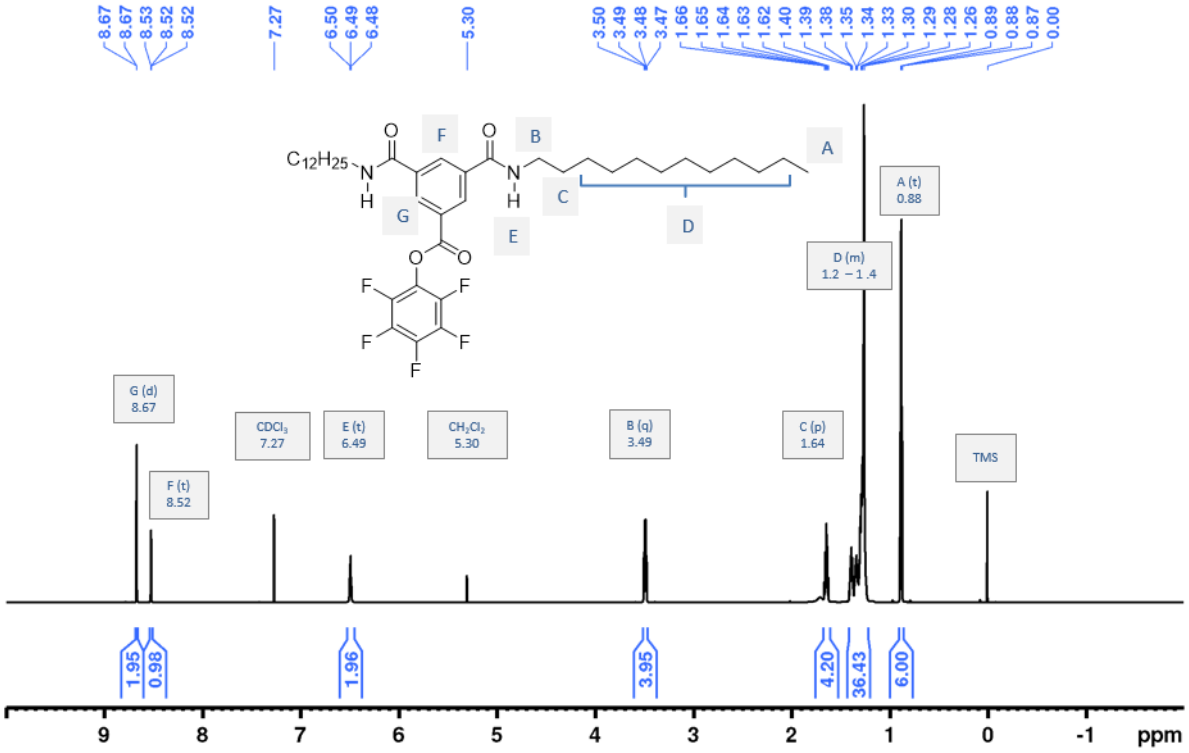

Figure S26: ^{1}H NMR ($CDCl_3$) of molecule **12**

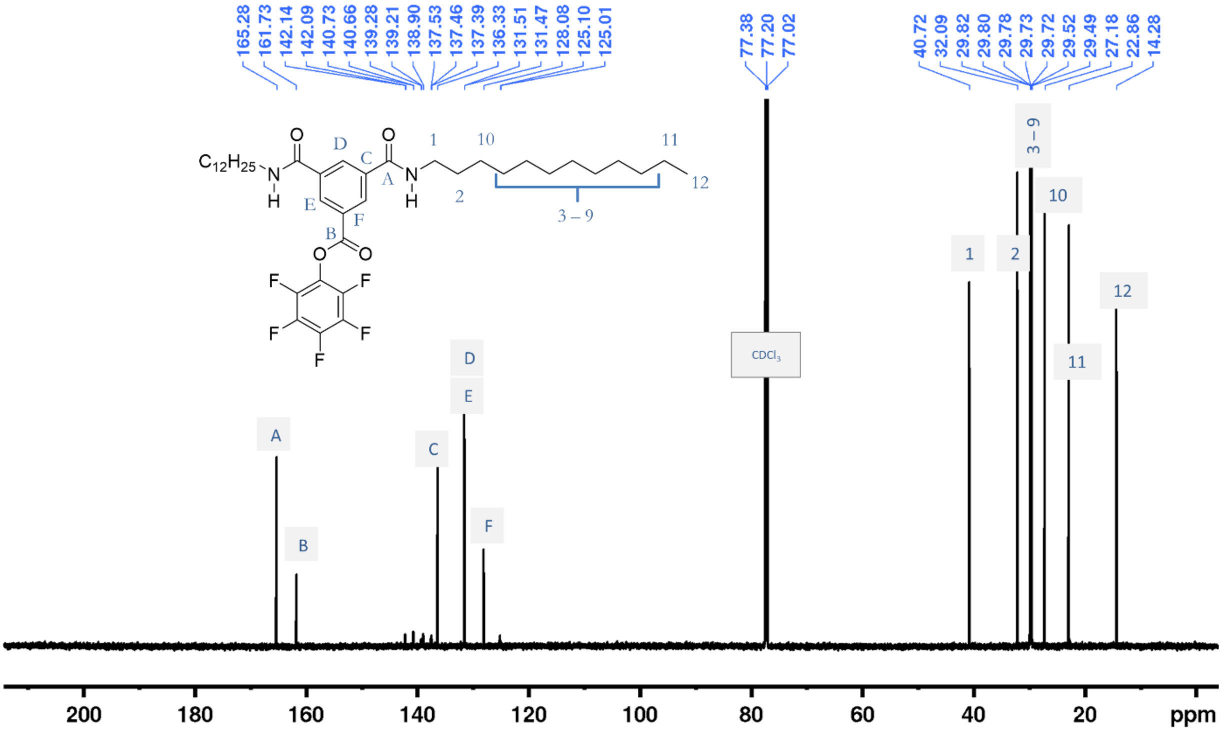

Figure S27: ^{13}C NMR ($CDCl_3$) of molecule **12**

Table S6: Table showing molecules formed in desymetrization reaction. BMA and BDA molecule formation was found to be maximum at equimolar concentration of aliphatic amines. % of molecules formed reported in table are derived from ^{1}H NMR peaks integration analysis.

	Substituent (R)				
	Hexyl (C_6)		Dodecyl (C_{12})		Norbornene (Nb)
**Reaction conditions**	**4°C**	**-78°C**	**4°C**	**-78°C**	**4°C**
% **BMA** (0.5 and 1 mole equiv.)	34% and 48%	32% and 49%	32% and 39%	26% and 42%	50%
% **BDA** (1 and 2 mole equiv.)	14% and 53%	26% and 65%	28.7% and 56%	26% and 57%	70%

Desymmetrization of tris(perfluorophenyl) benzene-1,3,5-tricarboxylate (3) using 5Nb-2MA:

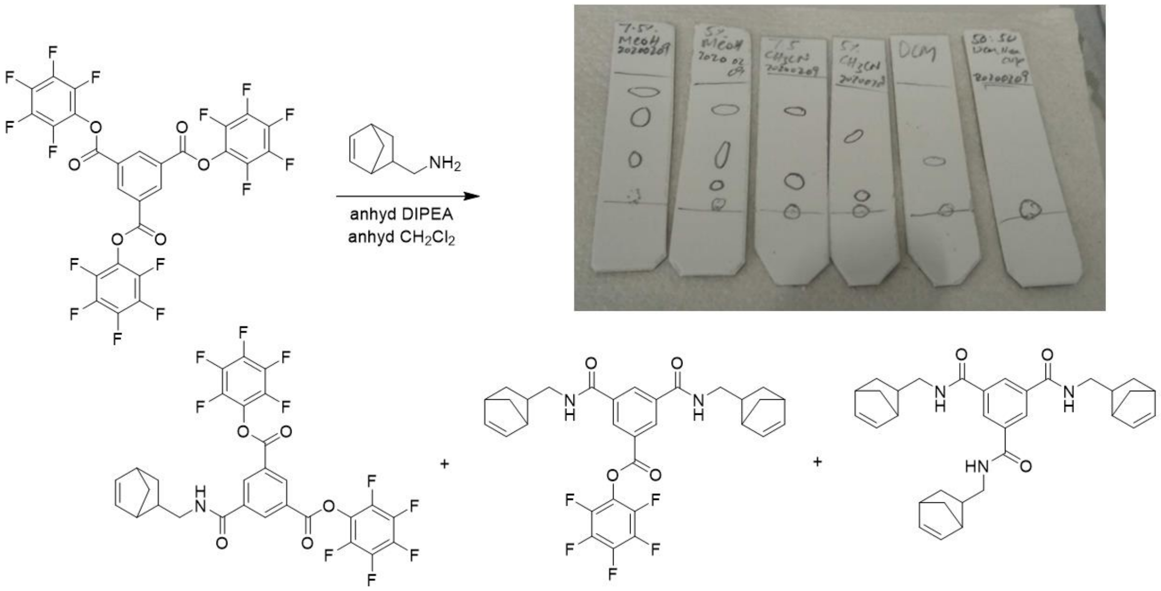

Figure S28: Desymmetrization of molecule **3** using 5-Norbornene-2-methylamine to create molecule **13** (monosubstituted) and **14** (disubstituted) derivatives.

Synthesis of molecule 13:

Under nitrogen atmosphere and in a dry round bottom flask, **3** (20 mg, 0.084 mmol, 1 equiv.) was dissolved in 3 mL anhydrous DCM. Reaction flask was placed into ice cold bath (4 °C) and then DIPEA (9.84 µL, 0.06 mmol, 0.67 equiv.) dissolved in anhydrous DCM was added into the reaction flask. Later, 5Nb-2MA (3.62 µL, 0.03 mmol, 0.33 equiv.), dissolved in anhydorus DCM, was added to the reaction flask drop by drop over 5–10 minutes using syringe under nitrogen atmosphere. After addition, the reaction solution was allowed to stir for 2 hours and formation of **13** was checked by thin layer chromatogrpahy shown in **Figure S28**. The excess of DCM was removed *in vacuo* and **13** was separated using silica gel flash chromatography using eluent DCM as white powder in 40% isolated yield (7.2 mg) as white powder. ^{1}H NMR and ^{13}C NMR shown below. ^{1}H NMR (700 MHz, $CDCl_3$) δ 9.10-9.09 (m, 1H, $H_{A\text{-}endo\text{-}exo}$), 8.9 (d, 2H, $H_{B\text{-}exo}$), 8.87 (d, 2H, $H_{B\text{-}endo}$), 6.5 (t, 1H, $H_{C\text{-}exo}$), 6.4 (t, 1H, $H_{C\text{-}endo}$), 6.24(dd, 1H, H_{5endo}), 6.1 (m, 2H, $H_{5,6exo}$), 6.04 (dd, 1H,

H_{6endo}), 3.6(m, 2H, H_8), 3.5(m, 2H, H_8), 3.25(m, 2H, H_8), 2.91 (s, 1H, H_1), 2.87 (s, 1H, H_4), 2.7(m, 2H, H_2), 2.43(m, 2H, H_2), 2.35(t, 2H, H_2), 1.94 (m, 2H, H_3), 1.50 (dd, 2H, H_7), 1.43 (bs, 2H, H_7), 1.41-1.33 (m, 2H, H_7), 1.31 (s, 2H, H_7), 1.29(s, 2H, H_7), 1.26 (m, 2H, H_3), 0.69 (ddd, 2H, H_3). ^{13}C NMR (176 MHz, $CDCl_3$) δ 164.4, 161.07, 142.08, 140.8, 140.5, 139.3, 138.8, 138.2, 137.45, 137.2, 137.1, 137.0, 136.2, 134.95, 134.91, 134.65, 134.59, 132.07, 128.8, 124.9, 45.9, 45.2, 44.7, 44.5, 42.6, 41.94, 39.3, 39.00, 31.16, 30.32. MS (MALDI-TOF), calcd. for $C_{29}H_{15}F_{10}NO_5$: 647.08, found : 647.94 $[M+H]^+$.

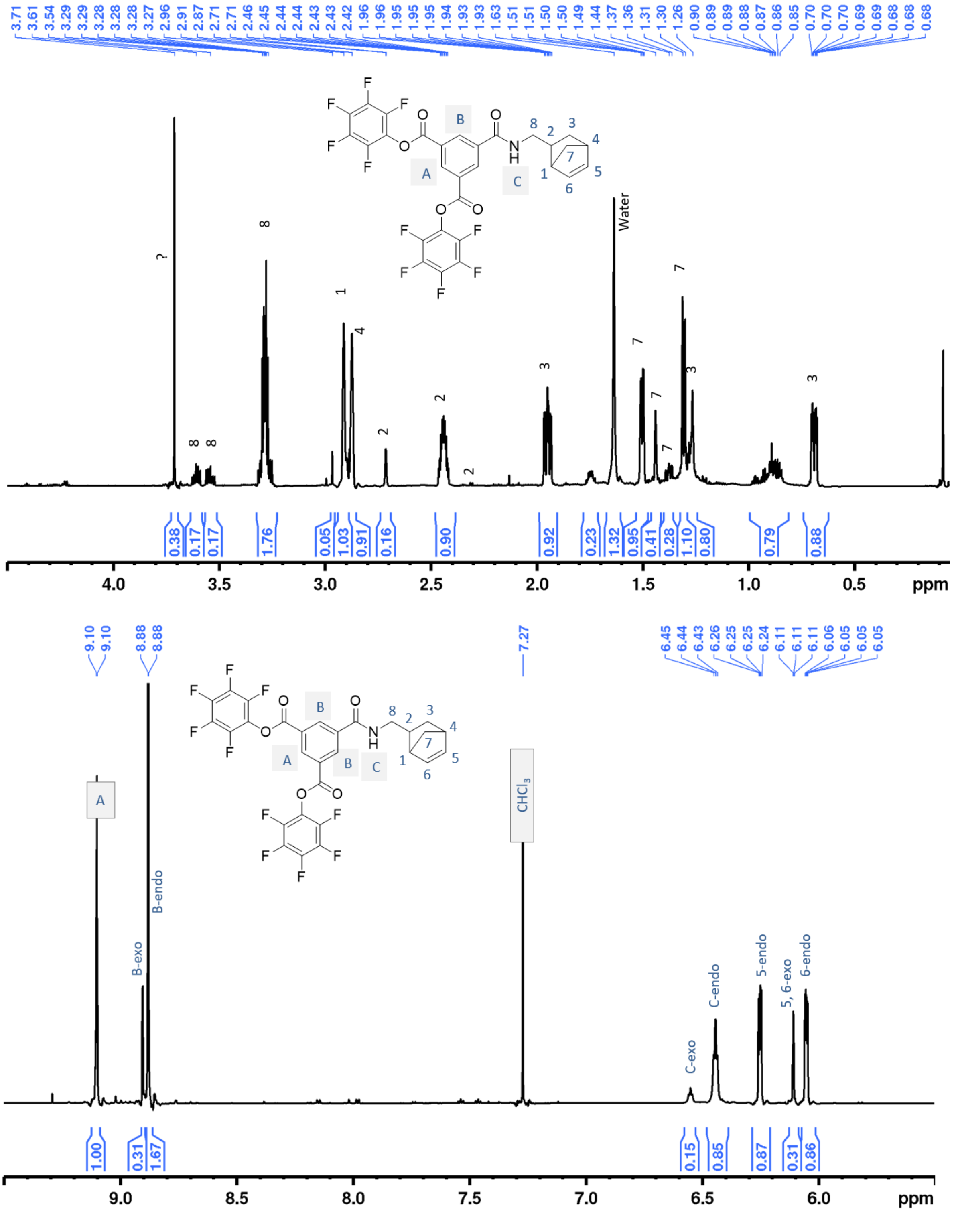

Figure S29: ^{1}H NMR **molecule 13**. Top spectrum from 0–5 ppm and bottom spectrum from 5.5–9 ppm.

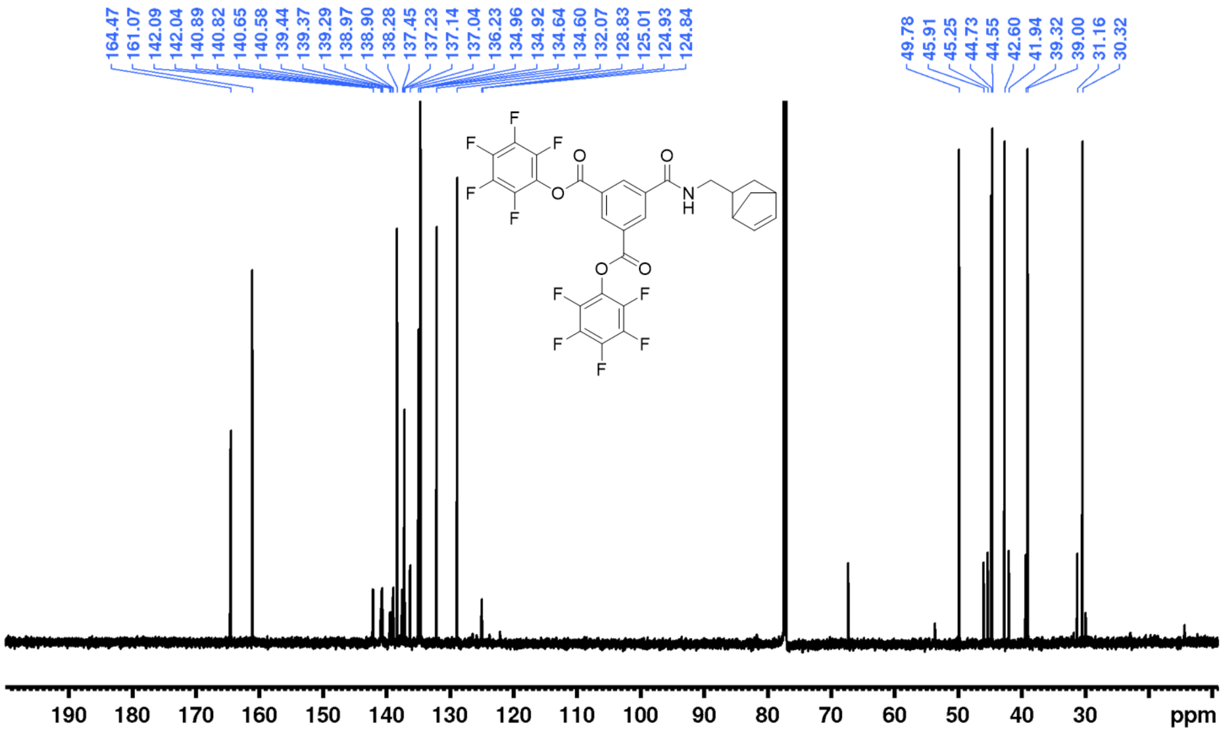

Figure S30: ^{13}C NMR **molecule 13**.

Synthesis of molecule 14:

A dry round bottom flask was loaded with molecule **3** (500 mg, 2.11 mmol, 1 equiv.) under nitrogen atmosphere and dissolved in 40 mL anhydrous DCM. Subsequently solution of anhydrous DIPEA (182 µL, 2.11 mmol, 1 equiv.) in 2 mL anhydrous DCM was added into the reaction flask. Recation flask was set inot ice bath (4 ºC). Solution of 5Nb-2MA (182 µL, 1.41 mmol, 0.67 equiv.) in DCM (25 mL) was added drop wise to the reaction flask in roughly 10 minutes under nitrogen atmosphere. The reaction was stirred for 2.5 hours at 4 ºC and reaction mixture was vacuum dried to remove excess solvent. Nb-BDA was separated running flash column chromatography on silica gel using eluent DCM/acetonitrile (92.5/7.5) by volume. Nb-BDA was obtained as a white powder (202 mg) in 49% isolated yield and 52% yield based on recovered starting material. ^{1}H NMR (700 MHz, $CDCl_3$) δ 8.71-8.69 (m, 2H, $H_{Aexo\text{-}exo,\ endo\text{-}exo}$), 8.68-8.65(m, 2H, $H_{Aexo\text{-}end,\ endo\text{-}endo}$), 8.55 (t, 1H, $H_{Bexo\text{-}exo}$), 8.53 (t, 1H, $H_{Bendo\text{-}exo}$), 8.51(t, 1H, $H_{Bendo\text{-}endo}$), 6.5 (t, 1H, H_C), 6.4 (t, H, H_C) 6.24(dd, 1H, $H_{5endo\text{-}endo,\ exo\text{-}exo}$), 6.1 (t, 2H, $H_{5,6\ endo\text{-}exo}$), 6.04 (dd, H, $H_{6endo\text{-}endo,\ exo\text{-}exo}$), 3.58(m, 2H, H_8), 3.51(m, 2H, H_8), 3.25(m, 2H, H_8), 2.90 (bs, 1H, H_1), 2.86 (bs, 1H, H_4), 2.7(m, 2H, H_2), 2.41(m, 2H, H_2), 2.31(t, 2H, H_2), 1.94 (m, 2H, H_3), 1.49 (dd, 2H, H_7), 1.43 (bs, 2H, H_7), 1.41-1.33 (m, 2H, H_7), 1.30 (bs, 2H, H_7), 1.29(bs, 2H, H_7), 1.26 (m, 2H, H_3), 0.67 (ddd, 2H, H_3). ^{13}C NMR (176 MHz, $CDCl_3$) δ 165.13, 161.72, 142.12, 140.63, 139.28, 138.87, 138.80, 138.22, 137.45, 137.19, 136.38, 136.29, 132.11, 131.5, 131.42, 128.12, 125.06, 49.7, 45.8, 44.6, 44.5, 42.6, 41.9, 39.2, 39.0, 31.1, 30.3. MS (MALDI-TOF), calcd. for $C_{31}H_{27}F_5N_2O_4$: 586.19, found : 587.13 $[M+H]^+$.

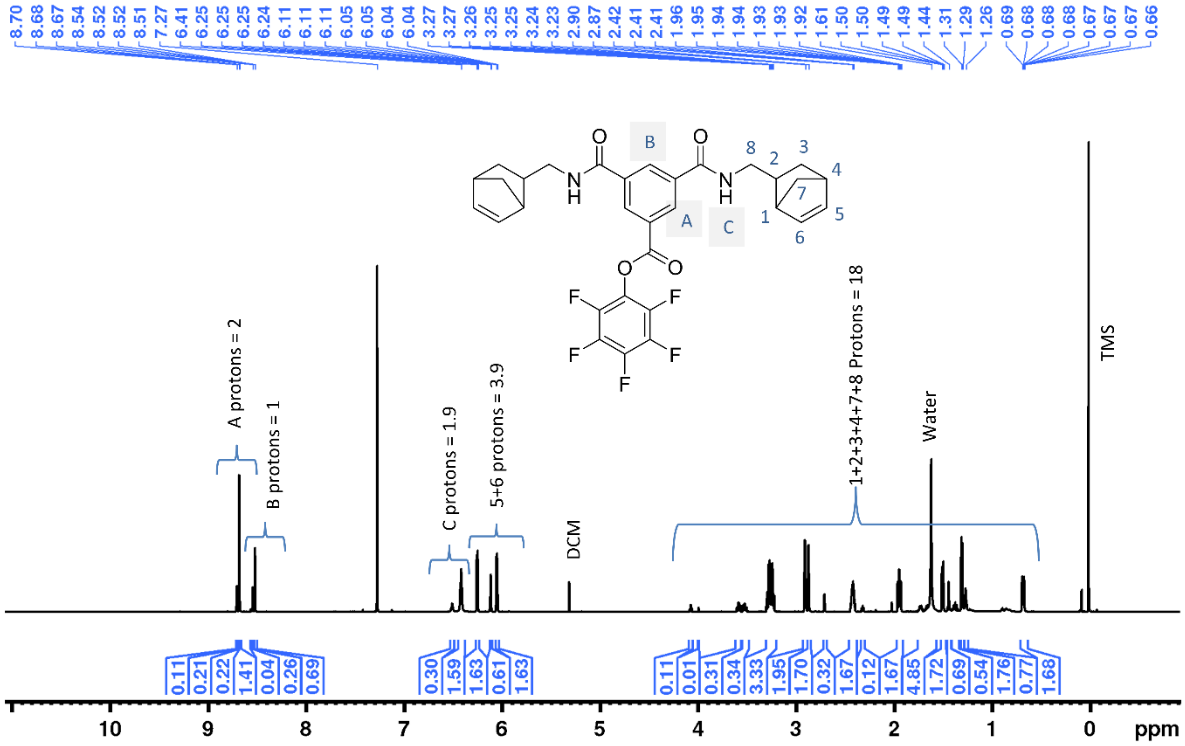

Figure S31: ^{1}H NMR ($CDCl_3$) molecule **14**.

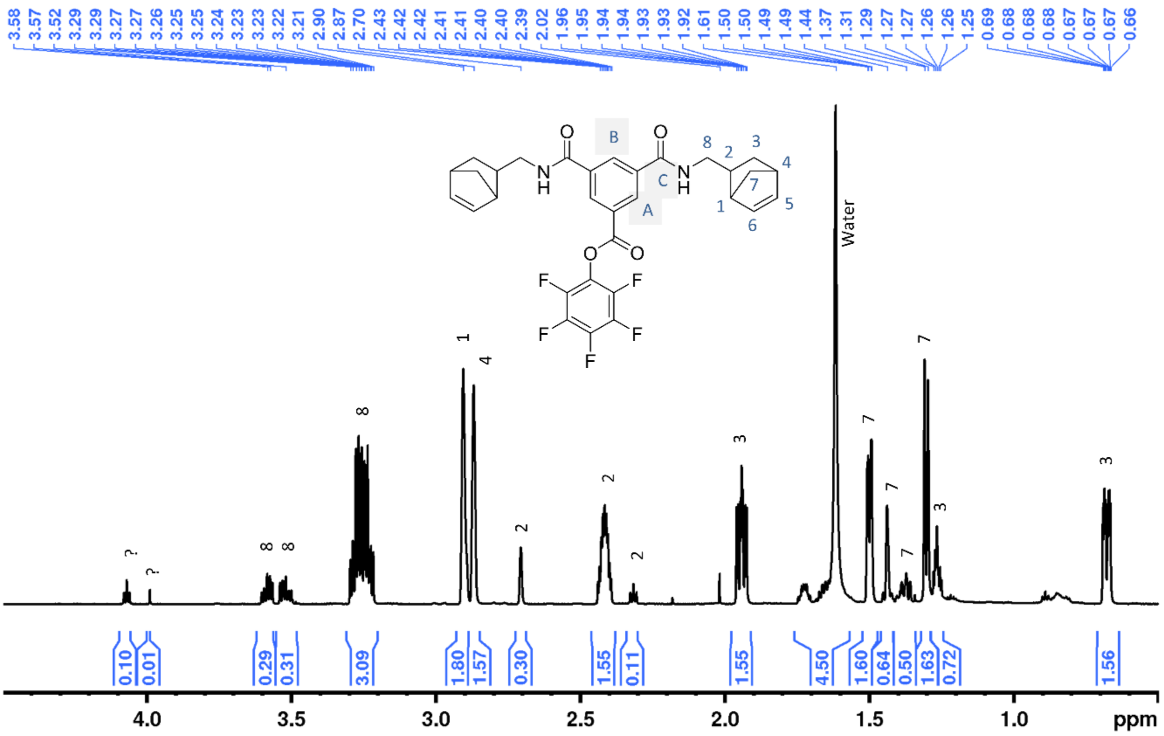

Figure S31A: ^{1}H NMR ($CDCl_3$) **14** molecule, zoomed in from 0.0 ppm to 4.5 ppm.

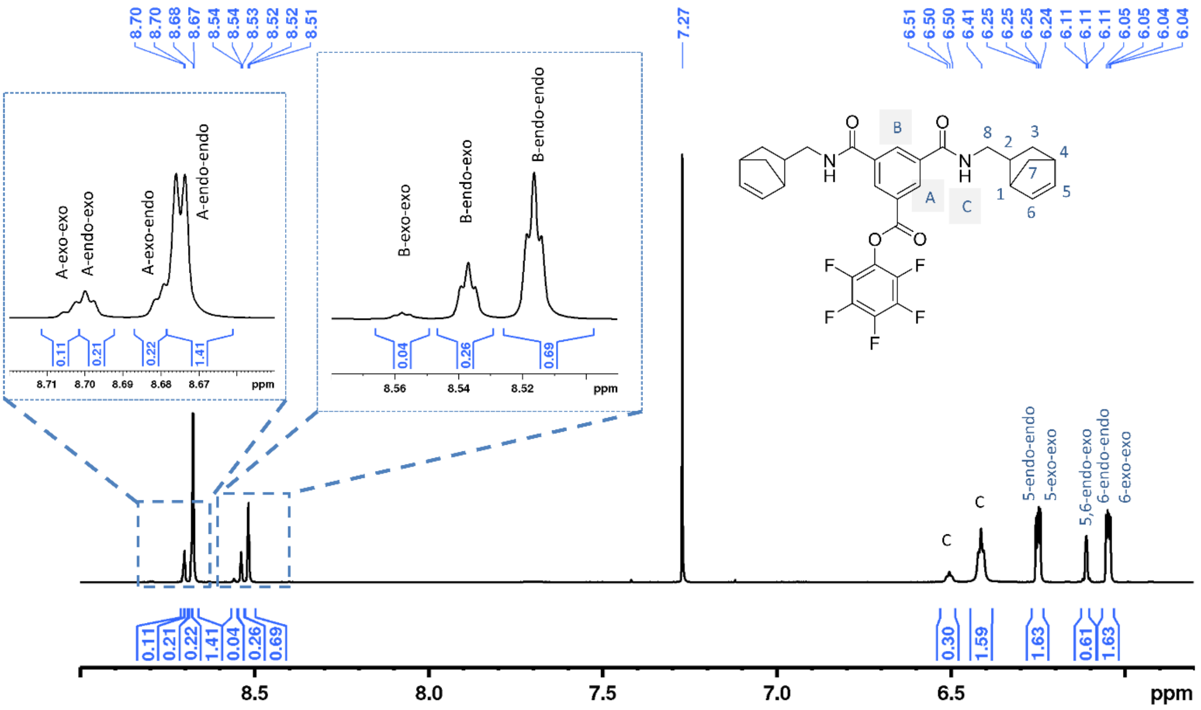

Figure S31B: ^{1}H NMR ($CDCl_3$) **14**, zoomed in from 5.8 ppm to 9.0 ppm.

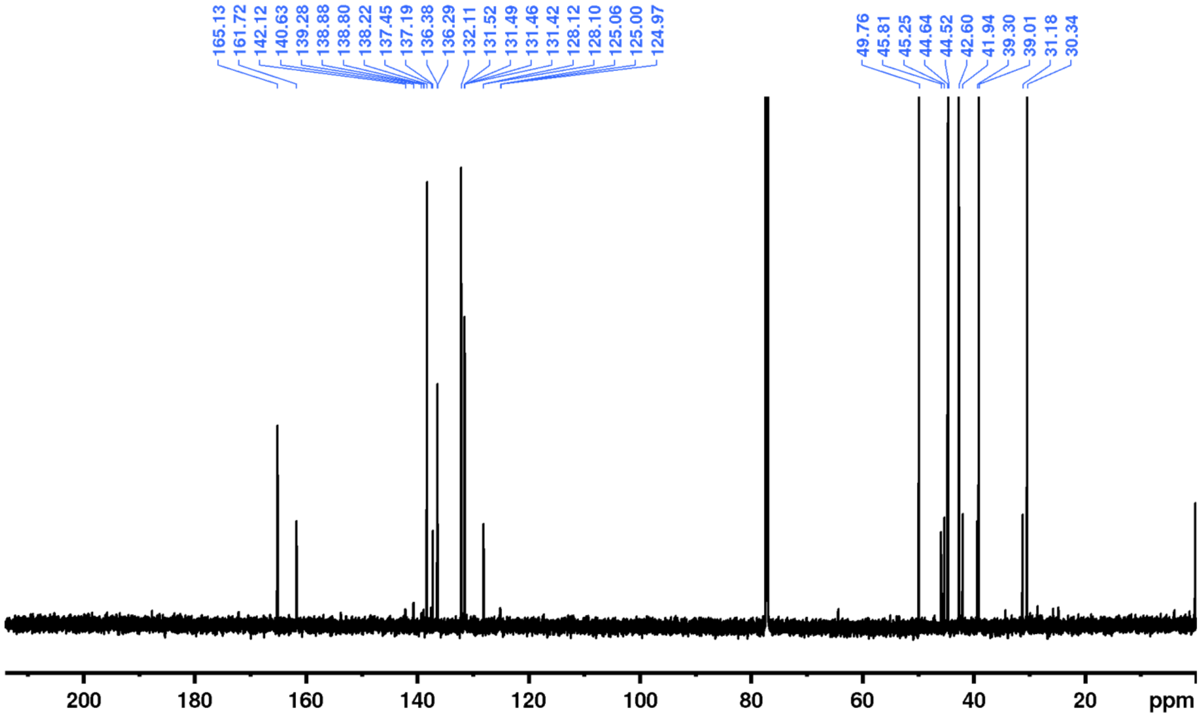

Figure S32: ^{13}C NMR ($CDCl_3$) **14.**

Desymmetrization using 6-amino-1-hexanol:

(A)

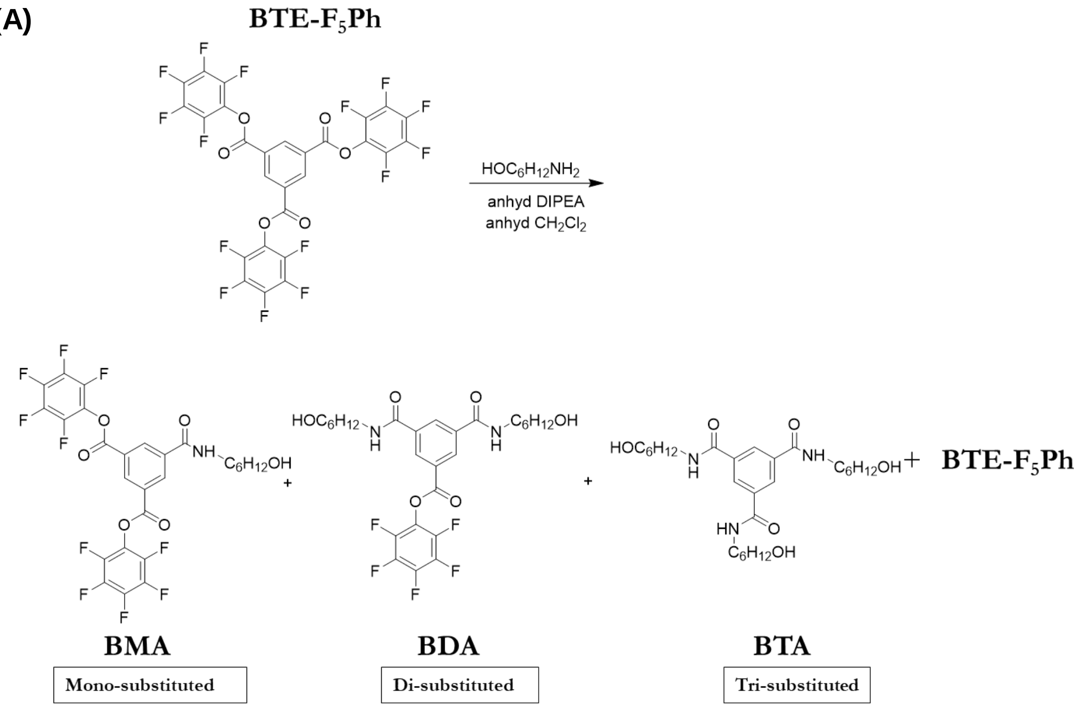

(B)

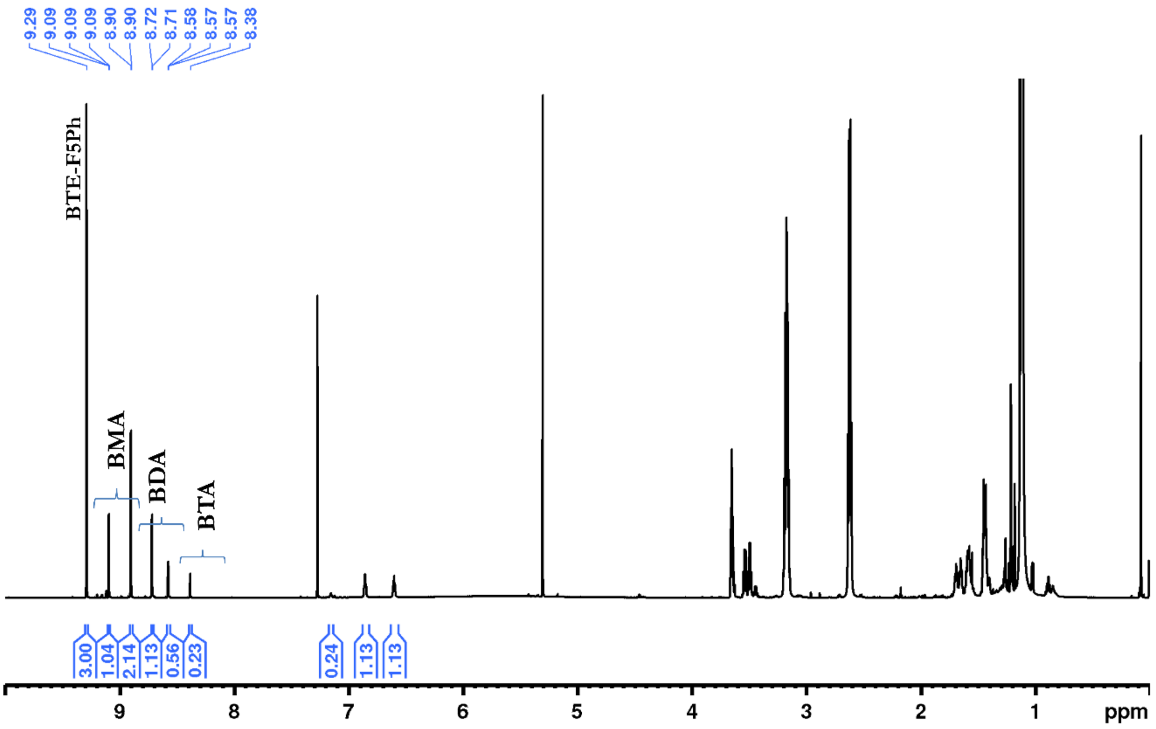

Figure S33: A) Reaction scheme for desymmetrization of molecule **3** using hexylamine alcohol. B) ^{1}H NMR ($CDCl_3$) of reaction mixture. Integration peak analysis indicates that there are total 4 molecules in reaction mixture which based on their peak ppm and peak integration analysis are mono-substituted, di-substituted and tri-substituted and leftover starting molecule **3** (BTE-F_5Ph).

Computational Details

General

Input files and molecular geometries were generated using Gaussview. Low energy structures were optimized in Gaussian 09W employing the B3LYP functional[1,2,3] up to the 6-311+G(d) basis set[4,5]; in all cases, no negative frequencies were found in the optimized geometries. Atomic charges were calculated via the NBO partitioning method[6] which also allows for the calculation of orbital occupancies. Electrophilicity values (ω)[7] were calculated according to the relationship $\omega = \mu^2/2\eta$, where μ is the electronic chemical potential and η is the global hardness. The values of μ and η can be approximated, applying Koopman's theorem[8], by $\mu = \frac{\epsilon HOMO + \epsilon LUMO}{2}$ and $\eta = \epsilon LUMO - \epsilon HOMO$. Electrostatic potential at the nuclei (EPN) (e.g., VC) were obtained using the Prop key word on geometry-minimized structures.

Optimized Structures

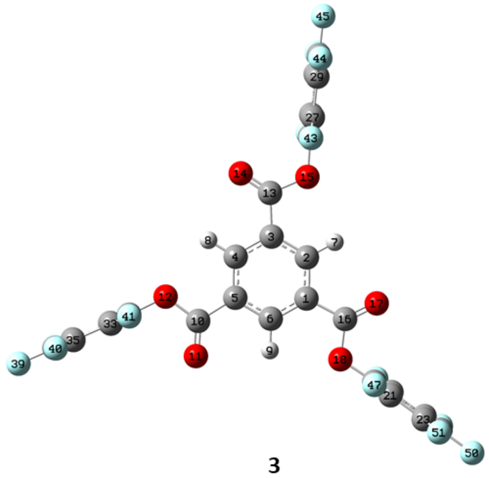

3

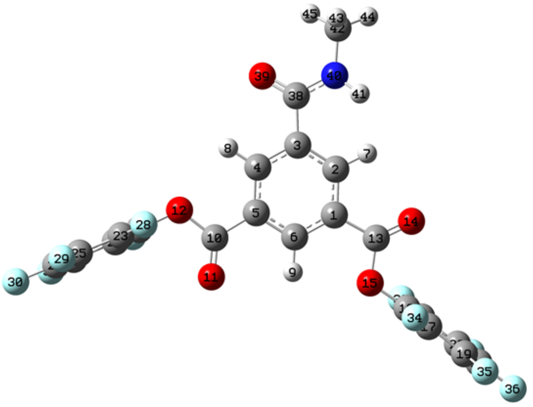

9a

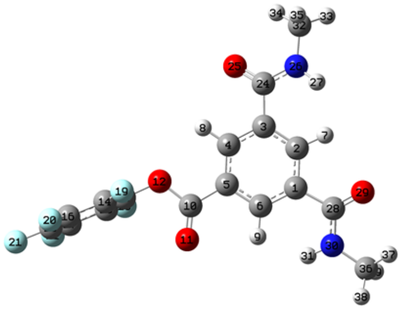

10a

Coordinates of optimized structures

3 (HF=-2980.2838139)

**C**	**0.95543**	**-1.01527**	**-0.01256**
**C**	**1.35568**	**0.32403**	**-0.0086**
**C**	**0.39746**	**1.33681**	**-0.01153**
**C**	**-0.96247**	**1.01381**	**-0.01704**
**C**	**-1.36048**	**-0.32247**	**-0.02078**
**C**	**-0.40071**	**-1.33879**	**-0.0191**
**H**	**2.41141**	**0.55973**	**-0.00268**
**H**	**-1.69437**	**1.81038**	**-0.01748**
**H**	**-0.72433**	**-2.37102**	**-0.02151**

**C**	**-2.78985**	**-0.7319**	**-0.02161**
**O**	**-3.1938**	**-1.85875**	**-0.01844**
**O**	**-3.625**	**0.36842**	**-0.02464**
**C**	**0.7576**	**2.77942**	**-0.00693**
**O**	**-0.01629**	**3.69267**	**-0.00318**
**O**	**2.12808**	**2.9523**	**-0.00744**
**C**	**2.0253**	**-2.04777**	**-0.00612**
**O**	**3.20287**	**-1.83305**	**0.00994**
**O**	**1.49142**	**-3.32172**	**-0.02045**
**C**	**2.38523**	**-4.37234**	**-0.00886**
**C**	**2.8509**	**-4.90986**	**-1.20374**
**C**	**2.79518**	**-4.92791**	**1.19817**
**C**	**3.70356**	**-6.00667**	**-1.19641**
**C**	**3.64752**	**-6.0248**	**1.2138**
**C**	**4.10066**	**-6.56364**	**0.01448**
**C**	**2.59384**	**4.25064**	**-0.00209**
**C**	**2.83474**	**4.91361**	**-1.20044**
**C**	**2.86665**	**4.89034**	**1.20201**
**C**	**3.36329**	**6.19833**	**-1.19937**
**C**	**3.3958**	**6.17483**	**1.21138**
**C**	**3.64424**	**6.82741**	**0.0087**
**C**	**-4.98205**	**0.12155**	**-0.01173**
**C**	**-5.6834**	**-0.00704**	**-1.2055**
**C**	**-5.66542**	**0.04089**	**1.19663**
**C**	**-7.05987**	**-0.19477**	**-1.19529**
**C**	**-7.04183**	**-0.14618**	**1.21509**
**C**	**-7.73801**	**-0.26361**	**0.01694**
**F**	**-5.0297**	**0.04726**	**-2.36773**
**F**	**-7.73166**	**-0.31135**	**-2.34215**
**F**	**-9.05878**	**-0.4421**	**0.0305**
**F**	**-7.69598**	**-0.21565**	**2.37592**
**F**	**-4.99385**	**0.14136**	**2.3456**
**F**	**2.55396**	**4.31233**	**-2.35836**
**F**	**2.6167**	**4.26713**	**2.35533**
**F**	**3.66373**	**6.78401**	**2.36785**
**F**	**4.15325**	**8.05925**	**0.01401**
**F**	**3.59972**	**6.82984**	**-2.35065**
**F**	**2.36923**	**-4.40218**	**2.34853**
**F**	**2.47836**	**-4.36699**	**-2.36456**
**F**	**4.14342**	**-6.52455**	**-2.34472**
**F**	**4.91713**	**-7.61712**	**0.02591**
**F**	**4.03395**	**-6.56012**	**2.37322**

9a (HF = -2272.3552288)

**C**	**1.33403**	**1.34236**	**-0.04588**
**C**	**1.03428**	**2.70893**	**-0.10565**
**C**	**-0.28802**	**3.14598**	**-0.12249**
**C**	**-1.3166**	**2.19946**	**-0.1042**
**C**	**-1.02371**	**0.83711**	**-0.03371**
**C**	**0.30506**	**0.40461**	**-0.00295**
**H**	**1.86562**	**3.40178**	**-0.16792**

H	-2.3393	2.54856	-0.15206
H	0.51819	-0.6545	0.04813
C	-2.07909	-0.20796	0.00954
O	-1.89431	-1.38872	0.08724
O	-3.34255	0.35123	-0.04967
C	2.76802	0.96035	-0.03404
O	3.69591	1.71895	-0.04993
O	2.92397	-0.41284	0.00179
C	4.21513	-0.89433	0.02558
C	4.82477	-1.19268	1.23933
C	4.90239	-1.12804	-1.16077
C	6.10103	-1.7407	1.2702
C	6.1797	-1.67376	-1.13816
C	6.77755	-1.98085	0.07935
C	-4.41182	-0.5171	-0.02033
C	-5.08585	-0.74603	1.17433
C	-4.85499	-1.13582	-1.18463
C	-6.20528	-1.56858	1.20473
C	-5.97051	-1.96296	-1.16136
C	-6.6464	-2.17709	0.03506
F	-4.65503	-0.17307	2.30034
F	-6.85488	-1.77899	2.35182
F	-7.72001	-2.96755	0.05997
F	-6.39531	-2.5522	-2.28103
F	-4.20247	-0.93534	-2.33118
F	4.33183	-0.82421	-2.32819
F	4.1801	-0.94987	2.38273
F	6.67987	-2.03346	2.43661
F	8.00227	-2.50709	0.10486
F	6.83475	-1.90194	-2.27829
C	-0.69527	4.59822	-0.2144
O	-1.80337	4.90789	-0.62158
N	0.24197	5.50821	0.17374
H	1.06177	5.18471	0.66049
C	-0.02334	6.93806	0.16334
H	-0.28584	7.30846	1.15937
H	0.85345	7.47863	-0.19847
H	-0.86156	7.12123	-0.50472

10a (HF=-1564.426622)

C	-3.34026	-1.07017	-0.11584
C	-3.76369	0.25432	0.04086
C	-2.84735	1.29698	0.16775
C	-1.48035	1.00916	0.14769
C	-1.04566	-0.30256	-0.03757
C	-1.97627	-1.33977	-0.1735
H	-4.83391	0.4212	0.07759
H	-0.77661	1.82016	0.27679
H	-1.5939	-2.33908	-0.34644
C	0.38949	-0.67002	-0.1041
O	0.82712	-1.77456	-0.26759
O	1.20421	0.43842	0.04975

**C**	**2.56354**	**0.22324**	**0.02092**
**C**	**3.27756**	**0.44545**	**-1.15174**
**C**	**3.24302**	**-0.17274**	**1.16842**
**C**	**4.65887**	**0.29593**	**-1.17478**
**C**	**4.62304**	**-0.32848**	**1.15231**
**C**	**5.33078**	**-0.09174**	**-0.02109**
**F**	**2.63329**	**0.80372**	**-2.26437**
**F**	**5.34118**	**0.51854**	**-2.30065**
**F**	**6.65666**	**-0.23649**	**-0.03929**
**F**	**5.27121**	**-0.70448**	**2.25731**
**F**	**2.56352**	**-0.40711**	**2.2931**
**C**	**-3.23944**	**2.74154**	**0.37071**
**O**	**-2.45107**	**3.54074**	**0.85177**
**N**	**-4.50434**	**3.07628**	**-0.01059**
**H**	**-5.04391**	**2.41248**	**-0.54143**
**C**	**-4.41951**	**-2.1166**	**-0.25173**
**O**	**-5.56159**	**-1.79841**	**-0.54996**
**N**	**-4.0392**	**-3.40549**	**-0.02801**
**H**	**-3.13121**	**-3.58186**	**0.36918**
**C**	**-5.01029**	**4.43417**	**0.10057**
**H**	**-6.02896**	**4.42618**	**0.49424**
**H**	**-4.36348**	**4.97924**	**0.78409**
**H**	**-5.00604**	**4.94749**	**-0.86631**
**C**	**-4.97771**	**-4.513**	**-0.1022**
**H**	**-5.86118**	**-4.16996**	**-0.63541**
**H**	**-4.53128**	**-5.35076**	**-0.64216**
**H**	**-5.2815**	**-4.85195**	**0.89328**

Table (S7). Experimental and computed values for reactivity of esters[a]

**Entry**	**Parameter**	**3**	**9a**	**10a**
1	$\delta_{C=O(ester)}$ (ppm)[b]	160.53	161.1	161.70
2	C=O (Å)	1.1971	1.1981	1.1993
3	π^* occ. (C=O)[c]	0.2174	0.2210	0.2251
4	ω (eV)[d]	2.9041	2.6626	2.2939
5	LUMO (eV)	-2.8817	-2.6564	-2.2907
6	V_C (au)[e]	-14.6025	-14.6093	-14.6166
7	charge (NBO)	0.8216	0.8224	0.8230

[a] Data presented (aside from entry 1) are derived from quantum chemical calculations (at the B3LYP/6-311G* level) unless specified otherwise. Structures **9a** and **10a** consisted of a truncated methyl-substituted amide. Data is presented as the average of multiple esters unless specified. [b] ^{13}C NMR chemical shifts for the ester carbonyl carbons (in $CDCl_3$). [c] Occupancy of the ester carbonyl π^* orbital from NBO population analysis. [d] Calculated global electrophilicity index. [e] Electrostatic potential at nuclei (EPN).

NBO Charges					EPN (atomic units)				C=O angstroms			
	C10	C13	C16	average C=O	C10	C13	C16	average C=O	C10-O11	C13-O14	C16-O17	average C=O
3	0.82162	0.82166	0.82163	0.821636667	-14.602471	-14.6025	-14.6025	-14.602467	1.19707	1.19706	1.19709	1.197073333
	C10	C13	Am_C38		C10	C13			C10-O11	C13-O14	C38-O39	
9a	0.82226	0.82246	0.67607	0.82236	-14.611169	-14.6075		-14.6093175	1.19765	1.19862	1.22049	1.198135
	C10	Am_C28	Am_C24		C10				C10-O11	C28-O29	C24-O25	
10a	0.82304	0.67764	0.67692	0.82304	-14.616601			-14.616601	1.19927	1.22252	1.22133	1.19927
Pi* occupancy	BD*(2)	BD*(2)	BD*(2)		LUMO		HOMO		Global Electrophilicity			
	C10-O11	C13-O14	C16-O17	average C=O	A.U.	eV	A.U.	eV	A.U.	eV		
3	0.21738	0.21736	0.2174	0.21738	-0.1059	-2.88169	-0.28348	-7.71388657	0.106724282	2.904117		
	C10-O11	C13-O14	C38-O39									
9a	0.21903	0.22295	0.30195	0.22099	-0.09762	-2.65638	-0.27491	-7.4806849	0.097847172	2.662558		
	C10-O11	C28-O29	C24-O25									
10a	0.22505	0.30813	0.30463	0.22505	-0.08418	-2.29066	-0.26579	-7.23251697	0.084300838	2.293943		

Synthesis of molecule 15:

A dry round bottom flask was charged with **11** (20 mg, 0.06 mmol, 1 equiv.) under nitrogen atmosphere and dissolved in 4mL anhydrous DCM. It was stirred until fully dissolved and then solution of anhy DIPEA (9.8 µL, 0.06 mmol, 1 equiv.) in 2mL anhy DCM was added into the flask. The reaction flask was transferred to the ice bath and subsequently, hexylamine (3.7 µL, 0.03 mmol, 0.5 equiv.) solution in DCM (3 mL) was added dropwise to the reaction flask in 15 minutes under continuous stirring. The reaction mixture was stirred for 1.5 hours and then the reaction mixture was vacuum dried. **15** was separated by running flash column chromatography on silica gel using eluent DCM/acetonitrile (95/5) by volume. **15** was obtained white powder in 50% (9 mg) isolated yield. ^{1}H NMR (700 MHz, $CDCl_3$) δ 8.68 (2H, d), 8.52 (1H, t), 6.43 (2H, t), 3.50 (4H, m), 1.65 (4H, p), 1.43–1.20 (24H, m), 0.93–0.85 (6H, dt). ^{13}C NMR (176 MHz, $CDCl_3$) δ 165.28, 161.73, 136.32, 131.54, 131.44, 128.12, 53.61, 40.72, 32.08, 31.64, 29.82, 29.80, 29.77, 29.72, 29.71, 29.68, 29.52, 29.48, 27.17, 26.83, 22.85, 22.70, 14.28, 14.18. MS (MALDI-TOF), calcd. for $C_{33}H_{43}F_5N_2O_4$: 626.31, found: 627.23 $[M+H]^+$.

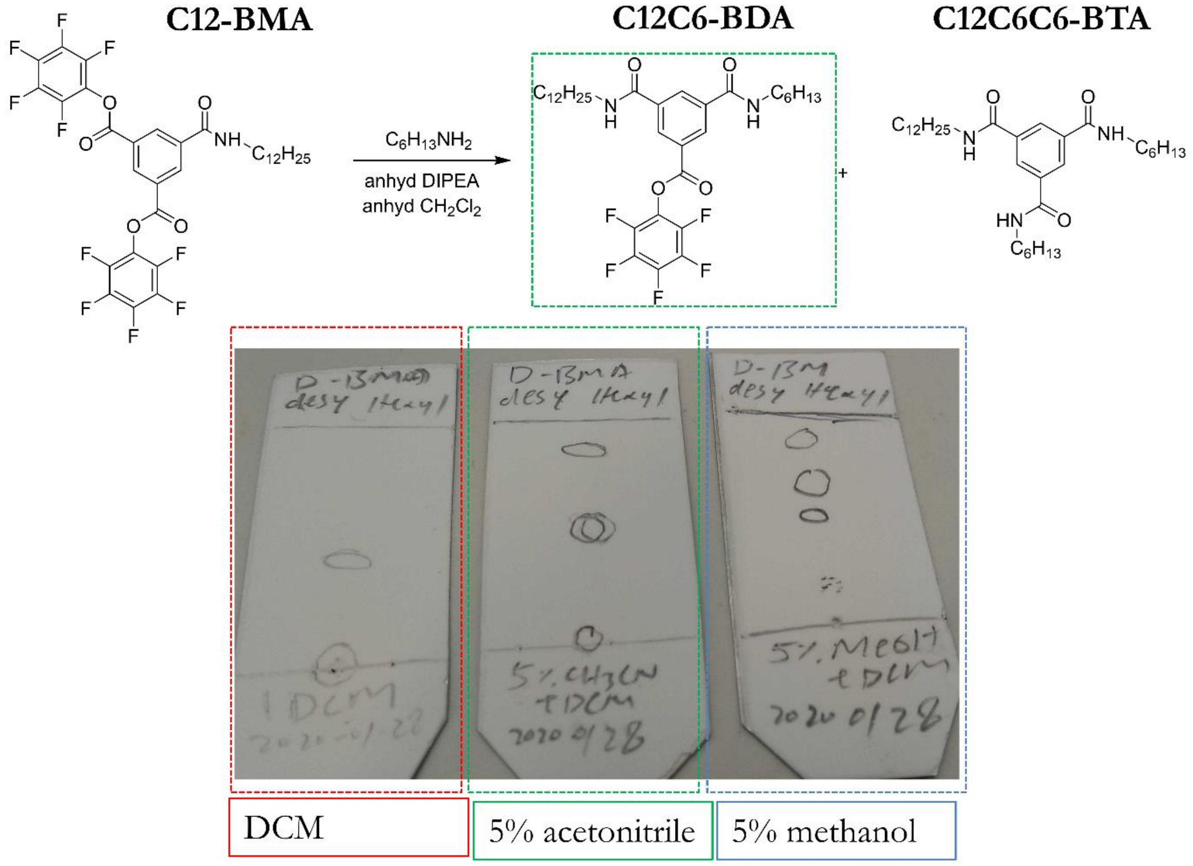

Figure S34: Reaction scheme of synthesis of molecule 15. Using hexylamine, **11** was desymmetrized for creating molecule **15**.

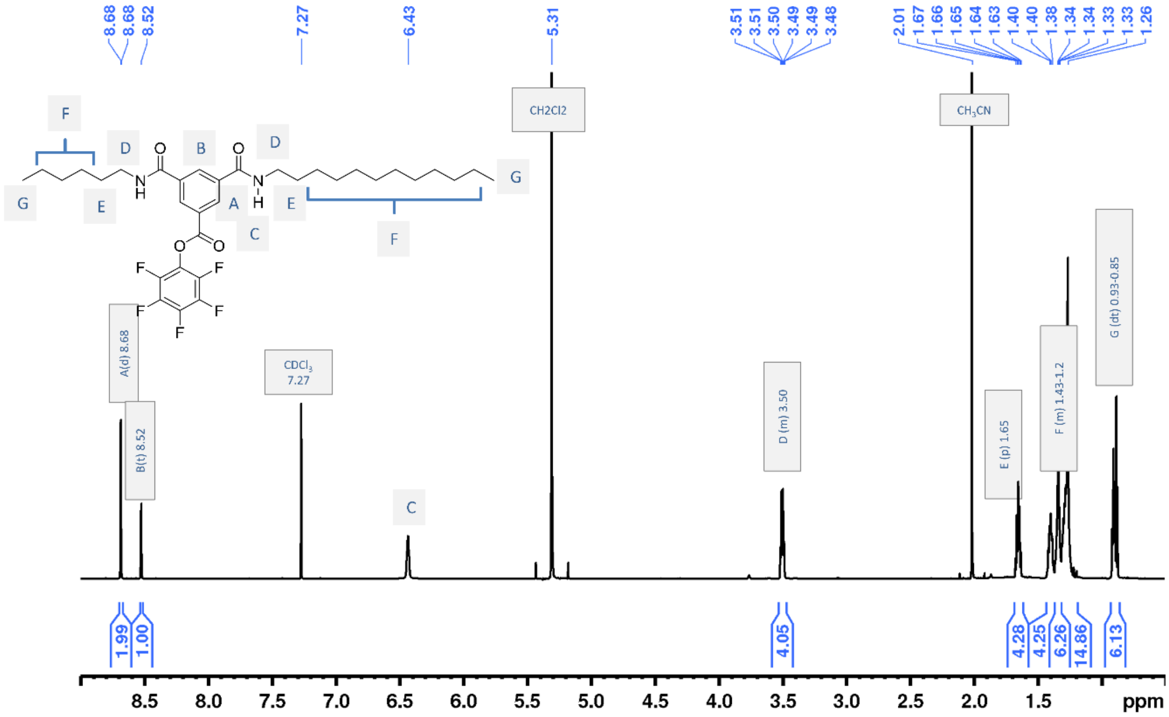

Figure S35: ^{1}H NMR ($CDCl_3$) of **15**

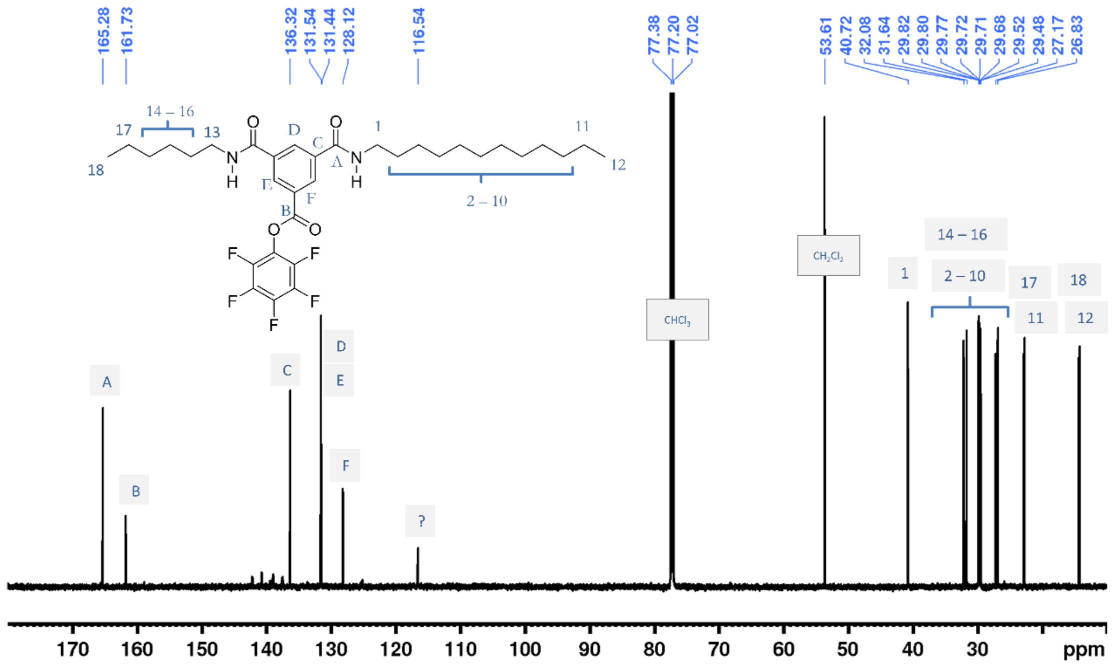

Figure S36: ^{13}C NMR ($CDCl_3$) of molecule **15**

Synthesis of molecule 16:

A dry round bottom flask was added **9** (75 mg, 0.24 mmol, 1 equiv.) under nitrogen atmosphere and dissolved in 6mL anhydrous DCM. Subsequently solution of anhydrous DIPEA (27 µL, 0.16 mmol, 0.65 equiv.) in 12mL anhydrous DCM was added into the reaction flask. 5Nb-2MA (11.6 µL, 0.1 mmol, 0.4 equiv.) solution in DCM (6 mL) was added dropwise to the reaction flask in 10-15 minutes under inert atmosphere. After 2 hours of continuous stirring, reaction mixture was vacuum dried. Using eluent DCM/acetonitrile (95/5) by volume**,** **16** (C_6Nb-BDA) was separated on silica gel flash column. **16** was obtained as white powder in 44% (30mg) yield. ^{1}H NMR (700 MHz, $CDCl_3$) δ 8.69-8.63 (m, 2H, Ar), 8.55-8.49 (m, H, Ar), 6.68 (t, 1H, H_c), 6.57 (t, 1H, H_c), 6.22(dd, H, H_5), 6.08 (m, 2H, $H_{5,6}$), 6.02(dd, H, H_6), 3.48(q, 2H H_D), 3.22(m, 2H, H_8), 2,89 (bs, H, H_4), 2.85 (bs, H, H_1), 2.68 (m, H, H_{2exo}), 2.39 (m, H, H_{2endo}), 2.01 (bs, 2H, H_7), 1.92 (m, 2H, H_7), 1.7 (m, 2H, H_7), 1.64 (p, 2H, H_E), 1.47 (dd, 2H, H_3), 1.4 (m, 2H, H_F), 1.32(m, 4H, H_G), 0.89 (t, 3H, H_H), 0.65 (dd, 2H, H_3). ^{13}C NMR δ 165.29, 165.24, 161.67, 138.16, 137.16, 136.29, 136.26, 132.09, 131.51, 131.45, 131.40, 128.01, 49.73, 45.79, 45.22, 44.62, 44.49, 42.58, 41.92, 40,67, 39,26, 38.98, 31.60, 31.15, 30.32, 29.63, 26.79, 22.67, 14.13.

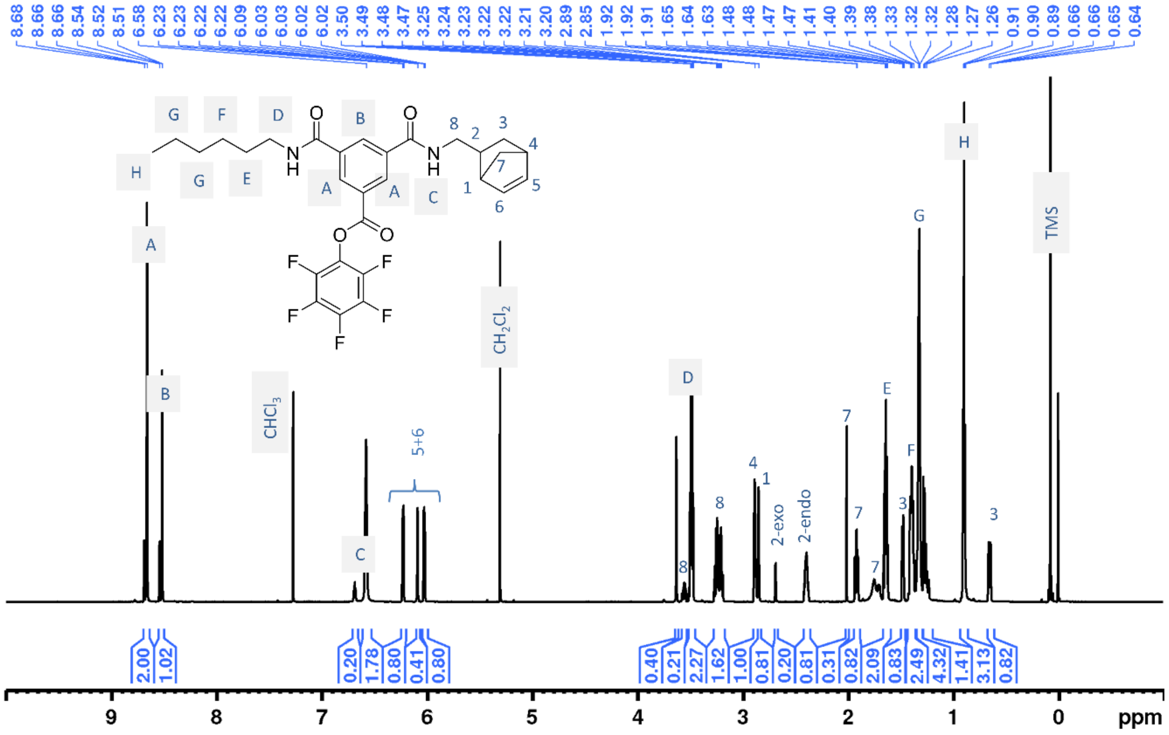

Figure S37: ^{1}H NMR ($CDCl_3$) of molecule **16**

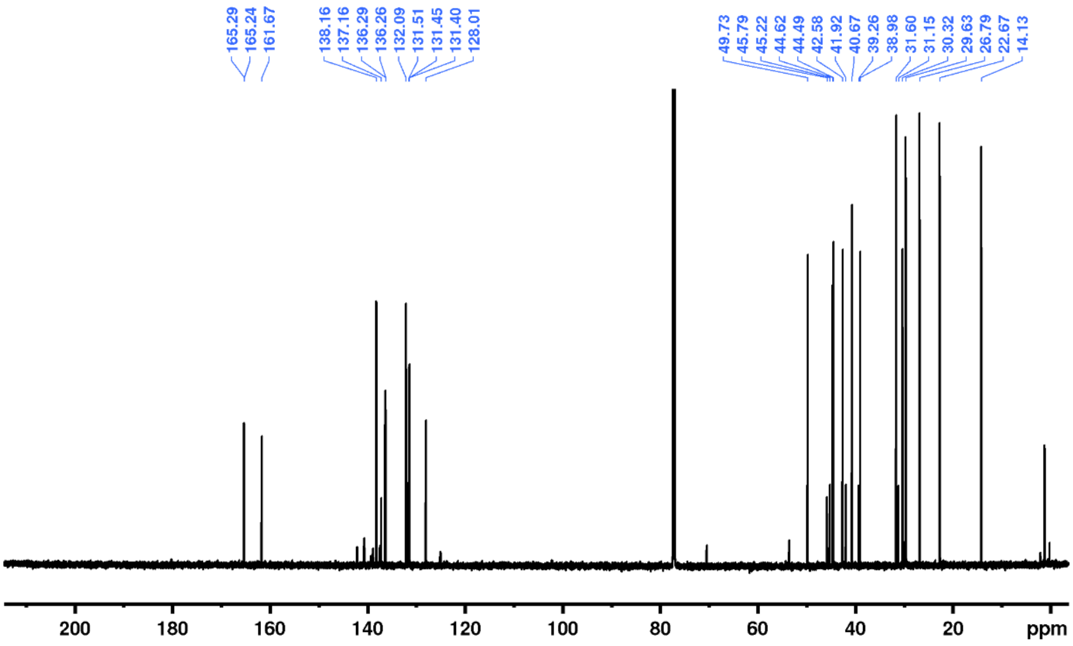

Figure S38: ^{13}C NMR ($CDCl_3$) of molecule **16**

Synthesis of molecule 17

To a clean and dry round bottom flask was added **12** (25 mg, 0.035 mmol, 1 equiv.) and dissolved in anhydrous dichloromethane (DCM, 4 mL) under inert atmosphere. To a stirring reaction solution was added dry DIPEA (13.5 µL, 0.077 mmol, 2.1 equiv.) and 5Nb-2MA (9.4 µL, 0.074 mmol, 2.1 equiv) solution in anhydrous DCM (3 mL) was added to the reaction flask. After overnight stirring excess DCM was removed *in vacuo* and **12** was obtained via column chromatography using eluent methanol/dichloromethane (5/95) by volume. Isolated yield of the reaction was 87% (20.1 mg). ^{1}H NMR spectrum is shown in **Figure S39**. It was not possible to clearly evaluate peak splitting pattern for some of the peaks in ^{1}H NMR and we leave all the information in the ^{1}H NMR spectrum. ^{1}H NMR (700 MHz, $CDCl_3$) δ 7.73 (bs, 3H, Ar), 7.48 (bs, 3H, H_e), 6.17 (dd, H, H_{5endo}), 6.09 (dd, H, H_{6exo}), 6.05 (dd, 2H, H_{5exo}), 6.02(dd, H, H_{6endo}), 3.6 (m, 2H, H_8), 3.35 (m, 4H, H_B), 3.14 (m, H_8), 3.0 (m, 2H, H_7), 2,84 (bs, H, H_4), 2.81 (bs, H, H_1), 2.64 (m, H, H_{2exo}), 2.34 (m, H, H_{2endo}), 1.86 (m, 2H, H_3), 1.58 (p, 4H, H_B), 1.22-1.37 (m, 36H, H_D), 0.88 (t, 6H, H_A), 0.6 (m, 2H, H_3). Perhaps that could be already BTA stacking of the moelcules in $CDCl_3$.^{13}C NMR δ 166.43, 166.39, 137.90, 137.00, 136.42, 135.56, 135.49, 132.30, 128.03, 127.99, 53.75, 49.69, 45.67, 45.22, 44.48, 44.46, 42.58, 42.04, 41.93, 40.54, 39.21, 38.96, 32.06, 30.34, 29.82, 29.79, 29.74, 29.65,29.50, 27.20, 22.83. MS (MALDI-TOF), calcd. for $C_{41}H_{67}N_3O_3$: 649.52, found: 650.37 $[M+H]^+$.

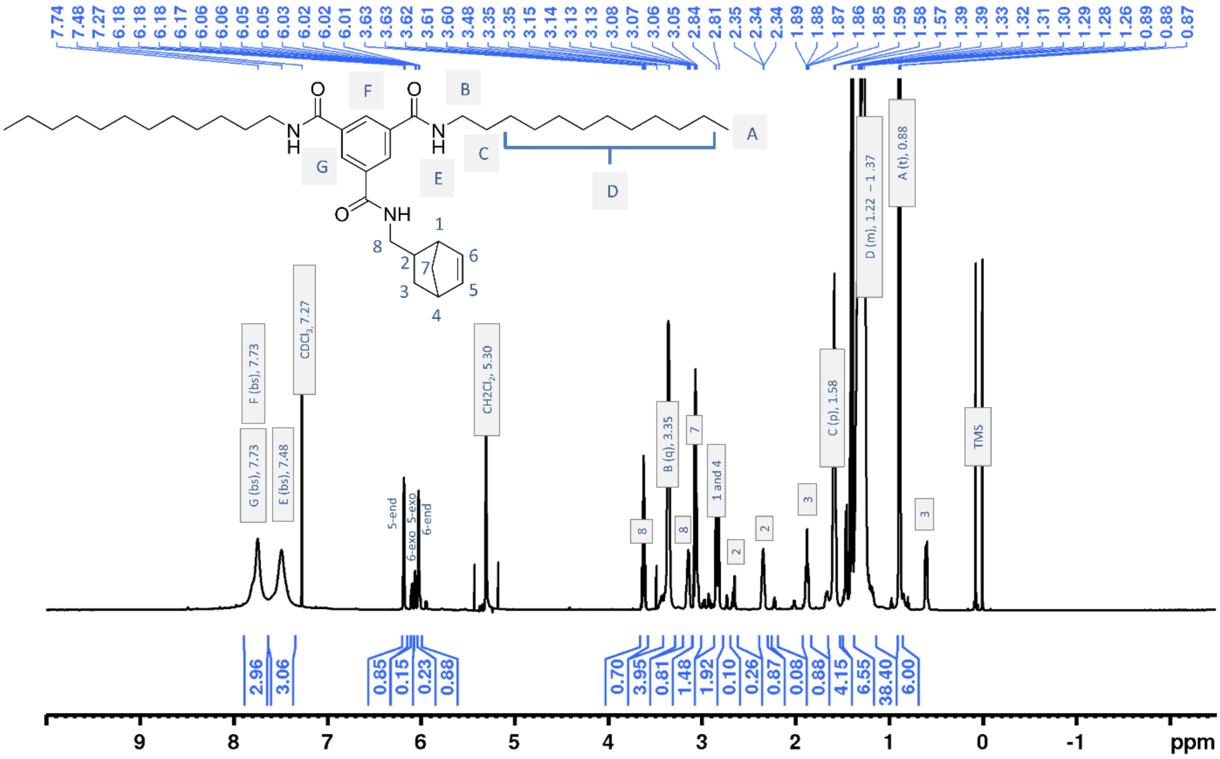

Figure S39: ^{1}H NMR ($CDCl_3$) molecule **17**

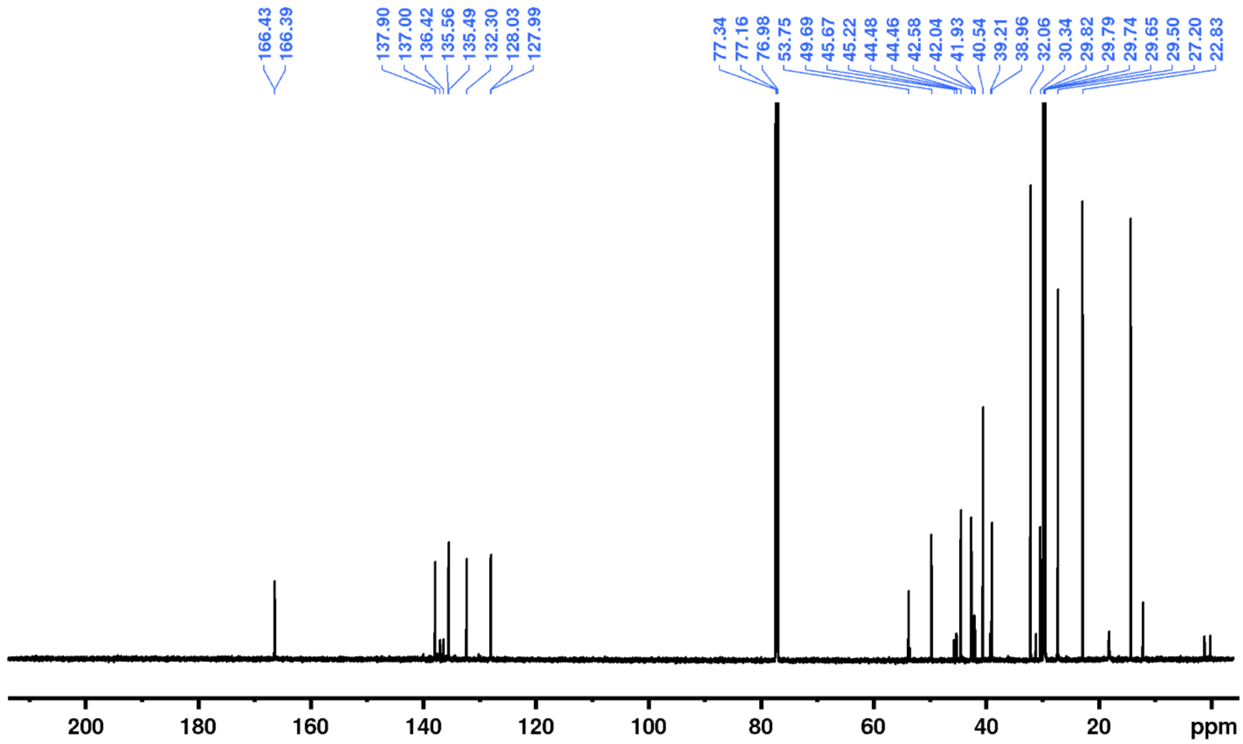

Figure S40: ^{13}C NMR ($CDCl_3$) molecule **17**

Synthesis of molecule 18:

A clean and round bottom flask equipped with a stir bar was charged with **12** (25 mg, 0.035 mmol, 1 equiv.) and dissolved in anhydrous dichloromethane (DCM, 4 mL) under inert atmosphere. The reaction solution was stirred until **12** dissolved and then to a stirring solution was added solution of DIPEA (13.5 μL, 0.08 mmol) and 3-Azido-1-propanamine (0.01 g, 0.074 mmol, 2.1 equiv.) in anhydrous DCM (3 mL) was added. After overnight stirring at RT, the reaction mixture was vacuum dried to remove excess solvent (DCM) and **12** was obtained in 85% yield (19 mg) by running flash column chromatography on silica gel using eluent methanol/dichloromethane (5/95) by volume. ^{1}H NMR (700 MHz, $CDCl_3$) δ 7.82 (bs, 2H, Ar), 7.59 (bs, H, Ar), 7.24 (bs, 3H, (C=O)N<u>H</u>), 3.49 (q, 2H,(C=O)NHC<u>H</u>$_2$CH$_2$CH$_2$N$_3$), 3.41 (t, 2H, C<u>H</u>$_2$N$_3$), 3.38 (q, 4H, (C=O)NHC<u>H</u>$_2$), 1.89 (p, 2H, C<u>H</u>$_2$CH$_2$N$_3$), 1.58(p, 4H, (C=O)NHCH$_2$C<u>H</u>$_2$), 1.26–1.43 (m, 36H, aliphatic), 0.88 (t, 6H, aliphatic). ^{13}C NMR (176 MHz, $CDCl_3$) δ 166.7, 166.43, 135.62, 135.26, 127.95, 127.82, 49.38, 40.53, 37.85, 32.07, 29.84, 29.81, 29.76, 29.64, 29.51, 28.92, 27.20, 22.83, 14.26. MS (MALDI-TOF), calcd. for $C_{36}H_{62}N_6O_3$: 626.49, found : 627.43 [M+H]$^+$.

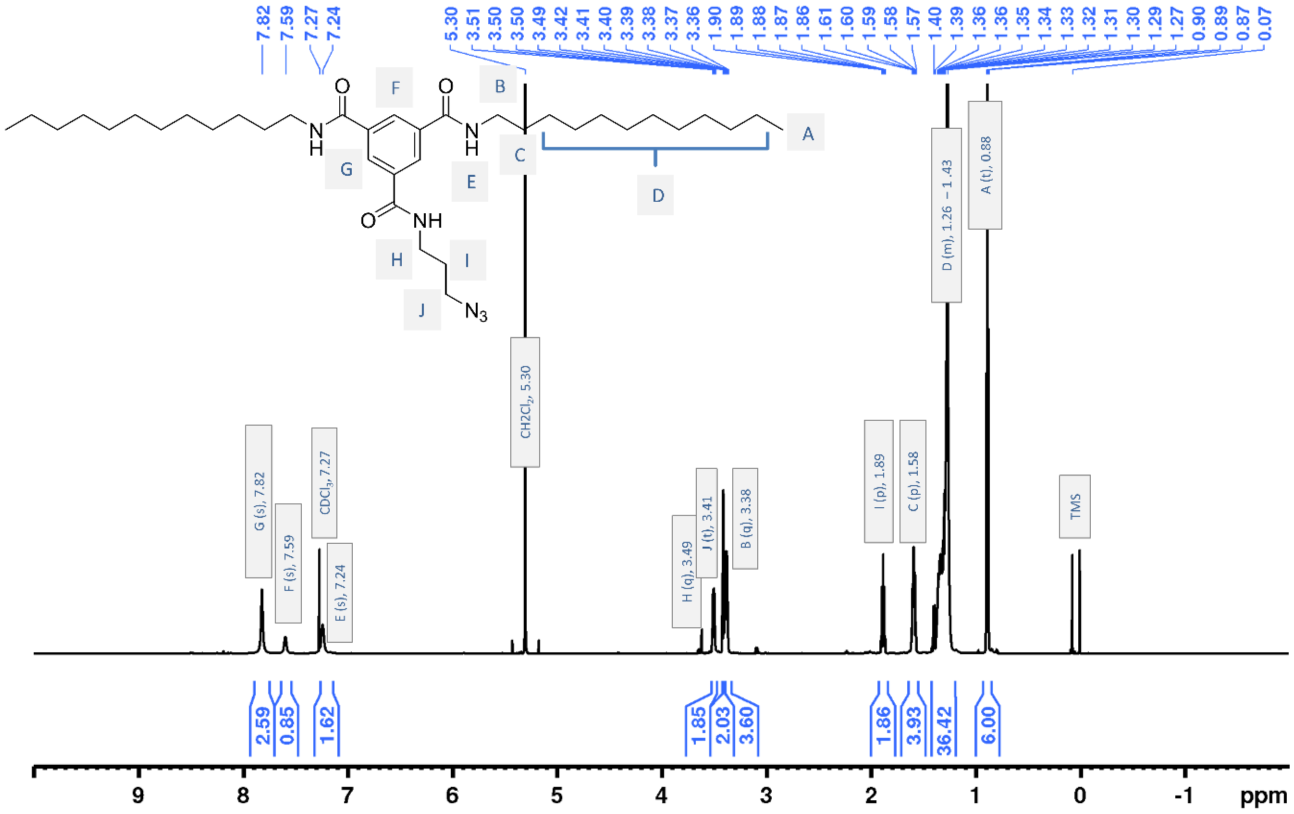

Figure S41: ^{1}H NMR ($CDCl_3$) molecule **18**

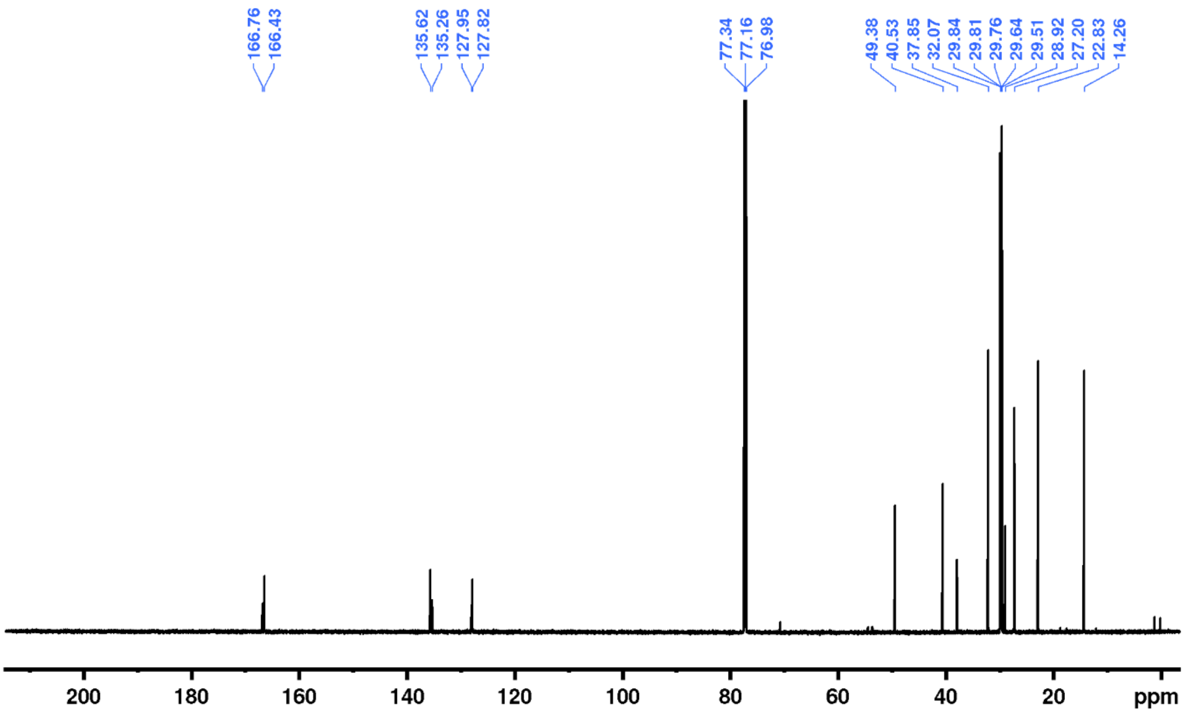

Figure S42: ^{13}C NMR ($CDCl_3$) molecule **18**

Synthesis of molecule 19

A dry round bottom flask was charged with **16** (20 mg, 0.04 mmol, 1 equiv.) under nitrogen atmosphere and dissolved in 2 mL anhydrous DCM. Subsequently solution of anhydrous DIPEA (13.5 µL, 0.08 mmol, 2 equiv.) in 2 mL anhydrous DCM was added into the reaction flask. Next, 3-Azido-1-propanamine (6.1 µL, 0.06 mmol, 1.6 equiv.) solution in DCM (2 mL) was added to the reaction flask using a glass pipette. The reaction was stirred overnight and then the reaction mixture was vacuum dried to make the reaction solution concentrated. C6Nbpropylazide-BTA was separated by running flash column chromatography on silica gel using eluent DCM/MeOH (95/7.5) by volume. Molecule **19** was obtained white powder in 85% (17 mg). ^{1}H NMR (700 MHz, $CDCl_3$) δ δ 7.73 (bs, 3H, Ar), 7.56 (bs, 1H, H_b), 7.29 (bs, 1H, H_c), 6.20 (dd, 1H, H_{5endo}), 6.12-6.06 (m, 2H, $H_{5,6exo}$), 6.02(dd, H, H_{6endo}), 3.64-3.33(m), 3.20-2.7 (m), 2.65 (bs, H, H_{2exo}), 2.34 (m, H, H_{2endo}), 2.05-1.78 (m), 1.7-1.54 (m), 1.42-1.19 (m), 0.90 (m, 3H, H_H), 0.65 (dd, 2H, H_3). ^{13}C NMR (176 MHz, d_6-DMSO) δ 165.38, 135.1, 134.86, 128.32, 54.91, 40.02, 31.00, 28.99, 26.15, 22.04, 13.91. since we took carbons pectra in *d6*-DMSO which showed septet at 39.52 and a number of peaks from hexyl, methyl norbornene and propyl hid under d6-DMSO peaks. MS (MALDI-TOF), calcd. for $C_{26}H_{36}N_6O_3$: 480.28, found : 481.21 $[M+H]^+$.

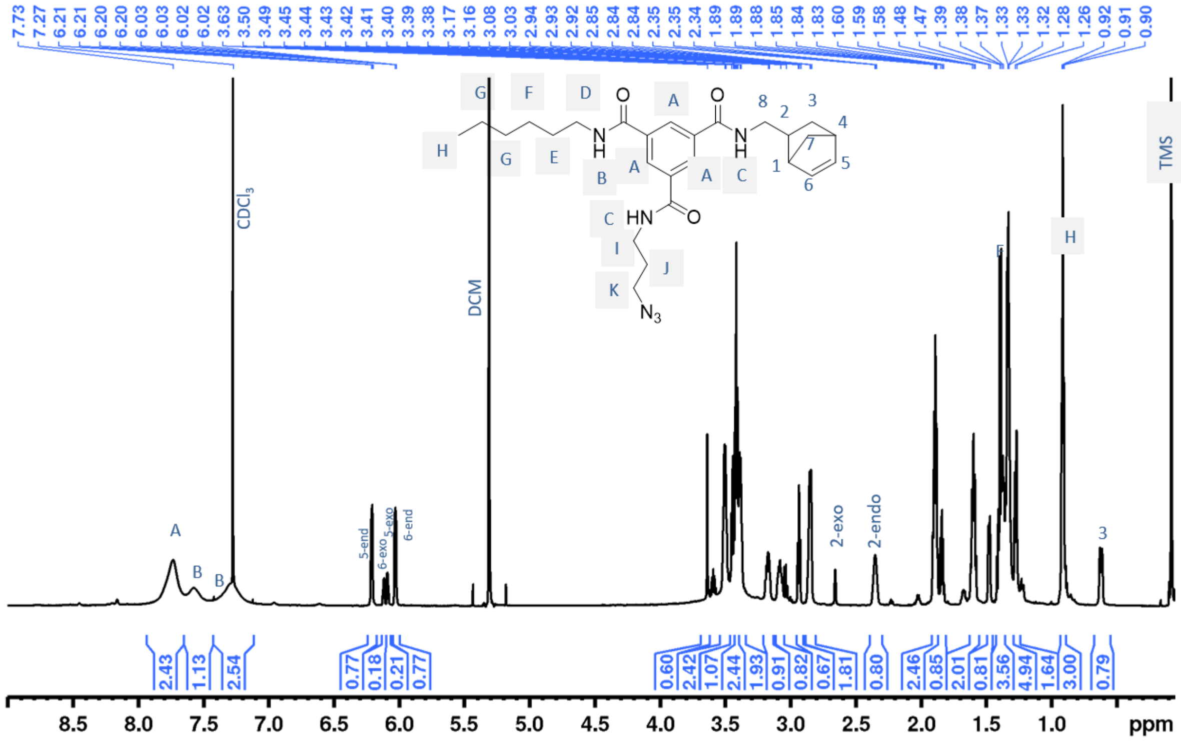

Figure S43: ^{1}H NMR of molecule **19**

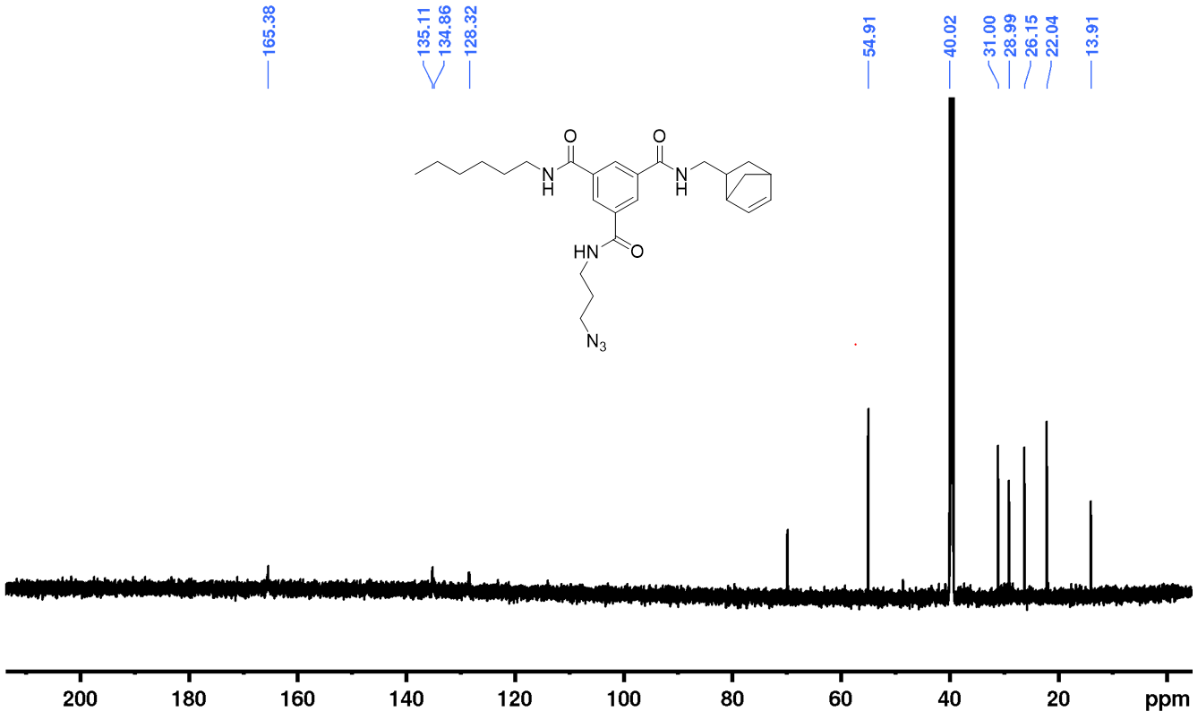

Figure S44: ^{13}C NMR of molecule **19**

PEG-CDI synthesis:

Bishydroxy PEG (20 kg/mol) was dried via azeotropic distillation using toluene. Dried bishydroxy PEG (30 g, 3 mmol, 1 equiv.) was dissolved in 100 mL of anhydrous 1,4-dioxane at 37 °C under N_2 atmosphere. Vacuum dried (at 50 °C for 3 hours) carbonyl diimidazole (CDI, 0.08 g, 0.5 mmol, 5 equiv. per OH) dissolved in 30 mL of anhydrous 1,4-dioxane was added to the reaction flask under nitrogen inert atmosphere. The reaction mixture was stirred at 37 °C for 3 hours. The reaction mixture was precipitated out in excess cold diethyl ether twice and dried overnight in a vacuum oven rotavap at 40 °C. The product was obtained as a white solid with a 96% (29 g) yield. ^1H NMR (700 MHz, $CDCl_3$) δ 8.11 (s, 2H, Ar), 7.61 (s, 2H, Ar), 7.40 (s, 2H, Ar), 4.51(t, 4H, $C\underline{H_2}OC{=}O$), 3.79 (t, 4H, $OC\underline{H_2}CH_2$), 3.4 (b, 1772H, O-$\underline{CH_2CH_2}$-O). The mass of the polymer is 19313 g/mol with Đ of 1.1.

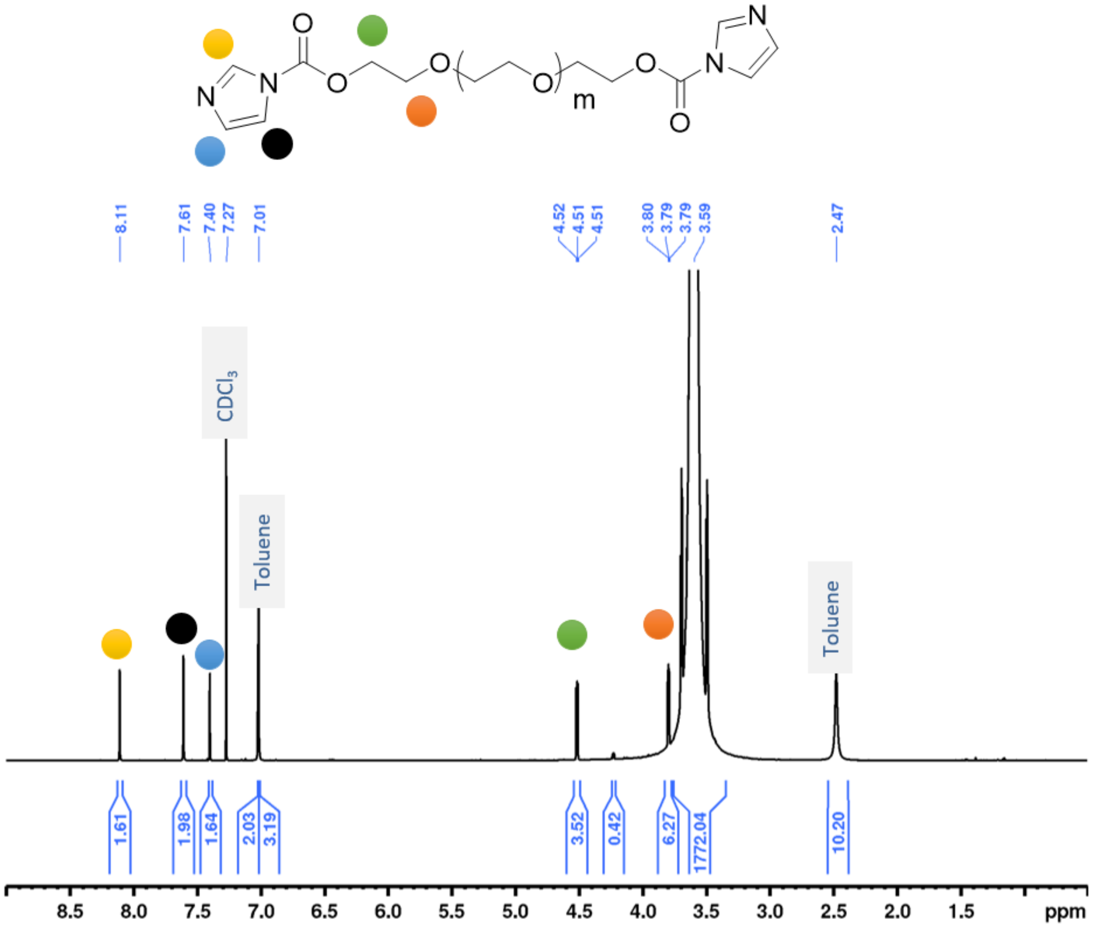

Figure S45: ^1H NMR ($CDCl_3$) of PEG-CDI

PEG-Bisamino dodecane synthesis:

PEG-CDI (27 g, 1.34 mmol, 1 equiv.) was vacuum dried at 60 ºC for 3 hours and dissolved in 270 mL anhydrous DMF. The polymer solution was added dropwise to the solution of 1,12 diaminododecane (3.5 g, 19 mmol, 14 equiv.) dissolved in 275 mL of anhydrous DMF and maintained at 70 ºC. The reaction mixture at 70 ºC was stirred for 24 hours under nitrogen atmosphere. The reaction mixture was concentrated by removing DMF and precipitated in excess cold diethyl ether. The product was again dissolved in DCM and precipitated out in excess cold diethyl ether. The product was obtained as a white solid in 97% yield. The mass of the polymer is 25760 g/mol with Đ of 1.2. ^1H NMR (700 MHz, $CDCl_3$) δ 4.83 (s, 2H, N<u>H</u>C=O), 4.20 (t, 4H, C<u>H</u>$_2$OC=O), 3.7 (b, 1776H, O<u>(CH$_2$)$_2$</u>O), 3.15 (q, 4H, C_{H2}NHC=O), 2.68 (t, 4H, C<u>H</u>$_2$NH$_2$), 1.47-1.25 (m, 40H, aliphatic).

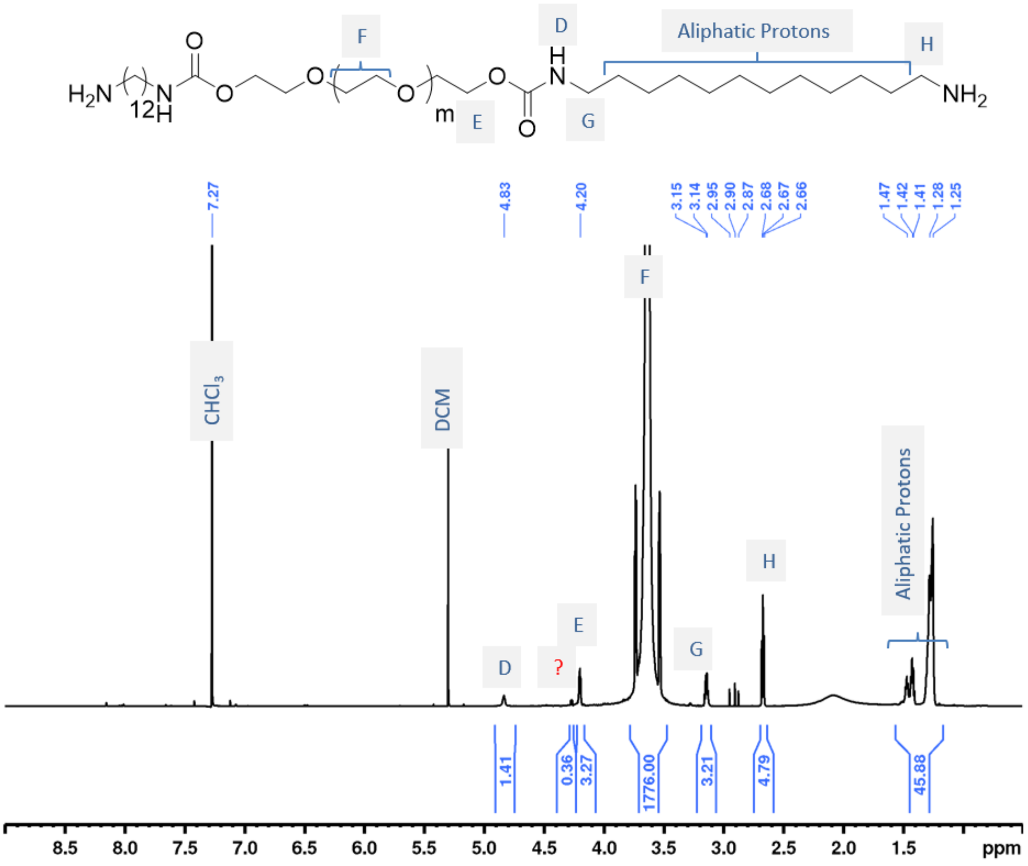

Figure S46: ^1H NMR ($CDCl_3$) of PEG bisaminododecane

Synthesis of hydrogelator C_6C_6

In a dry round bottom flask, **10** (0.2 g, 0.34 mmol, 1.1 equiv.) was dissolved in 8mL anhydrous DCM and DIPEA (0.06 g, 0.44 mmol, 1.5 equiv.) was added into the reaction flask. Subsequently, PEG bisaminododecane (3.2 g, 0.3 mmol, 1 equiv.) solution in anhydrous DCM (5–6 ml) was added dropwise to the reaction flask. The reaction mixture was stirred for 40 hours at room temperature (~20 °C) under nitrogen atmosphere. Excess solvent was removed in vacuo and the crude reaction mixture precipitated in excess cold diethyl ether obtaining BTA 1 hydrogelator, as a white powder in 98% yield. Second purification was done by dialyzing the sample in methanol against methanol to remove any unreacted small molecule impurities. Hydrogelator $\mathbf{C_6C_6}$ was obtained in 89% yield with the mass of 24344 g/mol and with Đ of 1.2. ^{1}H NMR (700 MHz, d_6-DMSO) 8.65-8.6 (m, 6H, NH(C=O)), 8.35 (s, 6H, Ar), 7.15 (t, 2H, CH2NH(C=O)O), 4.04-4.0 (t, 4H, NH(C=O)OCH2), 3.5 (bs, 1776H, O-(CH2)2-O), 3.29-3.24 (t, 12H, (C=O)NHCH2), 2.95-2.90 (q, 4H, CH2NH(C=O)O), 1.6-1.14 (mm, 72H, aliphatic), 0.88-084 (t, 12H, CH2CH3, aliphatic).

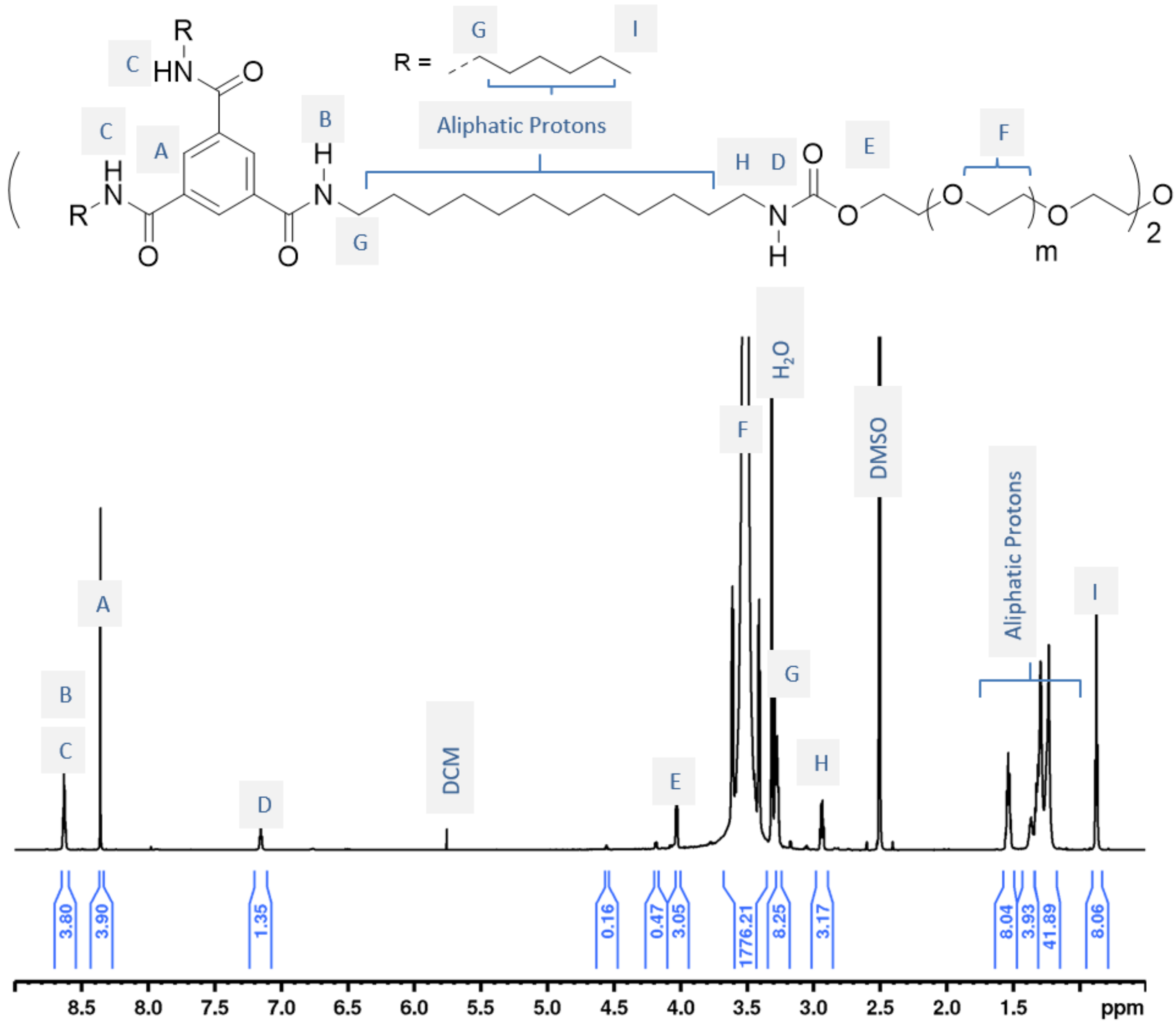

Figure S47: ^{1}H NMR (d_6-DMSO) of the hydrogelator $\mathbf{C_6C_6}$

Synthesis of hydrogelator C_6C_{12}

15 (0.03 g, 0.043 mmol, 1.1 equiv.) was dissolved in 3 mL anhydrous DCM and DIPEA (0.008 g, 0.06 mmol, 1.5 equiv.) was added to it. PEG bisaminododecane (0.4 g, 0.04 mmol, 1.0 equiv.) was weighed in dried flask, dissolved in anhydrous DCM (3–4 mL) and added to the flask. The reaction continued to run for 40 hours at 20 °C under nitrogen inert atmosphere. The reaction mixture was concentrated using vacuum and precipitated out using excess cold diethyl ether. The same procedure was repeated twice. The product was dried in a vacuum oven at 45 °C for 3 hours and the white product was obtained in 75% yield. Hydrogelator **C_6C_{12}** was obtained with mass 21207 g/mol with Đ of 1.2. ^{1}H NMR (700 MHz, d_6-DMSO) 8.65–8.58 (m, 6H, N<u>H</u>(C=O)), 8.36–8.32 (s, 6H, Ar), 7.17–7.12 (t, 2H, CH_2N<u>H</u>(C=O)O), 4.06–3.99 (t, 4H, N<u>H</u>(C=O)OC<u>H</u>$_2$), 3.64–3.37 (bs, 1776H, O-(C<u>H</u>$_2$)$_2$-O), 3.29–3.24 (m, 12H, (C=O)NHC<u>H</u>$_2$), 2.95–2.90 (q, 4H, C<u>H</u>$_2$NH(C=O)O), 1.56–1.17 (mm, 76H, aliphatic), 0.88–0.84 (t, 12H, CH_2C<u>H</u>$_3$, aliphatic).

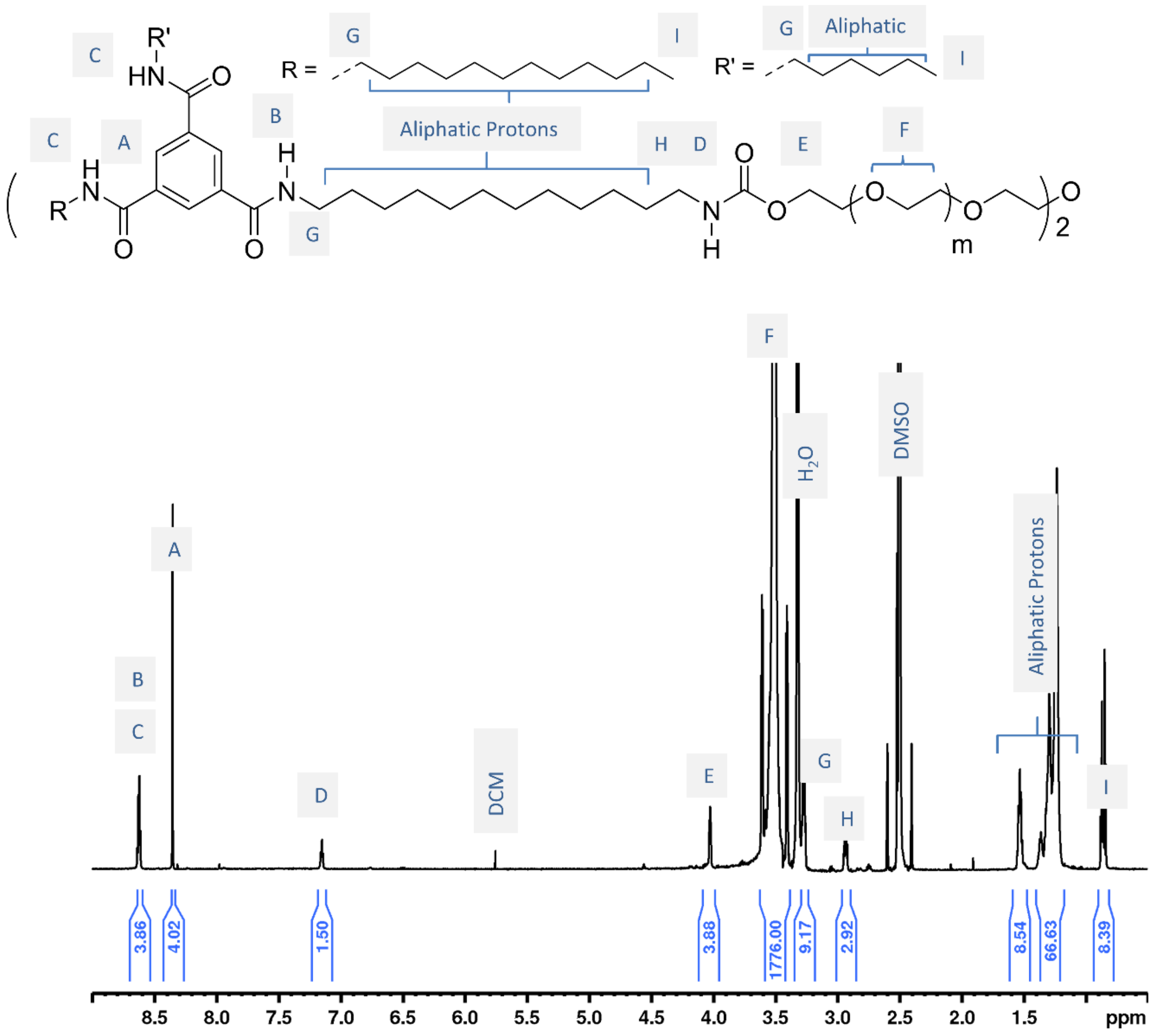

Figure S48: ^{1}H NMR (d_6-DMSO) of hydrogelator **C_6C_{12}**

Synthesis of hydrogelator $C_{12}C_{12}$

12 (C12C12, 0.23 g, 0.32 mmol, 1.1 equiv.) was dissolved in 8 mL anhydrous DCM and DIPEA (0.056 g, 0.44 mmol, 1.5 equiv.) was added to it. PEG bisaminododecane (3 g, 0.29 mmol, 1 equiv.) was weighed in dried flask, dissolved in anhydrous DCM (5-6 mL) and added to the flask. The reaction continued to run for 40 hours at 20 °C under an inert atmosphere. Reaction mixture was first purified by precipitation in cold diethyl ether and the pure molecule was obtained in 98% isolated yield as a white powder. Later also sample was dissolved in methanol and dialyzed against methanol for further purification. The pure molecule was obtained in 84% yield. Hydrogelator $\mathbf{C_{12}C_{12}}$ was obtained in 84% yield with the mass of 23573 g/mol and with Đ of 1.2. 1H NMR (700 MHz, d_6-DMSO) 8.65-8.57 (t, 6H, NH(C=O)), 8.38-8.31 (s, 6H, Ar), 7.18-7.10 (t, 2H, CH2NH(C=O)O), 4.06-3.94 (t, 4H, NH(C=O)OCH2), 3.6-3.37 (bs, 1776H, O-(CH2)2-O), 3.29-3.22 (t, 12H, (C=O)NHCH2), 2.96-2.90 (q, 4H, CH2NH(C=O)O), 1.59-1.15 (mm, 120H, aliphatic), 0.88-0.80 (dt, 12H, CH2CH3, aliphatic).

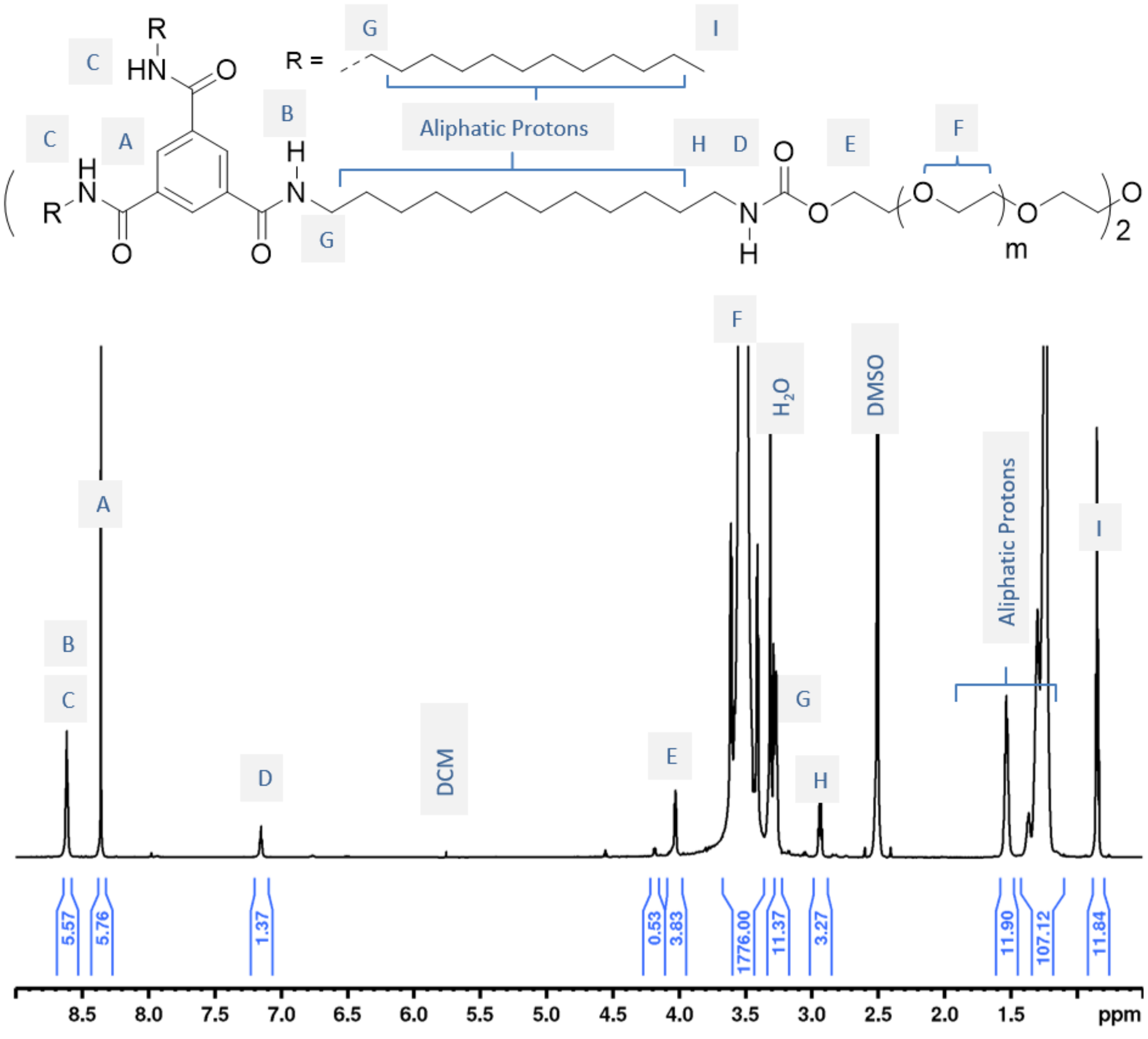

Figure S49: ^{1}H NMR (d_6-DMSO) of hydrogelator $\mathbf{C_{12}C_{12}}$

Table S8: **Details on molecular weight average for hydrogelator *C_6C_6*, *C_6C_{12}* and *$C_{12}C_{12}$*. All the samples were dissolved in DMF at concentrations around 5 mg/mL, filtered through 0.2 µm filters before being injected into the GPC system for analysis.**

Hydrogelator name	M_n (g/mol)	M_w (g/mol)	Đ (M_w/M_n)
C_6C_6	20,039	24,344	1.2
C_6C_{12}	18,146	21,207	1.16
$C_{12}C_{12}$	19,500	23,573	1.2

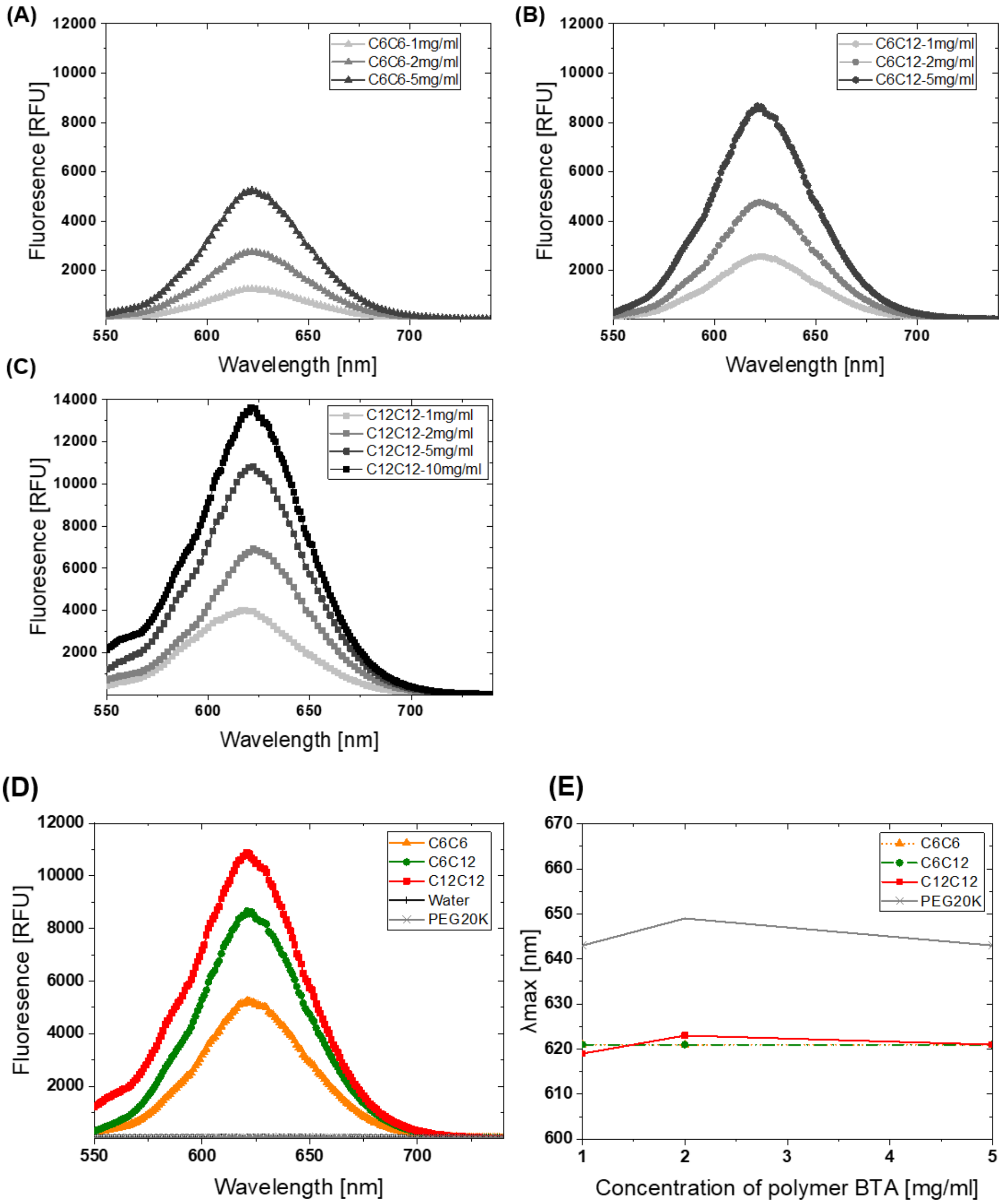

Figure S50: Nile red of hydrogelator (A) $\mathbf{C_6C_6}$, B) $\mathbf{C_6C_{12}}$, and C) $\mathbf{C_{12}C_{12}}$ at concentration 1 mg/mL, 2 mg/mL and 5 mg/mL. D) Nile red encapsulation shows an increase in fluorescence intensity from the C6C6 to C12C12 hydrogelators (5 mg/ml, 230 µM), B) The lambda max (λ_{max}) of encapsulated Nile Red at 1, 2, and 5 mg/ml showed minimal changes across the hydrogel series, suggesting a similar strength of hydrophobic environment.

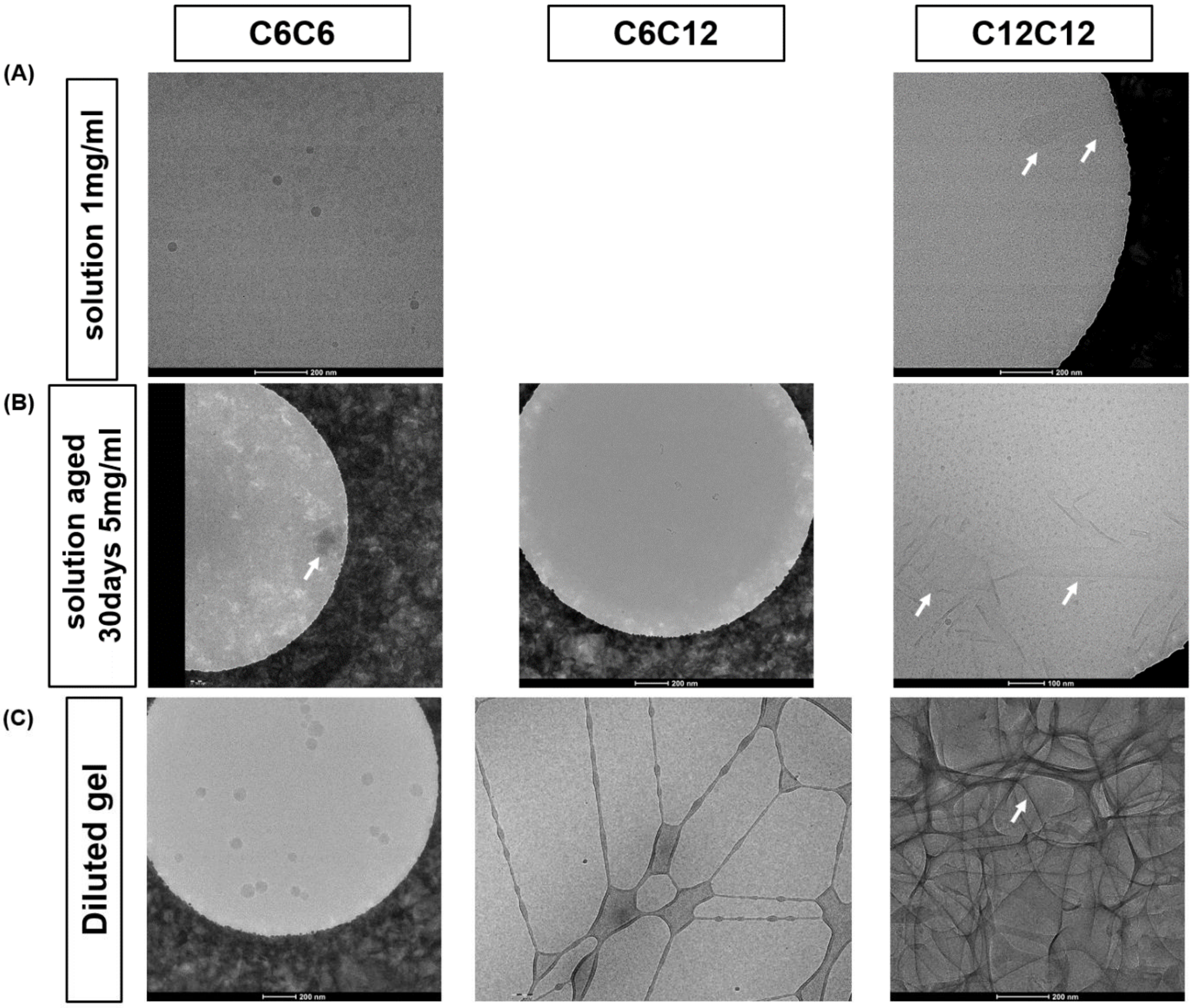

Figure S51: A) Cryo-TEM image of BTA hydrogelators at A) 1mg/ml and B) 5mg/ml self-assembled in aqueous solution. C) Cryo-TEM images of BTA hydrogelators when hydrogels at 10% (w/v) were diluted until they become clear solutions.

Critical gelation concentration (CGC):

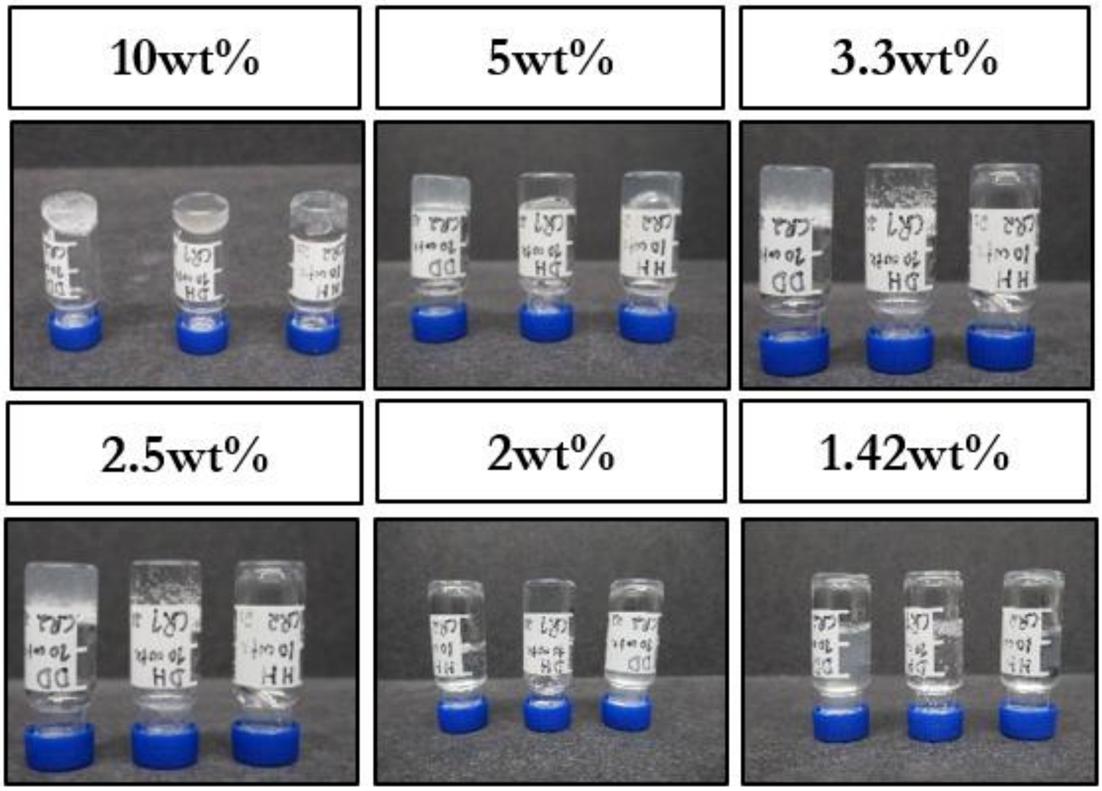

Figure S52: Critical gelation concentration experiment: hydrogelator **C_6C_6**, **C_6C_{12}** and **$C_{12}C_{12}$** are labelled as HH, DH and DD. Hydrogelator **C_6C_6**, **C_6C_{12}** and **$C_{12}C_{12}$** showed CGC ~5% (w/v), 1.42% (w/v) and ~2.5% (w/v). Vial was inverted at each wt% and observed if gel will flow or stay stable on top of the inverted vial. Observing or waiting time of the inverted vial to determine CGC was maximum 30 seconds.

Vial inversion experiment:

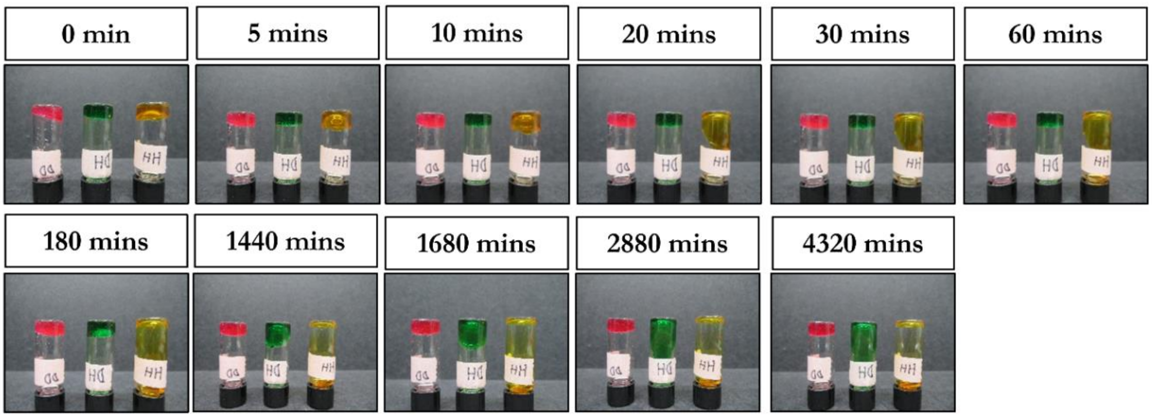

Figure S53: Vial inversion experiment to determine flow behaviour of hydrogels at 10% (w/v). Hydrogelator $\mathbf{C_6C_6}$, $\mathbf{C_6C_{12}}$ and $\mathbf{C_{12}C_{12}}$ are labelled as HH (orange), DH (green) and DD (red). Flow behavior is qualitative test and provide information on viscoelastic behaviour of hydrogels. Hydrogelator $\mathbf{C_6C_6}$ (HH) roughly started to flow after 20 minutes of vial inversion. A small flow can be seen for hydrogelator $\mathbf{C_6C_{12}}$ after 1680 minutes (28 hours) and hydrogelator $\mathbf{C_{12}C_{12}}$ did not flow even after 4320 minutes (72 hours).

Mechanical Properties:

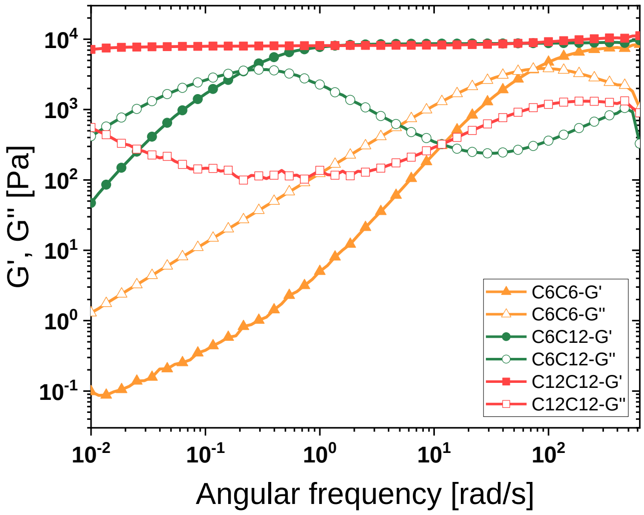

Figure S54: All hydrogelator showed frequency dependent loss moduli (G”) which show that hydrogelators are viscoelastic

Self-healing and moldability:

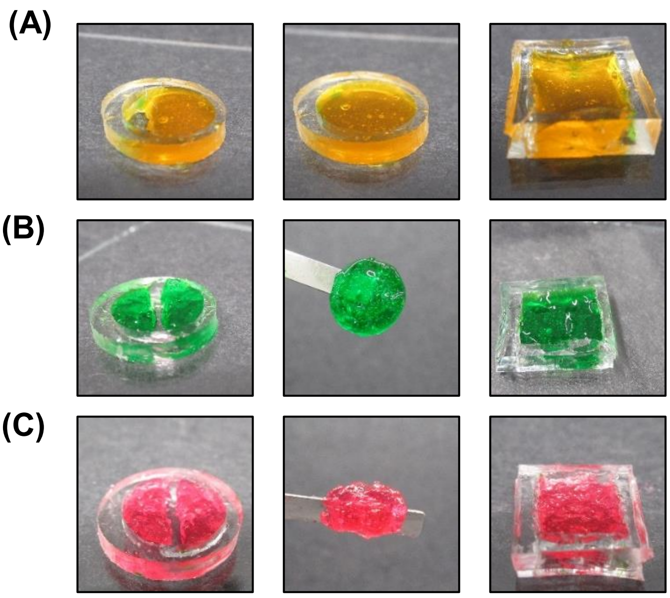

Figure S55: Self-healing and moldability of hydrogelators **C_6C_6** (orange), **C_6C_{12}** (green), and, **$C_{12}C_{12}$** (red). All hydrogelators are self-healing instantaneously when hydrogels pieces are placed and pressed together with spatula. Also hydrogels are moldable since they can adopt to different mould shapes when subjected to stress.

Cell viability:

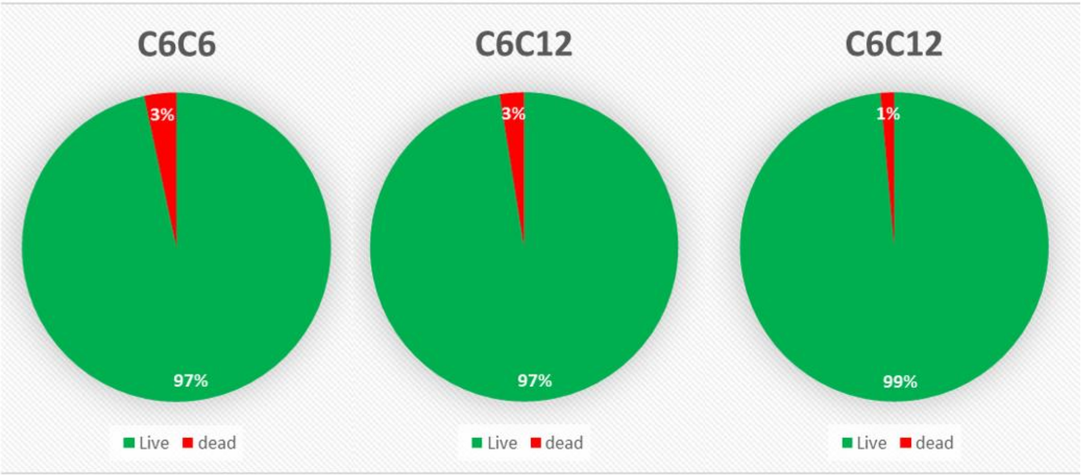

Figure S56: Cell viability of hydrogelators: Percentage of live and dead cells on day 4 in BTA hydrogels. ATDC5 forms multicellular aggregates within hydrogels and cell areas of multicellular aggregates were calculated and not of single cells. Live-dead cell area was calculated using Image J by stacking z stacks live and dead channel images were processed separately for calculating % of cell areas. The number of aggregates counted were between 190 and 500 for all BTA hydrogelators.

Bibliography

(1) Hendrikse, S. I. S.; Su, L.; Hogervorst, T. P.; Lafleur, R. P. M.; Lou, X.; Van Der Marel, G. A.; Codee, J. D. C.; Meijer, E. W. Elucidating the Ordering in Self-Assembled Glycocalyx Mimicking Supramolecular Copolymers in Water. *J. Am. Chem. Soc.* **2019**, *141* (35), 13877–13886. https://doi.org/10.1021/jacs.9b06607.

(2) Alex D. Beck. Density-Functional Thermochemistry. III. The Role of Exact Exchange. *J. Chem. Phys.* **1993**, *98* (7), 5648–5656.

(3) Stephen, P. J.; Devlin, F. J.; Chabalowski, C. F.; Frisch, M. J. Ab Initio Calculation of Vibrational Absorption. *J. Phys. Chem.* **1994**, *98* (45), 11623–11627.

(4) Petersson, G. A.; Tensfeldt, T. G.; Montgomery, J. A. A Complete Basis Set Model Chemistry. III. The Complete Basis Set-quadratic Configuration Interaction Family of Methods. *J. Chem. Phys.* **1991**, *94* (9), 6091–6101. https://doi.org/10.1063/1.460448.

(5) Clark, T.; Chandrasekhar, J.; Spitznagel, G. W.; von Ragué Schleyer, P. Efficient Diffuse Function- Augmented Basis Sets for Anion Calculations. III. The 3-21+G Basis Set for First-row Elements, Li–F. *J. Comput. Chem.* **1983**, *4* (3), 294-.

(6) Reed, A. E.; Curtiss, L. A.; Weinhold, F. Intermolecular Interactions from a Natural Bond Orbital, Donor—Acceptor Viewpoint. *Chem. Rev.* **1988**, *88* (6), 899–926. https://doi.org/10.1021/cr00088a005.

(7) Chattaraj, P. K.; Sarkar, U.; Roy, D. R. Electrophilicity Index. *Chem. Rev.* **2006**, *106* (6), 2065–2091. https://doi.org/10.1021/cr040109f.

(8) Ralph G. Pearson. Absolute Electronegativity and Hardness: Application to Inorganic Chemistry. *Inorg. Chem.* **1988**, *27* (4), 734–740.
